# An Omicron-specific, self-amplifying mRNA booster vaccine for COVID-19: a phase 2/3 randomized trial

**DOI:** 10.1038/s41591-024-02955-2

**Published:** 2024-04-18

**Authors:** Amit Saraf, Rohan Gurjar, Swarnendu Kaviraj, Aishwarya Kulkarni, Durgesh Kumar, Ruta Kulkarni, Rashmi Virkar, Jayashri Krishnan, Anjali Yadav, Ekta Baranwal, Ajay Singh, Arjun Raghuwanshi, Praveen Agarwal, Laxman Savergave, Sanjay Singh, the GEMCOVAC-OM Study Investigators, Himanshu Pophale, Prakash Shende, Ravindra Baban Shinde, Vikram Vikhe, Abhishek Karmalkar, Bhaskar Deshmukh, Krishna Giri, Shrikant Deshpande, Ajay Bulle, Md. Sabah Siddiqui, Swapnav Borthakur, V. Reddy Tummuru, A. Venkateshwar Rao, Dhaiwat Shukla, Manish Kumar Jain, Pankaj Bhardwaj, Pravin Dinkar Supe, Manoja Kumar Das, Manoj Lahoti, Vijaykumar Barge

**Affiliations:** 1 Gennova Biopharmaceuticals Limited, Pune, India; 2 Department of Communicable Diseases, Interactive Research School for Health Affairs, Bharati Vidyapeeth (Deemed to Be University), Pune, India; 3 JSS Medical Research, Haryana, India; 4 Cytel, Pune, India; 5 Ace Hospital and Research Centre, Pune, India; 6 Lokamanya Medical Research Centre, Pune, India; 7 CIMETs Inamdar Multispeciality Hospital, Pune, India; 8 Dr. D. Y. Patil Medical College Hospital and Research Centre, Pune, India; 9 Vedant Multispeciality Hospital, Pune, India; 10 Baramati Hospital, Pune, India; 11 Dhadiwal Hospital in coalition with Shreeji Healthcare, Nashik, India; 12 Ashirwad Hospital and Research Centre, Ulhasnagar, India; 13 Meditrina Institute of Medical Sciences, Nagpur, India; 14 All India Institute of Medical Sciences, Raipur, India; 15 Downtown Hospital Ltd, Guwahati, India; 16 Induss Hospital, Hyderabad, India; 17 St Theresa’s Hospital, Hyderabad, India; 18 V S General Hospital, Ahmedabad, India; 19 Maharaja Agrasen Super Specialty Hospital, Jaipur, India; 20 All India Institute of Medical Sciences, Jodhpur, India; 21 Supe Heart & Diabetes Hospital and Research Centre, Nashik, India; 22 SRMSIMS Hospital, Bareilly, India; 23 Suyash Institute of Medical Sciences Pvt Ltd, Raipur, India; 24 Rajarshee Chhatrapati Shahu Maharaj Government Medical College and Chhatrapati Pramila Raje Hospital, Kolhapur, India

**Keywords:** Randomized controlled trials, RNA vaccines

## Abstract

Here we conducted a multicenter open-label, randomized phase 2 and 3 study to assess the safety and immunogenicity of a severe acute respiratory syndrome coronavirus 2 (SARS-CoV-2) Omicron-specific (BA.1/B.1.1.529), monovalent, thermostable, self-amplifying mRNA vaccine, GEMCOVAC-OM, when administered intradermally as a booster in healthy adults who had received two doses of BBV152 or ChAdOx1 nCoV-19. GEMCOVAC-OM was well tolerated with no related serious adverse events in both phase 2 and phase 3. In phase 2, the safety and immunogenicity of GEMCOVAC-OM was compared with our prototype mRNA vaccine GEMCOVAC-19 (D614G variant-specific) in 140 participants. At day 29 after vaccination, there was a significant rise in anti-spike (BA.1) IgG antibodies with GEMCOVAC-OM (*P* < 0.0001) and GEMCOVAC-19 (*P* < 0.0001). However, the IgG titers (primary endpoint) and seroconversion were higher with GEMCOVAC-OM (*P* < 0.0001). In phase 3, GEMCOVAC-OM was compared with ChAdOx1 nCoV-19 in 3,140 participants (safety cohort), which included an immunogenicity cohort of 420 participants. At day 29, neutralizing antibody titers against the BA.1 variant of SARS-CoV-2 were significantly higher than baseline in the GEMCOVAC-OM arm (*P* < 0.0001), but not in the ChAdOx1 nCoV-19 arm (*P* = 0.1490). GEMCOVAC-OM was noninferior (primary endpoint) and superior to ChAdOx1 nCoV-19 in terms of neutralizing antibody titers and seroconversion rate (lower bound 95% confidence interval of least square geometric mean ratio >1 and difference in seroconversion >0% for superiority). At day 29, anti-spike IgG antibodies and seroconversion (secondary endpoints) were significantly higher with GEMCOVAC-OM (*P* < 0.0001). These results demonstrate that GEMCOVAC-OM is safe and boosts immune responses against the B.1.1.529 variant. Clinical Trial Registry India identifier: CTRI/2022/10/046475.

## Abstract (web summary)

A self-amplifying mRNA vaccine shows promise in this new modality by eliciting neutralizing antibodies against the SARS-CoV-2 Omicron (BA.1) variant in a phase 2/3 trial.

## Main

As of 3 March 2024, there have been 774,834,251 confirmed cases of COVID-19 with 7,037,007 deaths^1^. Vaccines designed for SARS-CoV-2 have been effective in mitigating the COVID-19 pandemic^2^.

Various platforms have been used to develop COVID-19 vaccines. These include inactivated whole virion, protein subunit and adenoviral vector platforms. However, one of the most prominent achievements during this pandemic has been the approval of messenger RNA-based vaccines for the first time for any disease.

mRNA vaccines have substantial advantages over the traditional vaccines. mRNA does not integrate into the host DNA and is noninfectious. Furthermore, mRNA vaccines are produced synthetically in a cell-free environment allowing a scalable, cost-effective and rapid production^3^. This makes the platform ideal to target emerging variants of concern, an important advantage given the rapidly evolving nature of SARS-CoV-2. Consequently, the first Omicron-specific adapted vaccines were mRNA based.

Notwithstanding these advantages, access to mRNA-based vaccines has been a challenge, especially for low- to middle-income countries (LMICs). The transport and storage of mRNA vaccines require a subzero temperature cold chain. Such infrastructure is not readily available in LMICs and is costly to implement.

The BA.1 (B.1.1.529) Omicron variant, first identified in Botswana, became dominant worldwide quickly and was seen to evade immunity acquired from vaccines that were designed against the ancestral strain^4^. An Omicron-adapted vaccine was needed to provide protection against this variant. To cater to this unmet need, we developed GEMCOVAC-OM, a monovalent, Omicron-specific (BA.1) booster mRNA vaccine for COVID-19 (ref. ^5^). It is distinct from the other two US Food and Drug Administration (FDA)-approved mRNA vaccines (from Pfizer-BioNTech and Moderna) in several ways. First, GEMCOVAC-OM is a self-amplifying mRNA (samRNA) vaccine, which can lower the dose for administration^6^. In contrast to the nonamplifying mRNA, the open reading frame of a samRNA encodes a replicase that is a complex of four nonstructural proteins (nsP1–nsP4). These nsPs interact to form an RNA-dependent RNA polymerase (RdRp) that drives the self-amplification of the mRNA inside the cell^7^. Second, GEMCOVAC-OM is lyophilized and stable at 2–8 °C for 12 months. Third, GEMCOVAC-OM is delivered intradermally through a needle-free injection system using a device called Tropis (PharmaJet).

In this Article, we describe the results of a phase 2 and 3 study, designed to assess the safety and immunogenicity of GEMCOVAC-OM as a heterologous booster in healthy adults (18 years of age and older). GEMCOVAC-OM has received an Emergency Use Authorization from the Central Licensing Authority in India on 19 June 2023. We present the immunogenicity and safety results from the phase 2 and 3 study.

## Results

GEMCOVAC-OM is a SARS-CoV-2 Omicron-specific (B.1.1.529), monovalent, thermostable, samRNA vaccine. Details on the self-amplifying nature of GEMCOVAC-OM and the methods used for its characterization and production can be found in Supplementary Information.

### Study design

We conducted a multicenter, randomized phase 2 seamlessly followed by phase 3 study to assess the safety and immunogenicity of GEMCOVAC-OM in healthy adults who had received two doses of either BBV152 (COVAXIN) or ChAdOx1 nCoV-19 (COVISHIELD) as their primary vaccination at least 4 months before screening. In the phase 2 study, 140 participants were randomized to either GEMCOVAC-OM or the prototype vaccine GEMCOVAC-19, which was designed against the D614G variant of SARS-CoV-2. Primary endpoints of phase 2 were to compare the safety and anti-spike IgG antibodies between the two vaccinated arms at a prespecified interim analysis at day 29 post-vaccination. Secondary endpoints included comparison of seroconversion as assessed by a ≥2-fold rise in anti-spike IgG antibody titers from baseline, percentage neutralization by a surrogate neutralization (cPass) assay and cellular immune responses at day 29. Additionally, exploratory endpoints included comparison of anti-spike IgG antibodies, percentage neutralization by cPass assay and cellular immune responses at day 90.

Ideally, in phase 3, GEMCOVAC-OM should have been compared with an Omicron-specific mRNA vaccine. However, at the time of the study, neither mRNA nor Omicron-specific vaccines were approved or available in India. Hence, in phase 3, we compared GEMCOVAC-OM with ChAdOx1 nCoV-19, which was designed against the Wuhan strain and was approved for administration as a booster in India. The primary endpoint of the study was to demonstrate the noninferiority of GEMCOVAC-OM to ChAdOx1 nCoV-19 in terms of neutralizing antibody titers assessed by least square geometric mean ratio (LSGMR) and difference in seroconversion at day 29. Secondary endpoints included comparison of anti-spike IgG antibody titers, percentage neutralization by cPass assay, cellular immune responses at day 29 and safety for the duration of the study (day 180). Additionally, exploratory endpoints were comparison of humoral and cellular immune responses at day 90.

### Participants

**Fig. 1:**
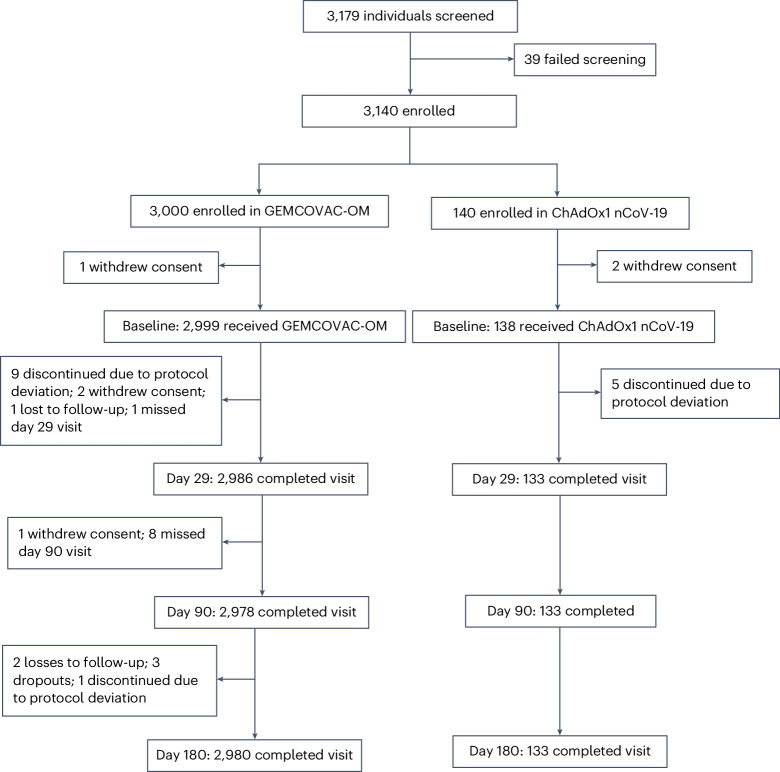
**CONSORT diagram**.

In phase 2, a total of 140 participants received GEMCOVAC-19 and GEMCOVAC-OM in a 1:1 ratio between 18 October 2022 and 20 October 2022. All 140 individuals completed the study up to the day 180 follow-up. In phase 3, a total of 3,140 participants were enrolled from 15 November 2022 to 24 November 2022; 3,000 and 140 participants were enrolled in the GEMCOVAC-OM and the ChAdOx1 nCoV-19 arms, respectively. Of these, three participants withdrew their consent before vaccination. After vaccination on day 1, it was found that 14 participants (9 in GEMCOVAC-OM arm and 5 in ChAdOx1 nCoV-19 arm) had already received a booster vaccine for COVID-19. This was a major protocol deviation and, hence, these participants were excluded from the safety and immunogenicity analysis. Additionally, in the GEMCOVAC-OM group, two participants withdrew consent, one individuals was lost to follow-up and one participant missed the day 29 visit. The day 29 visit was completed by 3,119 participants of which 404 (271 in the GEMCOVAC-OM arm and 133 in ChAdOx1 nCoV-19 arm) were included in the primary immunogenicity endpoint. Subsequently, in the GEMCOVAC-OM group, one individual withdrew consent, eight participants missed their day 90 visit, two participants were lost to follow-up and three exited the study. There was one protocol deviation where it was revealed that, for one participant, the time between the second dose of the primary vaccination and the booster vaccination was less than 4 months (exclusion criteria), which led to the discontinuation of the participant from the study. The last visit of day 180 was completed by 2,980 participants in the GEMCOVAC-OM group and 133 participants in the ChAdOx1 nCoV-19 group (Fig. 1).

### Baseline and demographic characteristics

The median age of participants was comparable in both phase 2 (GEMCOVAC-OM: 32 years and GEMCOVAC-19: 30 years) and phase 3 (32 years in both GEMCOVAC-OM and ChAdOx1 nCoV-19 arms). Of note, there were considerably more men in both phase 2 (87.1% in GEMCOVAC-OM and 91.4% in GEMCOVAC-19) and phase 3 (68.2% in GEMCOVAC-OM and 79.7% in ChAdOx1 nCoV-19). There was no notable difference in the mean weight and body mass index of participants in both phase 2 and phase 3 (Table 1).

**Table 1:** Demography and baseline characteristics

Phase 2	GEMCOVAC-OM (*N* = 70)	GEMCOVAC-19 (*N* = 70)
Age, median years (range)	32 (20–49)	30 (20–45)
Gender		
Female	9 (12.9%)	6 (8.6%)
Male	61 (87.1%)	64 (91.4%)
Weight, mean in kg (s.d.)	64.8 (5.07)	63.7 (5.17)
Body mass index in kg m^−2^, mean (s.d.)	23.2 (1.67)	23.0 (1.54)

Phase 3	GEMCOVAC-OM (*N* = 2,990)	ChAdOx1 nCoV-19 (*N* = 133)
Age, median years (range)	32 (18–81)	32 (19–57)
Gender		
Female	951 (31.8%)	27 (20.3%)
Male	2,039 (68.2%)	106 (79.7%)
Weight, mean in kg (s.d.)	62.6 (10.10)	63.9 (10.63)
Body mass index in kg m^−2^, mean (s.d.)	23.6 (3.43)	23.8 (3.52)

### Humoral Immunogenicity

All immunogenicity assessments were conducted against the BA.1 Omicron variant of SARS-CoV-2. Since none of the ancestral strains (Wuhan and D614G) was in circulation, immunogenicity against them was not assessed.

In phase 2, there was a statistically significant rise in the geometric mean titers (GMT) of anti-spike IgG antibodies from baseline (30,048, 95% confidence interval (CI) 23,910–37,763) to day 29 (244,440, 95% CI 229,122–260,782, *P* < 0.0001) with GEMCOVAC-OM (Extended Data Table 1). Similar increase in anti-spike IgG antibodies from baseline (35,676, 95% CI 29,401–43,289) to day 29 (75,683, 95% CI 61,687–92,853, *P* < 0.0001) was observed with GEMCOVAC-19. The geometric mean fold rise (GMFR, post-booster/prebooster vaccination) in anti-spike IgG antibodies for GEMCOVAC-OM and GEMCOVAC-19 was 8.13 and 2.12, respectively. The LSGMR of anti-spike IgG antibodies (GEMCOVAC-OM/GEMCOVAC-19) calculated using analysis of covariance (ANCOVA) was 3.40 (95% CI 2.79–4.13, *P* < 0.0001). At day 29, more participants (92.9%) in the GEMCOVAC-OM group achieved a ≥2-fold rise (seroresponse) in antibody titers as compared with participants (57.1%) in the GEMCOVAC-19 group. The seroresponse rate difference (secondary endpoint) calculated using the Miettinen–Nurminen method was 35.71 (95% CI 22.35–48.52, *P* < 0.0001). Furthermore, at day 90, anti-spike IgG GMT in both GEMCOVAC-OM and GEMCOVAC-19 groups was higher than baseline. However, these titers were higher with GEMCOVAC-OM compared with GEMCOVAC-19 (LSGMR: 3.31, 95% CI 2.72–4.02, *P* < 0.0001). Change in mean percentage neutralization (secondary endpoint), assessed by the surrogate neutralization assay (cPass), from baseline to day 29, was higher with GEMCOVAC-OM (19.3, standard error (s.e.) 1.40) compared with GEMCOVAC-19 (8.4, s.e. 1.40, *P* < 0.0001). Additionally, at day 90, the mean percentage neutralization for GEMCOVAC-OM and GEMCOVAC-19 was 96.3 (s.d. 8.35) and 90.0 (s.d. 21.11), respectively (Supplementary Table 3).

**Fig. 2:**
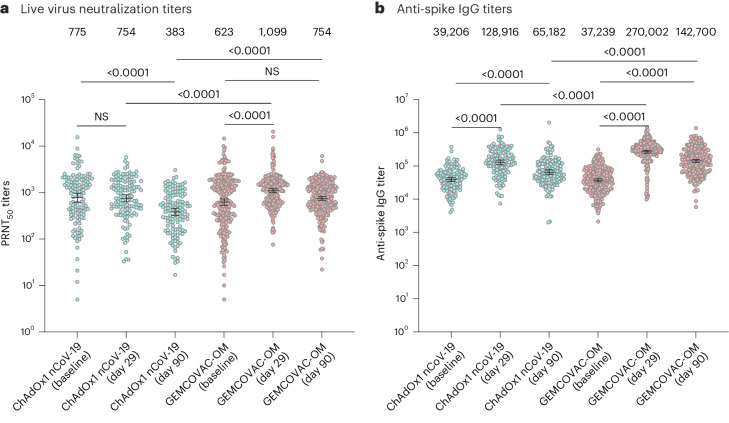
Humoral immune responses against Omicron B.1.1.529 variant. **a**,**b**, Humoral immune response with ChAdOx1 nCoV-19 (*n* = 133) and GEMCOVAC-OM (*n* = 271) assessed by PRNT_50_ (**a**) and anti-spike IgG antibodies (**b**). The data are presented as geometric mean with 95% CI. LSGMR with 95% CI at day 29 and day 90 along with the *P* value was calculated using ANCOVA with baseline values as covariates. Change in titers from baseline to day 29 and day 90 was calculated by using a two-sided paired *t*-test or Wilcoxon signed-rank test based on normality. NS, not significant.

In phase 3, there was a statistically significant rise in the GMT of neutralizing antibodies (PRNT_50_) against SARS-CoV-2 from baseline (623.9, 95% CI 533.3–729.9) to day 29 (1,099.9, 95% CI 1,000.0–1,209.9, *P* < 0.0001) with GEMCOVAC-OM. In contrast, no significant change in the GMT of neutralizing antibodies was observed with ChAdOx1 nCoV-19 from baseline (775.3, 95% CI 620.2–969.2) to day 29 (754.9, 95% CI 631.5–902.5, *P* = 0.1490; Fig. 2a). GMFR in neutralizing antibodies for GEMCOVAC-OM and ChAdOx1 nCoV-19 were 1.76 and 0.97, respectively. LSGMR of the neutralizing antibodies for the treatment groups (GEMCOVAC-OM/ChAdOx1 nCoV-19) at day 29 was 1.58 (95% CI 1.36–1.84; Table 2). The lower bound 95% CI of LSGMR was above the prespecified criteria of noninferiority (>0.67). At day 29, more participants in the GEMCOVAC-OM arm (39.5%) showed a seroresponse (≥2-fold rise in PRNT_50_) as compared with the participants in the ChAdOx1 nCoV-19 arm (19.5%; Table 2). The seroresponse rate difference between GEMCOVAC-OM and ChAdOx1 nCoV-19 was 19.93 (95% CI 10.57–28.43), which was statistically significant (*P* < 0.0001). The lower bound 95% CI of the difference in seroconversion was above the predefined criteria for noninferiority (>−10%). Moreover, the lower bound 95% CI for LSGMR and the lower bound 95% CI of difference in seroconversion were above the superiority criteria of >1 and >0%, respectively. Furthermore, at day 90, GMT of neutralizing antibodies was higher with GEMCOVAC-OM (754.0, 95% CI 682.0–833.6) compared with ChAdOx1 nCoV-19 (383.1, 95% CI 319.3–459.6; Fig. 2a). The GMFR from baseline with GEMCOVAC-OM (1.21) was numerically higher than ChAdOx1 nCoV-19 (0.49). LSGMR of the neutralizing antibodies at day 90 was 2.09 (95% CI 1.75–2.49, *P* < 0.0001; Table 2).

**Table 2:** Live virus neutralization assessed by PRNT_50_ in phase 3

	GEMCOVAC-OM (*N* = 271)	ChAdOx1 nCoV-19 (*N* = 133)
**Baseline** GMT (95% CI)^a^	623.9 (533.3–729.9)	775.3 (620.2–969.2)
**Day 29** GMT (95% CI)^a^	1,099.9 (1,000.0–1,209.9)	754.9 (631.5–902.5)
GMFR^b^	1.76	0.97
*P* value^c^	<0.0001	0.1490
LSGMR (GEMCOVAC-OM/ChAdOx1 nCoV-19) 95% CI^d^	1.58 (1.36–1.84)	
*P* value^d^	<0.0001	
Seroconversion assessed by ≥2-fold rise from baseline, % (95% CI)^e^	39.5 (33.6–45.5)	19.5 (13.1–27.3)
Difference in seroconversion (95% CI)	19.93 (10.5–28.4)^f^	
**Day 90** GMT (95% CI)^a^	754.0 (682.1–833.6)	383.1 (319.4–459.6)
GMFR^b^	1.21	0.49
*P* value^c^	0.5618	<0.0001
LSGMR (GEMCOVAC-OM/ChAdOx1 nCoV-19) 95% CI^d^	2.09 (1.75–2.49)	
*P* value^d^	<0.0001	

^a^The 95% CI of GMT was calculated by taking the log base 10 transformed titers.

^b^GMFR was calculated as post/pre of neutralizing antibody titers.

^c^*P* value for GMFR was calculated using a two-sided Wilcoxon signed-rank test.

^d^LSGMR (95% CI) and *P* value was calculated using ANCOVA with baseline values as covariates.

^e^The 95% CIs were calculated by the Clopper–Pearson method.

^f^Two-sided 95% CIs for difference in proportion of participants between groups were calculated using the Miettinen–Nurminen method.

In phase 3, there was an increase in the anti-spike IgG GMT from baseline to day 29 in both vaccinated groups (Fig. 2b). GMFR was numerically higher in GEMCOVAC-OM (7.25) compared with ChAdOx1 nCoV-19 (3.29). LSGMR of anti-spike IgG antibody titers (GEMCOVAC-OM/ChAdOx1 nCoV-19) at day 29 was 2.15 (95% CI 1.83–2.52, *P* < 0.0001). Seroresponse in terms of anti-spike IgG antibodies were higher with GEMCOVAC-OM (93.0%) compared with ChAdOx1 nCoV-19 (76.7%) with a difference of 16.30 (95% CI 9.02–24.64, *P* < 0.0001; Supplementary Table 6). Similarly, at day 90, anti-spike IgG GMT was significantly higher with GEMCOVAC-OM compared with ChAdOx1 nCoV-19 with a LSGMR of 2.23 (95% CI 1.87–2.66, *P* < 0.0001).

Mean percentage neutralization assessed by cPass assay was higher at day 29 (94.0%, s.d. 11.30) compared with baseline (68.1%, s.d. 27.07) with GEMCOVAC-OM. A similar increase was observed with ChAdOx1 nCoV-19 at day 29 (94.3%, s.d. 12.26) compared with baseline (68.6%, s.d. 26.10). There was no difference in the increase in the mean percentage neutralization between the two groups (*P* = 0.8559). However, at day 90, mean percentage neutralization was numerically higher with GEMCOVAC-OM (91.7%, s.d. 11.45) compared with ChAdOx1 nCoV-19 (81.3%, s.d. 19.74; Supplementary Table 7).

We conducted a subgroup analysis of the humoral immunogenicity data based on primary vaccination in both phase 2 and phase 3 and observed that the differences in immune responses between GEMCOVAC-OM and the comparators were consistent. Additionally, we assessed the humoral response (PRNT and IgG titers) and safety of phase 3 using data disaggregated by sex. In both the vaccine arms, women had numerically higher neutralizing and anti-spike IgG antibody titers at baseline compared with men. We considered these baseline titers as covariates in the ANCOVA model that was used to assess differences in the humoral immunogenicity in male and female participants. In the ChAdOx1 nCoV-19 arm, there were no significant differences between the two sexes in the neutralizing and anti-spike IgG titers at day 29 and day 90. Similarly, in the GEMCOVAC-OM group, there were no significant difference in the neutralizing antibodies at day 29 and anti-spike IgG antibodies at days 29 and 90 between female and male participants. However, at day 90, neutralizing antibody titers were significantly higher in women compared with men who received GEMCOVAC-OM (LSGMR: 0.79, 95% CI 0.63–0.98, *P* = 0.0333; Supplementary Figs. 9 and 10).

### Cell-mediated immunogenicity

Cellular responses against the B.1.1.529 (BA.1) variant were assessed by stimulating peripheral blood mononuclear cells (PBMCs) with an Omicron-specific peptide pool. Cellular immunogenicity assessed at day 29 was the secondary objective for both phase 2 and phase 3 part of the study. The cellular response at day 90 was a part of the exploratory endpoints.

In phase 2, the cellular immune response was assessed in 20% of the participants (14 in each arm). Lymphocyte counts were comparable in both the vaccinated groups (Supplementary Fig. 2). A statistically significant increase in IFNγ^+^CD4^+^ T cells from baseline to day 29 was observed in participants who received GEMCOVAC-19 (*P* = 0.0076) and GEMCOVAC-OM (*P* = 0.007). A similar increase was also observed in IL-2^+^CD4^+^ T cells in participants who received GEMCOVAC-19 (*P* = 0.0005) and GEMCOVAC-OM (*P* < 0.0001). At day 29, IFNγ^+^CD4^+^ T cells and IL-2^+^CD4^+^ T cells were comparable in GEMCOVAC-19 and GEMCOVAC-OM (Supplementary Fig. 3). In terms of CD8^+^ T cells, GEMCOVAC-19 and GEMCOVAC-OM showed a significant increase in IFNγ^+^CD8^+^ T cells (*P* = 0.0004 and *P* = 0.0009 respectively) and IL-2^+^CD8^+^ T cells (*P* = 0.0002 and *P* = 0.0001, respectively) from baseline to day 29. Moreover, a significant increase in TNF^+^CD8^+^ T cells from baseline to day 29 was observed in GEMCOVAC-OM (*P* = 0.002; Supplementary Fig. 4). Additionally, at day 90, GEMCOVAC-19 and GEMCOVAC-OM had significantly higher IFNγ^+^CD4^+^ T cells (*P* = 0.001 and *P* = 0.0465, respectively) and IFNγ^+^CD8^+^ T cells (*P* = 0.0002 and *P* = 0.0027, respectively) compared with baseline. TNF^+^CD8^+^ T cells were higher with GEMCOVAC-OM (*P* = 0.0003) at day 90 compared with baseline. At day 90, TNF^+^CD4^+^ T cells (*P* = 0.0055) and IL-2^+^CD8^+^ T cells (*P* = 0.0271) were higher with GEMCOVAC-OM compared with GEMCOVAC-19. Spike-specific T helper 2 (T_H_2) cell cytokine (IL-4 and IL-13) expression in the T cells from both the vaccinated cohorts was significantly lower at day 29 and day 90 compared to baseline (Supplementary Fig. 5).

**Fig. 3:**
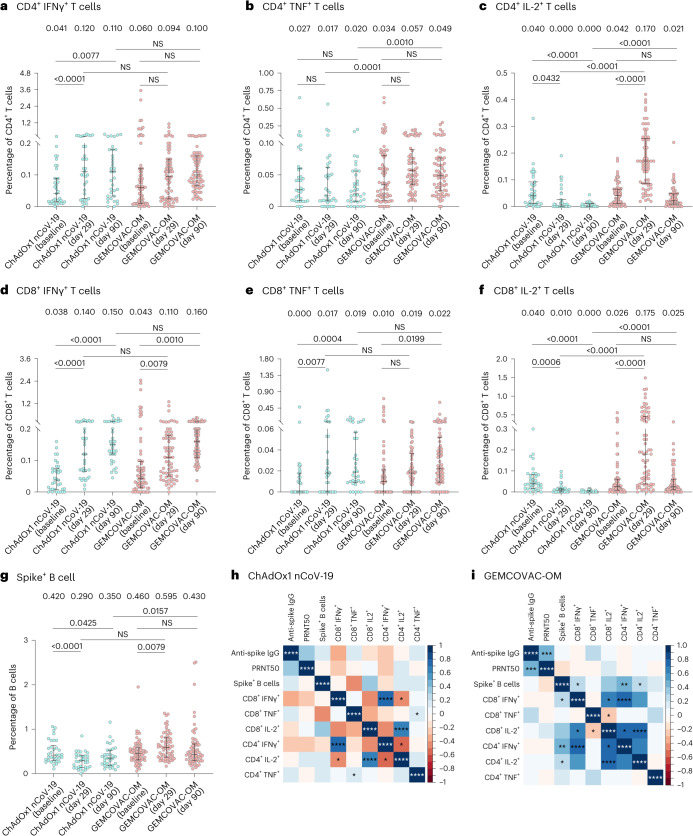
Cellular immune responses against Omicron B.1.1.529 variant. **a**–**f**, For T cell response analysis, PBMCs from ChAdOx1 nCoV-19 (*n* = 35) and GEMCOVAC-OM (*n* = 71) cohorts were stimulated with Omicron spike-specific PepTivator. Change in CD4^+^ T cells expressing IFNγ (**a**), TNF (**b**) and IL-2 (**c**) and CD8^+^ T cells expressing IFNγ (**d**), TNF (**e**) and IL-2 (**f**). **g**, Omicron B.1.1.529 spike^+^ CD19^+^ CD20^+^ B cells in ChAdOx1 nCoV-19 (*n* = 34) and GEMCOVAC-OM (*n* = 67) cohorts. The data are presented as median with interquartile range. Change in cytokine expression from baseline to day 29 and day 90 was assessed using a two-sided paired *t*-test or Wilcoxon signed-rank test based on normality. Expression at day 29 and day 90 in both the groups was compared using a two-sided *t*-test or Wilcoxon rank sum test based on normality. **h**,**i**, Spearman correlation between humoral and cellular responses in ChAdOx1 nCoV-19 (**h**) and GEMCOVAC-OM (**i**) cohorts measured at day 29. In corrplot, the blue boxes represent positive correlations and the red boxes represents negative correlation. Significant correlations were represented as an asterisk in the boxes. *P < 0.05, **P < 0.01, ***P < 0.001 and ****P < 0.0001.

**Extended Data Fig. 1:**
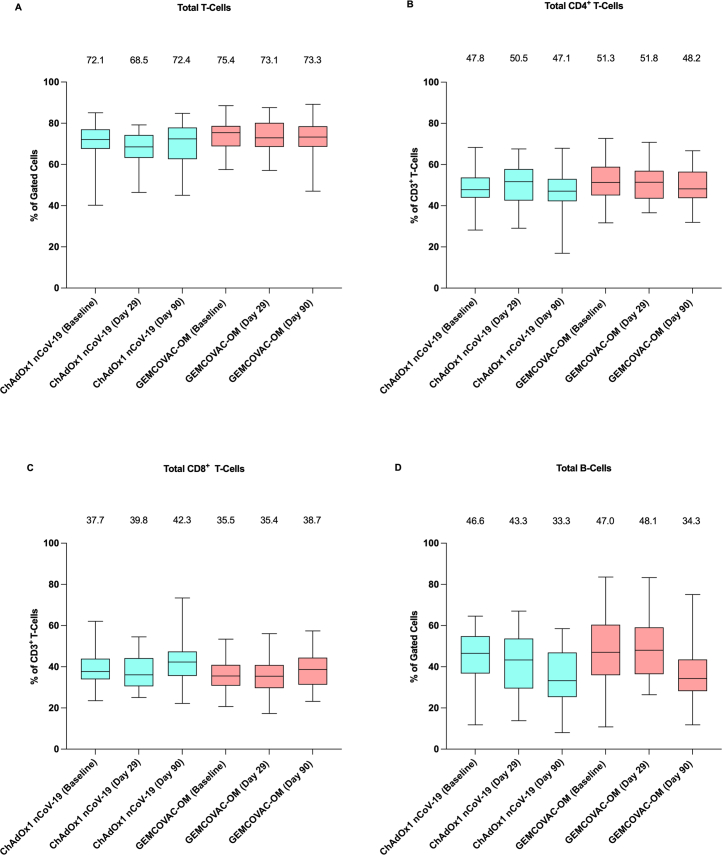
Lymphocytes populations in ChAdOx1 nCoV-19 and GEMCOVAC-OM cohorts. (**A**) Total T-cells (CD3^+^ cells) population in % of lymphocyte gate applied, (**B**) total CD4^+^ T-cells population in % of total CD3^+^ T-cells, (**C**) total CD8^+^ T-cells in % of total CD3^+^ T-cells in ChAdOx1 nCoV-19 (n = 35) and GEMCOVAC-OM (n = 71) cohorts. (**D**) Total CD19^+^ CD20^+^ B-cells in % of CD3^−^ gated population in ChAdOx1 nCoV-19 (n = 34) and GEMCOVAC-OM (n = 67) cohorts. Box plots represent the median, 25^th^ and 75^th^ percentiles. Whiskers extend from the minimum to maximum values in the data set. Change in T-cells from baseline to day 29 and day 90 was assessed using a two-sided paired t-test or Wilcoxon sign ranked test based on normality. Expression at day 29 and day 90 in both the groups was compared using a two-sided t-test or Wilcoxon rank sum test based on normality.

In the phase 3 study, cellular immunity was assessed in subset of participants (~25% from each arm). Hence, a total of 106 samples (GEMCOVAC-OM: 71 and ChAdOx1 nCoV-19: 35) were included in the analysis. Lymphocyte counts were comparable across both the vaccinated groups (Extended Data Fig. 1a–c). There was a statistically significant increase in IFNγ^+^CD4^+^ T cells from participants vaccinated with ChAdOx1 nCoV-19 (*P* < 0.0001) and in IL-2^+^CD4^+^ T cells from recipients of GEMCOVAC-OM (*P* < 0.0001) from baseline to day 29. However, at day 29, there was no significant difference in the IFNγ^+^CD4^+^ T cells in both the groups. GEMCOVAC-OM samples showed significantly higher TNF^+^CD4^+^ and IL-2^+^CD4^+^ T cells compared with ChAdOx1 nCoV-19 (*P* < 0.0001; Fig. 3a–c) at day 29. With regard to cytotoxic T cells, a significant rise in IFNγ^+^CD8^+^ T cells was seen with both ChAdOx1 nCoV-19 (*P* < 0.0001) and GEMCOVAC-OM (*P* = 0.0079). ChAdOx1 nCoV-19 samples showed a significant increase in TNF ^+^CD8^+^ T cells from baseline to day 29. However, at day 29, there was no difference in TNF ^+^CD8^+^ T cells in GEMCOVAC-OM and ChAdOx1 nCoV-19. GEMCOVAC-OM samples had a significantly higher percentage of IL-2^+^CD8^+^ T cells at day 29 compared with baseline (*P* < 0.0001). The percentage of IL-2^+^CD8^+^ T cells was also higher than ChAdOx1 nCoV-19 samples at day 29 (*P* < 0.0001; Fig. 3d–f). Additionally, at day 90, the percentage of IFNγ^+^CD4^+^, IFNγ^+^CD8^+^ and TNF^+^CD8^+^ T cells were higher in ChAdOx1 nCoV-19, whereas IFNγ^+^CD8^+^ and TNF^+^CD8^+^ T cells were higher in GEMCOVAC-OM compared with baseline. GEMCOVAC-OM showed significantly higher Omicron B.1.1.529-reactive TNF^+^CD4^+^ (*P* < 0.001), IL-2^+^CD4^+^ (*P* < 0.0001) and IL-2^+^CD8^+^ (*P* < 0.0001) T cells compared with ChAdOx1 nCoV-19 (Fig. 3a–f).

**Extended Data Fig. 2:**
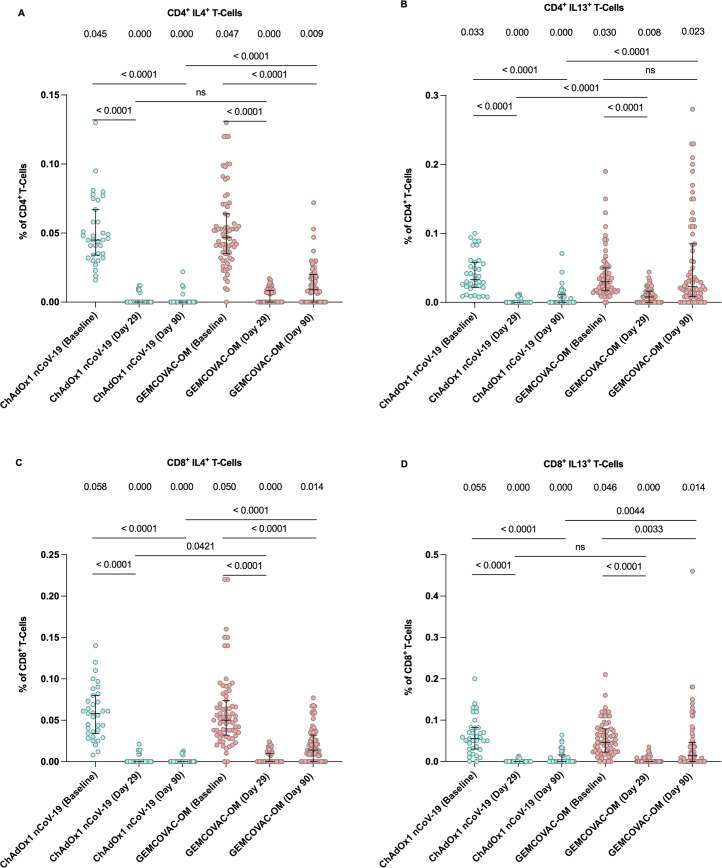
Th2 response in ChAdOx1 nCoV-19 and GEMCOVAC-OM cohorts. PBMCs were stimulated with Omicron Spike-specific PepTivator^®^. (**A**) IL-4 expressing CD4^+^ T-cells, (**B**) IL-13 expressing CD4^+^ T-cells, (**C**) IL-4 expressing CD8^+^ T-cells and (**D**) IL-13 expressing CD8^+^ T-cells in ChAdOx1 nCoV-19 (n = 35) and GEMCOVAC-OM (n = 71) cohorts. Data is presented as median with interquartile range (IQR). Change in cytokine expression from baseline to day 29 and day 90 was assessed using a two-sided paired t-test or Wilcoxon sign ranked test based on normality. Expression at day 29 and day 90 in both the groups was compared using a two-sided t-test or Wilcoxon rank sum test based on normality. ns = not significant.

B.1.1.529 spike-specific T_H_2 cell cytokine expressions (IL-4 and IL-13) in T cells in both phase 2 and phase 3 study were lower at day 29 and day 90 compared with their baselines (Extended Data Fig. 2).

B.1.1.529 spike-specific B cells following booster dose were also quantified. Total CD19^+^CD20^+^ B cells were comparable in both the arms at day 29 (Extended Data Fig. 1d). GEMCOVAC-OM displayed a significant increase in B.1.1.529 spike-specific B cells compared with baseline (*P* = 0.0013) and ChAdOx1 nCoV-19 at day 29 (*P* < 0.0001; Fig. 3g). However, at day 90, Omicron B.1.1.529-specific B cells in the GEMCOVAC-OM study group were similar to baseline level but significantly higher than ChAdOx1 nCoV-19 (*P* = 0.0157).

A post-hoc Spearman correlation analysis was conducted to elucidate the relationships between immunogenic responses measured on day 29 with GEMCOVAC-OM and ChAdOx1 nCoV-19 (Fig. 3h,i). A significant positive correlation between anti-spike IgG (binding) antibody titers and neutralizing antibody titers was observed with GEMCOVAC-OM, but not with ChAdOx1 nCoV-19. In both, GEMCOVAC-OM and ChAdOx1 nCoV-19 cohorts, a strong positive correlation was seen between the IFNγ^+^CD4^+^ and IFNγ^+^CD8^+^ T cells (*P* < 0.0001). ChAdOx1 nCoV-19 also showed a significant positive correlation between TNF-expressing CD4^+^ and CD8^+^ T cells (*P* < 0.05) as well as IL-2^+^CD4^+^ and IL-2^+^CD8^+^ T cells (*P* < 0.0001). Specifically, a noteworthy finding was the significant positive correlation between spike^+^ B cells and IFNγ^+^CD4^+^, IFNγ^+^CD8^+^ and IL-2^+^CD4^+^ T cells with GEMCOVAC-OM. Significant positive correlations between IL-2-expressing CD4^+^ and CD8^+^ T cells (*P* < 0.0001) as well as IFNγ^+^CD8^+^ and IL-2^+^CD8^+^ T cells (*P* < 0.05) were also observed with GEMCOVAC-OM.

### Safety

Safety was the co-primary endpoint in phase 2 and secondary endpoint in phase 3. As this was a seamless study, the adverse event (AE) data 7 days post vaccination in phase 2 was analyzed and presented to the Data Safety Monitoring Board (DSMB). After approval by the DSMB, phase 3 was initiated. Safety data of both phase 2 and phase 3 study were reviewed periodically by DSMB.

In phase 2, there were a total of 14 AEs of which 5 occurred in participants who received GEMCOVAC-OM and 9 in those who received GEMCOVAC-19 (Supplementary Table 4). No unsolicited events or serious AEs were reported till the end of the study.

**Fig. 4:**
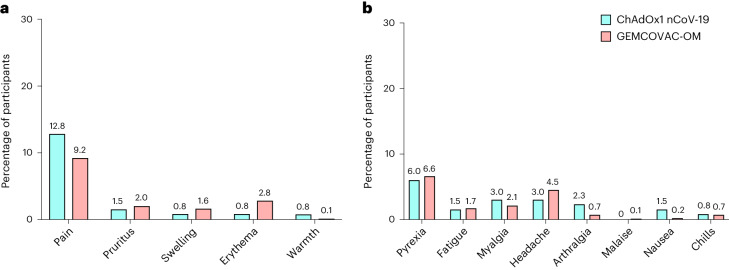
Solicited local and systemic AEs. **a**,**b**, Percentage of participants in whom local (**a**) and systemic (**b**) solicited AEs were observed in the first 7 days after a booster dose of ChAdOx1 nCoV-19 (*n* = 133) and GEMCOVAC-OM (*n* = 2,990).

In phase 3, no substantial difference in the AEs was observed in participants who received GEMCOVAC-OM and ChAdOx1 nCoV-19 (Fig. 4). In the GEMCOVAC-OM arm, 602 participants (20.1%) reported at least one AE compared with 33 (24.8%) in the ChAdOx1 nCoV-19 arm. Most of the reported AEs were mild. In the GEMCOVAC-OM arm, the most common local solicited AE was injection site pain (9.2%), followed by erythema (2.8%), pruritus (2.0%) and swelling (1.6%). The most common systemic solicited AE was fever (6.6%), followed by headache (4.5%), myalgia (2.1%), fatigue (1.7%), arthralgia (0.7%), chills (0.7%) and nausea (0.2%). A total 1.40% of participants in GEMCOVAC-OM and 2.26% in ChAdOx1 nCoV-19 reported unsolicited events until day 180. Three participants from the GEMCOVAC-OM arm reported serious AEs of omphalitis, spontaneous abortion and pulmonary tuberculosis, while one participant who received ChAdOx1 nCoV-19 suffered a ligament rupture. These events were determined unlikely due to vaccination by the study site investigators. There was no death in the study. In phase 3, there was no notable difference in the AEs reported between men and women who received ChAdOx1 nCoV-19. However, in the GEMCOVAC-OM group, more women than men reported local (unadjusted odds ratio (OR) 0.78, 95% CI 0.64–0.96) and systemic (OR 0.73, 95% CI 0.60–0.90) AEs (Supplementary Table 11).

## Discussion

We report the humoral and cellular immunogenicity as well as the safety results of the phase 2 and 3 study of GEMCOVAC-OM versus GEMCOVAC-19 and ChAdOx1 nCoV-19, respectively, administered as a heterologous booster in participants who received either BBV152 or ChAdOx1 nCoV-19 as their primary vaccination. GEMCOVAC-OM was found to be safe and well tolerated and generated significant humoral as well as cellular immune responses against the B.1.1.529 variant. GEMCOVAC-OM is thermostable and the first samRNA vaccine to receive an Emergency Use Authorization. Moreover, it is the only Omicron-specific vaccine that is approved in India.

Recently, a samRNA vaccine (ARCT-154) by Arcturus Therapeutics and CSL received a full approval as a COVID-19 booster in Japan^8^. This approval was based on a phase 3 study in which ARCT-154 administered at 5 μg dose resulted in higher immune responses than 30 μg Comirnaty (Pfizer/BioNTech)^9^. Several companies across the globe^10,11^ are developing samRNA vaccine candidates against COVID-19 that are already in clinical trials.

In phase 2 and phase 3, the safety and tolerability of GEMCOVAC-OM was comparable to GEMCOVAC-19 and ChAdOx1 nCoV-19. Most of the AEs were mild to moderate and resolved on their own. No vaccine-related serious AEs or deaths were reported. mRNA vaccines have been associated with myocarditis, and this AE was included in the study trial as an AE of special interest. None of the participants reported any signs or symptoms of myocarditis that would warrant further investigation, which is expected given the incidence of 1–5 cases per 100,000 in the general population^12^.

The Omicron variant (B.1.1.529/BA.1), first detected in Botswana, harbors up to 59 mutations, of which 34 occur within the spike protein^13^. These mutations are associated with escape from natural and vaccine-induced neutralizing antibodies^14,15^ and increased affinity to angiotensin-converting enzyme 2 (ACE2), thereby increasing infectivity^16^. The mutations have been shown to reduce the effectiveness of vaccines that were developed against the Wuhan strain resulting in breakthrough infections and hospitalizations^17–19^. Based on the WHO Technical Advisory Group on COVID-19 Vaccine Composition (TAG-CO-VAC) guidelines, Moderna^20^ and Pfizer^21^ updated their vaccines to a bivalent booster containing mRNAs targeting the Wuhan as well as the Omicron variant to provide broader protection to vaccine recipients. However, the benefit of adding the Wuhan-specific mRNA was limited due to immune imprinting (antigenic sin)^22^. People who were immunized with the bivalent vaccines were already primed to respond to Wuhan and generated antibodies against Wuhan even when boosted with the Omicron-specific vaccine. Subsequently, TAG-CO-VAC upgraded its recommendations to develop a monovalent booster vaccine for COVID-19 (ref. ^23^). In line with this guideline, GEMCOVAC-OM is a monovalent booster vaccine against the BA.1 variant.

Neutralizing antibody response is considered to be a surrogate for efficacy against severe disease^24^ and has been used in immunobridging studies, especially for variant-updated COVID-19 vaccines^20^. Live virus neutralization assays are considered to be the gold standard for assessing immune responses^25^. ChAdOx1 nCoV-19, the comparator vaccine in phase 3, was designed against the Wuhan strain of SARS-CoV-2 and is expected to have lower immunogenic responses against Omicron than the Omicron-specific (BA.1) vaccine GEMCOVAC-OM. Consequently, GEMCOVAC-OM showed a 1.76-fold increase in neutralizing antibody titers against Omicron BA.1, while no significant change in the neutralization titers was observed with ChAdOx1 nCoV-19, when administered as a booster. At day 29, the lower bound 95% CI of LSGMR and the lower bound of 95% CI of seroconversion difference between GEMCOVAC-OM and ChAdOx1 nCoV-19 was above the predefined margins of noninferiority (>0.67 and >−10%, respectively) as well as superiority (>1 and >0%, respectively)^26^. Neutralizing antibody titers with GEMCOVAC-OM were 1.58 times that of ChAdOx1 nCoV-19 at day 29. These results are similar to the neutralizing antibody titer ratios observed with the other approved BA.1-adapted mRNA vaccines when compared with their prototype vaccines. At 28 days after booster vaccination, neutralizing antibody titers with Moderna’s mRNA-1 273.214 (25 μg each of ancestral Wuhan-Hu-1 and Omicron B.1.1.529 (BA.1) spike mRNAs) were 1.75 times those elicited with mRNA.1273 (ref. ^20^). Similarly, at 1 month after booster vaccination, neutralizing antibody titers with Pfizer/BioNTech’s 30 μg (15 μg each of BNT162b2 and BA.1) were 1.56 times those induced by BNT162b2 (ref. ^21^).

Furthermore, at day 90, the neutralizing antibodies further dropped in the ChAdOx1 nCoV-19 group while the titers in the GEMCOVAC-OM group returned to levels close to the baseline (GMFR 1.21). Neutralizing antibody titers with GEMCOVAC-OM were significantly higher than with ChAdOx1 nCoV-19 at day 90 (LSGMR 2.09). It is important to note that the baseline neutralizing titers in this study were already high and could potentially impact the boosting ability of the vaccines. Nonetheless, GEMCOVAC-OM elicits higher neutralization of BA.1 for at least 3 months compared with ChAdOx1 nCoV-19.

In phase 2 and phase 3, anti-spike IgG antibodies measured using enzyme-linked immunosorbent assay (ELISA) against the BA.1 variant of SARS-CoV-2 were significantly higher with GEMCOVAC-OM compared with GEMCOVAC-19 and ChAdOx1 nCoV-19 at day 29 and day 90. This difference can be attributed to the fact that GEMCOVAC-19 and ChAdOx1 nCoV-19 are designed against the D614G and Wuhan variants while GEMCOVAC-OM is designed against BA.1.

Percentage neutralization, when assessed with a surrogate virus neutralization assay (cPass) in phase 3, showed no difference in GEMCOVAC-OM and ChAdOx1 nCoV-19 arms at day 29. In both arms, the percentage neutralization was >90%, which is representative of high neutralization activity. These results are in contrast with the neutralization assessed using PRNT_50_ assay and may be due to differences in the assays. cPass is a semi-quantitative assay and has an upper limit cutoff of 100%. Differences in the vaccines beyond the cutoff cannot be observed. Importantly, live virus neutralization is considered to be the gold standard of immunogenicity assessment and a correlate of protection.

Omicron variants, although more infective, are associated with lower rates of hospitalization and death compared with previous SARS-CoV-2 strains. Studies have shown that the intrinsic severity of the Omicron variant is similar to that of its predecessors and cross-reactive protection from vaccination and/or infection plays an important role in reducing the severity of Omicron-associated COVID-19 (ref. ^27^). A study published by Naranbhai et al.^28^ showed that T cell cross-reactivity to the SARS-CoV-2 Omicron variant remained intact in most of the previously infected and vaccinated individuals. However, a subset of individuals (approximately 21%) exhibited a more than 50% decrease in T cell reactivity to the Omicron spike^28^. We observed similar findings in which GEMCOVAC-19 and ChAdOx1 nCoV-19 showed cross-reactive cellular responses against Omicron. However, the cellular responses with the Omicron-specific GEMCOVAC-OM were higher. In phase 2, GEMCOVAC-19 exhibited cross-reactive IFNγ^+^ T cells against the BA.1 variant. However, at day 90, the TNF^+^CD4^+^ and IL-2^+^CD8^+^ T cell responses were higher in GEMCOVAC-OM compared with GEMCOVAC-19. Similarly, in phase 3, GEMCOVAC-OM showed an increase in IFNγ^+^CD8^+^ T cells from baseline to day 29 and a significantly higher expression of TNF^+^CD4^+^, IL-2^+^CD4^+^ and IL-2^+^CD8^+^ T cells at day 29 compared with ChAdOx1 nCoV-19. Recent findings have shown that vaccine-induced CD8^+^ T cell responses are important for long-term immunity and should also be considered as a correlate of protection^29,30^. Both IFNγ and TNF play crucial roles in controlling intracellular pathogen infections. It is worth noting that IFNγ co-operates with other T_H_1 cell cytokines and acts synergistically to enhance their ability to eliminate pathogens^31,32^. IL-2 serves various functions in the development of effector and memory CD8^+^ T cell responses. Kahan et al.^33^ have demonstrated that a specific subset of CD8^+^ T cells, capable of intrinsic IL-2 expression, exhibit stem-like characteristics, display a memory phenotype, can withstand exhaustion and effectively regulate chronic viral infections^33^. Additionally, at day 90, GEMCOVAC-OM showed significantly higher TNF^+^CD4^+^ T cells and IL-2^+^CD8^+^ T cells compared with GEMCOVAC-19 (phase 2) and higher TNF^+^CD4^+^, IL-2^+^CD4^+^ and IL-2^+^CD8^+^ T cells compared with ChAdOx1 nCoV-19 (phase 3), indicating a durable T cell protection with BA.1-specific vaccine.

In addition to T cell responses, B cell responses play an important role in generating durable immunity. T_H_1 and T_H_2 cell responses support B cell activation and differentiation for antigen-specific antibody production and long-term memory development through germinal cell responses^34,35^. In our study, we observed a significant increase in the Omicron-specific B cell population at day 29 following vaccination. This response was not observed with ChAdOx1 nCoV-19.

In adults, women have shown to mount higher immune responses than men and are more likely to report adverse reactions^36^. We conducted a sex-disaggregated analysis on the humoral immunogenicity and safety data from phase 3. There were no significant differences in the neutralizing antibodies and anti-spike IgG titers between men and women who received ChAdOx1 nCoV-19 at day 29 and day 90. Additionally, no difference in AEs was observed. These findings contrast with sex-disaggregated data published by Marchevsky et al.^37^ where a small, statistically significant difference was found in the anti-spike IgG titers, with higher titers in female participants and there were twice as many systemic reactions reported by women. However, it is important to note that there were many differences in the study design and the populations (sample size, primary versus booster vaccination, and ethnicity) assessed in these two studies that can have a substantial impact on outcomes. There were no significant differences in the neutralizing and anti-spike IgG titers between men and women receiving GEMCOVAC-OM, except at day 29, where neutralizing titers were significantly higher in women. Interestingly, female participants in the GEMCOVAC-OM group reported significantly higher local and systemic AEs. These findings are in line with the real-world evidence on COVID-19 mRNA vaccines (BNT162b2, mRNA-1273) which describe higher reactogenicity in females compared with males^38,39^.

The study has several limitations. (1) The study participants were predominantly male, and as such, the results may be more representatives of men than women. We have conducted a sex-disaggregated analysis; however, the analysis was not powered. (2) Ideally, GEMCOVAC-OM should have been assessed in a clinical trial using another Omicron-specific mRNA vaccine as a comparator. However, the mRNA vaccines from Moderna and Pfizer were not approved or available in India due to which ChAdOx1 nCoV-19, which was designed against the ancestral spike (Wuhan), was used as a comparator. This makes it difficult to accurately compare and characterize GEMCOVAC-OM. (3) Blinding of participants in the study was not possible due to the different mechanisms of delivery, which has the potential to introduce a bias in the safety assessment. (4) Given the multiple COVID-19 waves and asymptomatic infections, accurate data on past COVID-19 infections could not be obtained. This makes analysis of the impact of previous infection on the safety and immunogenicity of the vaccine difficult. (5) At the time of the study, booster vaccines were approved in India and, therefore, using a placebo as an arm in the clinical trial would have been unethical. An efficacy study was not possible and, hence, an immunobridging approach was used.

The platform used to develop GEMCOVAC-OM has certain advantages over the other mRNA vaccines approved for COVID-19. GEMCOVAC-OM is administered intradermally using a needle-free injection system called Tropis. The dermis has a rich network of dendritic cells, macrophages and T cells^40^. Vaccination into the dermis provides a more potent and broader immunogenic response than vaccinating into the muscle^24^. Additionally, the absence of a needle obviates challenges of need for sharps disposal, needle-stick injuries, cross-contamination and needle phobia. These advantages have translated into the field where vaccinators and caregivers expressed a preference for Tropis due to the ease of use, appearance, response to vaccination and increased coverage^41^. The device has been well characterized and approved by numerous regulatory bodies including the World Health Organization. Each Tropis device can inject up to 20,000 vaccines, and the additional cost of the device is negligible and outweighed by advantages. Thermostability is another critical factor in ensuring equitable access to effective mRNA vaccines in LMICs^42^. The degradation of mRNA in the presence of water, attributed to oxidation and hydrolysis^43^, required mRNA vaccines to be stored and transported at subzero temperatures. This makes the vaccine inaccessible to LMICs due to lack of infrastructure and the cost of establishing subzero cold chains. GEMCOVAC-OM is a lyophilized vaccine and can be transported and stored at 2–8 °C for 12 months, facilitating democratization of access to vaccine. Lastly, the samRNA platform used for GEMCOVAC-OM offers a promising avenue for effective and safe immunization against infectious diseases. samRNA vaccines can induce potent immune responses at lower doses due to their ability to amplify antigen production within the body, potentially reducing the risk of adverse reactions^6,44^.

In summary, these results show that GEMCOVAC-OM is safe and generates immunogenic responses when administered as a booster. This self-amplifying, thermostable mRNA platform delivered intradermally provides a framework for next-generation vaccines that can improve accessibility and global equity.

## Methods

### Study design

This was a prospective, multicenter, open-label, randomized phase 2 seamlessly followed by a phase 3 study to evaluate the safety, tolerability and immunogenicity of GEMCOVAC-OM as a booster in participants 18 years of age and older. In this seamless study, phase 2 safety data till day 7 were analyzed and presented to an independent DSMB. The DSMB evaluated these data and provided their approval to initiate the phase 3 part of the study. The phase 3 study was conducted at 20 hospitals in 13 cities across India in compliance with the principles defined in the Declaration of Helsinki, International Conference for Harmonisation Good Clinical Practice Guideline. The study protocol was approved by the local ethics committee at each study site and Central Drugs Standard Control Organisation, the central licensing authority in India. This clinical trial is registered with the Clinical Trial Registry India, CTRI/2022/10/046475. Details on the sites and Ethics Committees can be found in Supplementary Information.

An interim analysis was planned at day 29 of phase 3 where the immunogenicity and safety of the participants was assessed and presented to the Central Drugs Standard Control Organisation for Emergency Use Authorization.

### Participants

In phase 2, the safety and immunogenicity of GEMCOVAC-OM as a booster was compared with the prototype vaccine GEMCOVAC-19 designed against the spike protein of the D614G strain of SARS-CoV-2 (*n* = 140). Participants were randomized to receive the vaccines in a 1:1 ratio.

The phase 3 study comprised a safety and an immunogenicity cohort. The safety cohort consisted of 3,140 participants of whom 3,000 were enrolled into the GEMCOVAC-OM arm and 140 were enrolled into the ChAdOx1 nCoV-19 arm. Within the safety cohort, the immunogenicity cohort consisted of 420 participants of whom 280 were enrolled into the GEMCOVAC-OM arm and 140 were enrolled into the ChAdOx1 nCoV-19 arm. Participants were healthy adults (male or female participants reported by self), 18 years of age or older, who have received two doses of either BBV152 or ChAdOx1 nCoV-19, 4 months before the screening visit. Additionally, the participants should have had no known COVID-19 infection at least 3 months before the screening visit. Key exclusion criteria included pregnant or lactating mothers, individuals with illnesses that in the opinion of the investigator may affect safety, and the immunocompromised. Detailed inclusion and exclusion criteria are provided in the protocol (Supplementary Information). Participants were screened on the basis of medical history, vital signs and physical examination before enrollment. Eligible participants provided signed informed consent forms at enrollment. Participants were compensated at every visit for their time and cost of travel.

### Randomization and masking

Participants who met the inclusion criteria and successfully completed all screening procedures were randomized in the study by using the interactive web response system (IWRS). Unique randomization codes were assigned to the participants and remained unchanged until the completion of the trial. The randomization codes were generated through Proc Plan using SAS version 9.4 or higher (SAS Institute) by an independent biostatistician. The final randomization list was filed securely by the independent biostatistician and accessible to authorized persons only. Participants were enrolled by investigators with the help of the IWRS.

In phase 3, consecutive 420 in the immunogenicity cohort were randomized in a 2:1 ratio into GEMCOVAC-OM and ChAdOx1 nCoV-19 by stratified block randomization through the IWRS. A randomization code was assigned to each participant in sequence in the order of enrollment, and then the participants received the investigational products labeled with the same code. This was an open-label study, and no masking was performed.

### Procedures

GEMCOVAC-OM consists of an in vitro transcribed mRNA encoding for the spike protein of the Omicron variant of the SARS-CoV-2 virus and cationic lipid nano-emulsion in a buffer containing 10% sucrose in 10 mM sodium citrate, pH 6.5 (ref. ^45^). The complete antigenic sequence that was used has been published in the DDBJ database (accession no. LC769018). GEMCOVAC-OM, 10 µg in 0.1 ml, was administered intradermally using a Tropis needle-free injection system (PharmaJet). More information on the vaccine, mRNA platform and development can be found in Supplementary Information.

ChAdOx1 nCoV-19 (COVISHIELD), the comparator vaccine in phase 3, consisted of Corona Virus Vaccine (Recombinant) 5 × 10^10^ viral particles. This vaccine is based on recombinant, replication-deficient chimpanzee adenovirus vector encoding the SARS-CoV-2 spike glycoprotein, produced in genetically modified human embryonic kidney 293 cells. ChAdOx1 nCoV-19 was administered intramuscularly.

GEMCOVAC-19 consists of an in vitro transcribed mRNA encoding for the spike protein of the D614G variant of the SARS-CoV-2 virus and cationic lipid nano-emulsion in a buffer containing 10% sucrose in 10 mM sodium citrate, pH 6.5. The complete antigenic sequence that was used has been published in the DDBJ database (accession no. LC776732.1). GEMCOVAC-19, 10 µg in 0.5 ml, was administered intramuscularly.

The participants were screened on visit 1 (day 1), which included a validated reverse transcription polymerase chain reaction (RT–PCR) for SARS-CoV-2. Regardless of the outcome of the RT–PCR, the participants who fit the inclusion criteria were enrolled and the vaccine was administered on the same day. Those found to be RT–PCR positive would be excluded from the immunogenicity analysis to avoid confounding. Importantly, during the trial, in India, a third dose of BBV152 or ChAdOx1 nCoV-19 (precautionary dose) was approved for participants who had received primary doses of BBV152 or ChAdOx1 nCoV-19, respectively. However, individuals who had taken two doses of BBV152 as their primary vaccination were not eligible for a third dose of ChAdOx1 nCoV-19. Keeping in line with these vaccination guidelines, participants with ChAdOx1 nCoV-19 as their primary vaccination were randomized to get ChAdOx1 nCoV-19 or GEMCOVAC-OM, whereas participants with BBV152 as their primary vaccination received GEMCOVAC-OM only in the clinical trial.

Participants were provided an e-diary or a paper diary to record the solicited AEs till day 7 and unsolicited AEs as well as concomitant medication taken, if any, till the end of the study. A telephone call was placed to all the participants at day 7 to record any additional AEs, if any. Participants visited the study site for visit 2 (day 29 + 7), visit 3 (day 90 + 14) and visit 4 (day 180 + 14). Blood for assessing immunogenicity was drawn at visit 1 before vaccination (baseline), day 29 and day 90. Safety was assessed throughout the duration of the study.

### Endpoints

In phase 2, the primary endpoints were to compare the safety and anti-spike IgG antibodies between the two vaccinated arms at day 29. Secondary endpoints included comparison of seroconversion as assessed by ≥2-fold rise in anti-spike IgG antibody titers from baseline, percentage neutralization by a surrogate neutralization (cPass) assay and cellular immune responses at day 29. Exploratory endpoints included comparison of anti-spike IgG antibodies, percentage neutralization by cPass assay and cellular immune responses at day 90.

In phase 3, the primary endpoint was the demonstration of noninferiority of neutralizing antibody GMT assessed by a plaque reduction neutralization test (PRNT_50_) assay in terms of LSGMR at day 29 and difference in seroconversion (≥2-fold rise in antibody titers at day 29 from baseline) between GEMCOVAC-OM and ChAdOx1 nCoV-19. Secondary endpoints included comparison of safety, LSGMR and seroconversion in terms of anti-spike IgG antibody titers, percentage neutralization by a surrogate virus neutralization assay (cPass assay, GenScript) and cell-mediated immunity assessment by intracellular cytokine expression at day 29. Exploratory endpoints included humoral and cellular immune response assessment at day 90.

### Immunogenicity assessment

Although the trial was open-label, laboratory analysis was conducted in a blinded manner. Measurements were taken from distinct samples. Information on the materials used is provided in detail in Supplementary Information.

Neutralizing antibody titers were assessed by the PRNT_50_ assay at the Interactive Research School for Health Affairs (IRSHA, Bharati Vidyapeeth, Deemed to be University, Pune) that was previously developed^46^ and then optimized for the BA.1 Omicron variant of SARS-CoV-2 (SARS-CoV-2-IND/0005/2022; B.1.1.529.1 lineage). In brief, Vero E6 cells were initially seeded at a density of 1 × 10^5^ cells ml^−1^ in 24-well plates using Minimum Essential Medium (MEM) containing 10% fetal bovine serum (FBS) and antibiotics and allowed to incubate overnight at 37 °C with 5% CO_2_. Serum samples, initially diluted at 1:5 ratios, were subjected to heat inactivation for 30 min at 56 °C. Subsequently, a fourfold serial dilution was executed, and these serum dilutions were mixed in equal proportions with the SARS-CoV-2 virus with a titer ranging from 600 to 1,000 plaque-forming units (pfu) per milliliter. The serum–virus mix was then incubated for 1 h at 37 °C within a humidified incubator with 5% CO_2_. Following incubation, 100 μl of the resultant mixture was introduced into duplicate wells of the seeded 24-well plate and subjected to an additional 3-h incubation at 37 °C in a humidified incubator with 5% CO_2_. Then, 1 ml of an overlay medium, constituting MEM, Aquacide-II, 2% FBS and antibiotics, was added to the Vero cell monolayer. Plates were then incubated for 6 days at 37 °C within a humidified incubator with 5% CO_2_. At the end of this incubation period, the overlay medium was removed, and cells were fixed through the application of 3.7% formaldehyde. After washing with phosphate-buffered saline, cells were stained using 1% crystal violet. Plates were washed once more and air-dried. Viral plaques were counted using the C.T.L. ImmunoSpot platform. PRNT_50_ titers were determined using standard logistic regression model. Neutralization was also assessed using a semi-quantitative surrogate virus neutralization assay (cPass, GenScript)^47^ for the BA.1 variant.

Anti-IgG responses against the spike glycoprotein of B.1.1.529 Omicron variant of SARS-CoV-2 was assessed by an in-house developed indirect ELISA. In brief, 96-well ELISA plates (Nunc Maxisorp) coated with spike protein (full length from Sino Biologicals, 40589-V08H26) were washed thrice with phosphate-buffered saline-Tween (PBS-T). Plates were blocked with 3% nonfat dried milk. Diluted sera samples were added to the blocked plates and incubated at room temperature for 2 h. Plates were then washed thrice with PBS-T and incubated at room temperature with detection antibody (1:5,000), anti-human IgG (Fc region specific from Sigma A0170) for 1 h at room temperature. After secondary antibody incubation, plates were washed thrice with PBS-T before addition of TMB substrate. Color development was quenched with 3 M HCl after 20 min of incubation at room temperature. Plates were read at 450 nm using a plate reader. The assay background was calculated from the 10 s.d. added to the average of the readouts where there was no sample but diluent in the wells. For all samples, IgG titers were an interpolation of previously calculated assay background in 5 parameter logistic fit of sample dilution versus absorbance.

**Extended Data Fig. 3:**
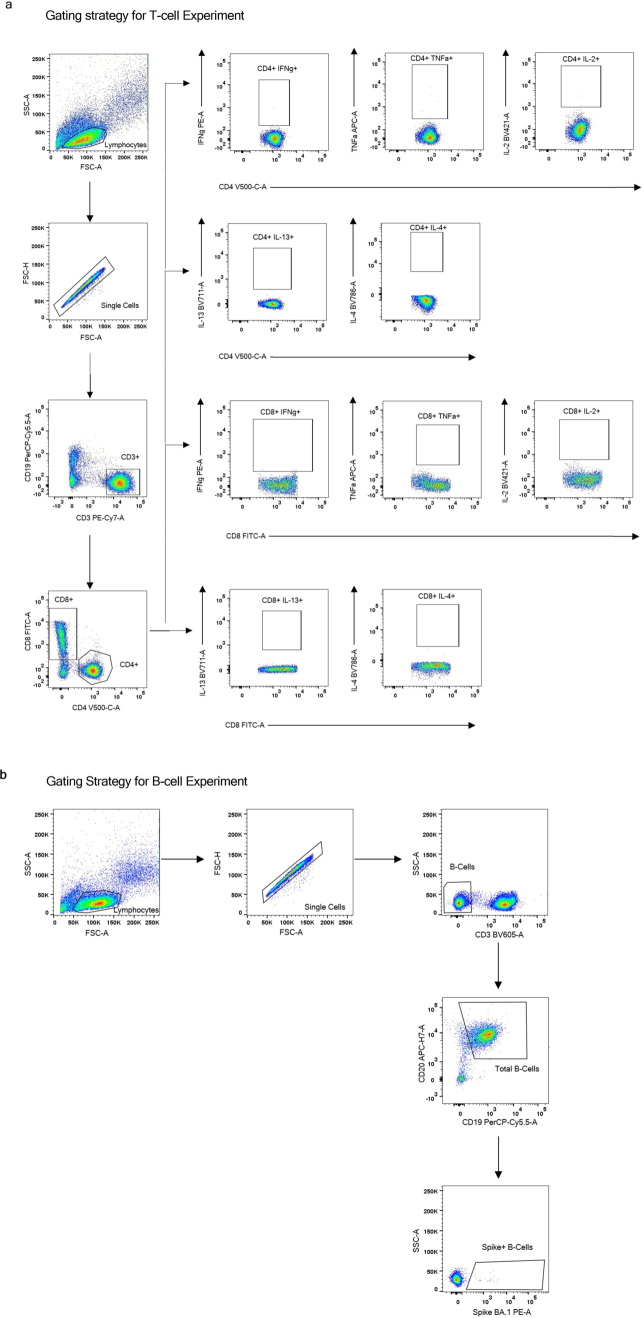
Gating strategy used to measure Omicron B.1.1.529. (**A**) Spike-specific effector T-cell responses and (**B**) B-cell population.

PBMCs were isolated using BD Vacutainer CPT with sodium citrate tubes following the manufacturer’s guidelines and subsequently cryopreserved in liquid nitrogen. For immune-phenotyping purposes, frozen PBMCs were thawed and allowed to rest in complete RPMI 1640 culture medium (CRPMI) supplemented with 10% FBS, 100 U ml^−1^ penicillin and 0.1 mg ml^−1^ streptomycin (1× pen-strep) for 18–22 h. Gating strategies for both T cell and B cell experiments are given in Extended Data Fig. 3. In T cell response analysis, intracellular cytokine staining (ICS) was performed using 0.5 million PBMCs in 100 µl CRPMI medium per well in a V-bottom plate. These cells were stimulated with a 1 µg ml^−1^ epitope mapping 15-mer peptide pool derived from the Omicron B.1.1.529/BA.1 spike glycoprotein peptides, specifically the PepTivator SARS-CoV-2 Prot S B.1.1.529/BA.1 Mutation Pool. Stimulation was carried out in the presence of 1 µg ml^−1^ BD FastImmune (anti-CD28/49d antibody)^48^ for 6 h, with the addition of 1 µl of Brefeldin-A during the final 4 h of stimulation. After stimulation, PBMCs were washed and subjected to surface and ICS staining using antibodies targeting CD3 PE-Cy7 (BD 557851, clone SK7, 1:20), CD4 BV480 (BD 566104, clone SK3, 1:20), CD8 FITC (BD 555366, clone RPA-T8, 1:5), IFNγ PE (BD 559327, clone B27, 1:5), TNF APC (BD 551384, clone MAb11, 1:5), IL-2 BV421 (BD 562914, clone 5344.111, 1:20), IL-2 BV786 (BD 564113, clone MP4-25D2, 1:10), IL-13 BV711 (BD 564288, clone JES10-5A2, 1:10) and CD19 PerCP-Cy5.5 (BD 561295, clone HIB19, 1:20) markers. ICS was executed utilizing the BD cytofix/cytoperm kit following the manufacturer’s instructions. Antibody incubation was carried out for 30 min at 4 °C. Phorbol myristate acetate (PMA)–ionomycin was used as positive control. To assess the B cell population specific to the B.1.1.529 spike protein, PBMCs were initially labeled with biotinylated spike protein specific to Omicron B.1.1.529. Subsequently, surface staining was performed with common surface markers CD3 BV605 (BD 563219, clone SK7, 1:20), CD19 PerCP-Cy5.5 (BD 561295, clone HIB19, 1:20), CD20 APC-H7 (BD 560734, clone 2H7, 1:20). Following staining and washing steps, PBMCs were resuspended in fluorescence-activated cell sorting buffer, acquired using the FACSLyric system (BD Biosciences), and analyzed using FlowJo software version 10.8.1 (FlowJo LLC, BD Biosciences).

### Safety assessment

Solicited events data were captured up to 7 days after booster vaccine administration through an electronic or paper diary. Local solicited events included pain, redness, swelling, warmth, pruritus and bruising. Systemic solicited events included fever, headache, myalgia, arthralgia, fatigue, malaise, nausea and chills. Unsolicited events were assessed throughout the duration of the study. AE terms were coded using Medical Dictionary for Regulatory Activities. These AEs were graded on the basis of the Division of AIDS criteria^49^. Myocarditis was considered as an AE of special interest; site investigators were asked to thoroughly evaluate participants with any symptoms of chest pain, breathlessness or palpitations.

### Statistical analysis

No formal sample size calculations were performed for phase 2. Phase 3 consisted of a safety and an immunogenicity cohort. The safety cohort consisted of 3,140 participants of whom 3,000 were included in the GEMCOVAC-OM arm. The immunogenicity cohort was analyzed for two primary endpoints based on World Health Organization guidelines^50^, with individuals randomized to GEMCOVAC-OM and ChAdOx1 nCoV-19 in a 2:1 ratio. A sample size of 420 (280 in GEMCOVAC-OM and 140 in ChAdOx1 nCoV-19) was found adequate for assessing noninferiority of neutralizing antibody titers if the lower limit of the two-sided 95% CI of the LSGMR (GMT_GEMCOVAC-OM_/GMT_ChAdOx1 nCoV-19_) was >0.67 considering a standard deviation of 1.82, alpha error of 5%, power of 90% and dropout rate of 20%. A sample size of 381 (254 in GEMCOVAC-OM and 127 in ChAdOx1 nCoV-19) was found adequate for assessing the noninferiority of seroconversion difference considering a margin of −10%, alpha error of 5%, power of 90% and dropout rate of 20%. The sample size of 420 (280 in GEMCOVAC-OM and 140 in ChAdOx1 nCoV-19 arm) was considered in this study to provide adequate numbers for the statistical analysis of both the primary endpoints.

All immunogenicity analysis was performed in the full analysis set (intention to treat) population. The observed GMT and associated 95% CIs (Clopper–Pearson method) were calculated on the basis of log-transformed antibody titers. The rise in neutralizing antibody titers from baseline to day 29 was compared using a paired *t*-test. The LSGMR of neutralizing antibody titers in both the arms at day 29 from the PRNT_50_ (BA.1 strain of SARS-CoV-2) assay was calculated using ANCOVA with baseline titers as covariates. If the lower limit of the two-sided 95% CI of the LSGMR was >0.67, GEMCOVAC-OM would be considered noninferior to ChAdOx1 nCoV-19. Seroconversion in terms of neutralizing antibody titers from PRNT_50_ assay was defined as a ≥2-fold rise in titers at day 29 from baseline. The difference in seroconversion was determined using the Meitinen–Nurminen method. If the lower bound of the two-sided 95% CI for seroconversion difference was >−10%, GEMCOVAC-OM would be considered noninferior to ChAdOx1 nCoV-19.

Similarly, for the secondary endpoint, LSGMR of anti-Spike IgG antibody titers from ELISA at day 29 was assessed using ANCOVA, with baseline titers as covariates. The 95% CI was calculated for percentage by using the Clopper–Pearson method. The difference in seroconversion at day 29 for anti-spike IgG antibodies from ELISA were calculated using the Meitinen–Nurminen method. The difference in the change in mean percentage neutralization from baseline to day 29, assessed by the cPass assay, was analyzed using ANCOVA with baseline neutralization considered as a covariate. In cell-mediated immunity, change in expression from baseline to day 29 was assessed by either a two-sided paired *t*-test or Wilcoxon signed-rank test depending on normality of the data. Expression at day 29 was compared using a two-sided *t*-test or Wilcoxon rank sum base based on normality. The normality of the data was assessed using a Shapiro–Wilk test. This was also performed for subgroups based on their primary vaccination of either BBV152 or ChAdOx1 nCoV-19.

Sex-disaggregated analysis was conducted for humoral and safety data from phase 3. For humoral immunogenicity, ANCOVA was used to compare the differences in neutralizing antibody and anti-spike IgG between men and women at days 29 and 90, using baseline titers as covariates. For safety, unadjusted OR was calculated along with the 95% CI.

Data were collected using Clinion (version 3.1). Statistical analysis for humoral immunogenicity was performed using Statistical software SAS version 9.4 (SAS Institute). Figures were generated using GraphPad Prism (Version 9.5.1). The Spearman rank correlation coefficient (denoted as ‘*r*’) was computed for all pairs of parameters utilizing the corrplot package (version 0.92) within RStudio (version 2022.12.0.0). To complement the correlogram, two-tailed *P* values associated with Spearman rank correlations were calculated through the corr.mtest function and visualized using the corrplot function.

### Reporting summary

Further information on research design is available in the Nature Portfolio Reporting Summary linked to this article.

## Online content

Any methods, additional references, Nature Portfolio reporting summaries, source data, extended data, supplementary information, acknowledgements, peer review information; details of author contributions and competing interests; and statements of data and code availability are available at 10.1038/s41591-024-02955-2.

### Supplementary information

Supplementary Information Additional phase 2 and phase 3 data; antibodies, reagents, cells and viruses used for analysis; samRNA platform; methods used for characterization of GEMCOVAC-OM.

Reporting Summary

Supplementary Data 1 Phase 2 and 3 clinical trial protocol.

Supplementary Data 2 Statistical analysis plan for the clinical study.

## Figures and Tables

**Extended Data Table 1:**
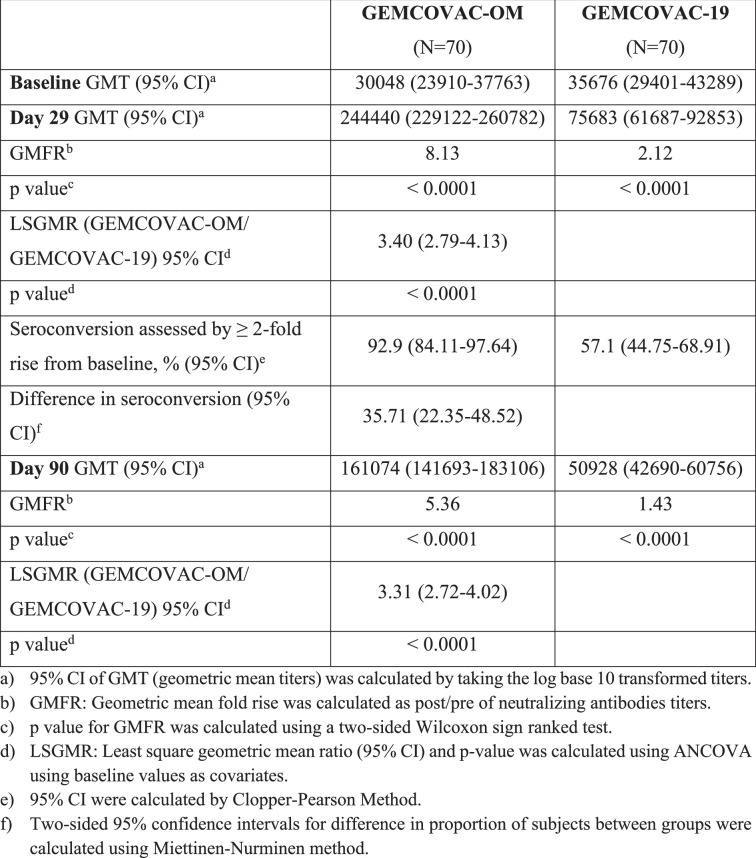
Anti-spike IgG antibodies in phase 2

## Data Availability

Individual participant data will be made available for meta-analysis when the trial is complete. The request should be approved by an ethics committee or the institutional review board of the institution to which the person requesting the information belongs. If approved, it should be directed to the corresponding author at sanjay.singh@gennova.co.in. The requester will need to sign a data access agreement. Data will be shared through a secure online platform within 2 months from the signing of the access agreement. The aggregated data are included in this manuscript. DDBJ datasets were used to design GEMCOVAC-19 (accession no. LC776732.1) and GEMCOVAC-OM (accession no. LC769018).

## Code availability

No custom code or mathematical algorithms were used in this study.
