# Supplementary material for: An Omicron-specific, self-amplifying mRNA booster vaccine for COVID-19: a phase 2/3 randomized trial
Source: Nat Med. 2024 Apr 18;30(5):1363–72. doi: 10.1038/s41591-024-02955-2 (PMC11108772; doi:10.1038/s41591-024-02955-2)
Supplement: Supplementary file 1 — Additional phase 2 and phase 3 data; antibodies, reagents, cells and viruses used for analysis; samRNA platform; methods used for characterization of GEMCOVAC-OM. [file 41591_2024_2955_MOESM1_ESM.pdf]

nature medicine

Article https://doi.org/10.1038/s41591-024-02955-2

An Omicron-specific, self-amplifying mRNA booster vaccine for COVID-19: a phase 2/3 randomized trial

In the format provided by the authors and unedited

Supplementary Information: An omicron-specific, self-amplifying mRNA booster vaccine for COVID-19: a phase 2/3 randomized trial

Table of Contents

1. **Additional phase 2 and phase 3 data 5**

2. **Antibodies, reagents, cells, and virus used for analysis. 30**

3. **Self-Amplifying mRNA Vaccine Platform for GEMCOVAC-OM 32**

4. **Methods used for the characterization of the GEMCOVAC-OM. 36**

Supplementary Tables

Supplementary Figure 1. Anti-spike IgG antibodies (phase 2).. 11

Supplementary Figure 2. Lymphocyte population (phase 2)... 13

Supplementary Figure 3. Th1 cytokine producing CD4$^+$ T-cells (phase 2).................................... 14

Supplementary Figure 4. Th1 cytokine producing CD8$^+$ T-cells (phase 2).................................... 15

Supplementary Figure 5. Th2 cytokine producing CD4$^+$ and CD8$^+$ T cells (phase 2) 16

Supplementary Figure 6. Live Virus Neutralization by PRNT (phase 3)....................................... 21

Supplementary Figure 7. Anti-spike IgG antibodies (phase 3).. 22

Supplementary Figure 8. Adverse events (phase 3) 23

Supplementary Figure 9. Neutralizing antibodies disaggregated by sex (phase 3). 26

Supplementary Figure 10. Anti-spike IgG antibodies disaggregated by sex (phase 3). 28

Supplementary Figure 11. Schematic of mRNA-628.2... 32

Supplementary Figure 12. Manufacturing process of GEMCOVAC-OM 39

Supplementary Tables

Supplementary Table 1. Clinical trial contributors..5

Supplementary Table 2. LSGMR and seroconversion of anti-spike IgG antibodies (phase 2) 10

Supplementary Table 3. Percent neutralization by cPASS assay (phase 2)..................................12

Supplementary Table 4. Summary of adverse events (phase 2) 17

Supplementary Table 5. LSGMR and seroconversion of neutralizing antibodies (phase 3).........18

Supplementary Table 6. LSGMR and seroconversion of anti-spike IgG antibodies (phase 3) 19

Supplementary Table 7. Percent neutralization by cPass assay (phase 3).....................................20

Supplementary Table 8. Demography disaggregated by sex (phase 3)..24

Supplementary Table 9. Neutralizing antibodies disaggregated by sex (phase 3) 25

Supplementary Table 10. Anti-spike IgG antibodies disaggregated by sex (phase 3) 27

Supplementary Table 11. Sex-disaggregated adverse events (phase 3)..29

Supplementary Table 12. List of antibodies, reagents, cells, and virus used for analysis. 30

Supplementary Table 13. Oligonucleotides designed to detect the (-) strand of mRNA. 35

Supplementary Table 14. qPCR data of mRNA transfected cells. 35

Supplementary Table 15. mRNA drug substance characterization 36

Supplementary Table 16. mRNA drug product characterization. 37

1. Additional phase 2 and phase 3 data

Supplementary Table 1. Clinical trial contributors

**Hospital name, City, State**	**Ethics Committee Number**	**Principal Investigator**	**Co-Investigators**	**Study Coordinators/Field worker/Nurse**
Ace Hospital & Research Centre Pune, Maharashtra	ECR/474/Inst/MH/2013/RR-19	Dr. Himanshu Pophale, MBBS, M.D	1. Dr. Rajiv Adkar<br>2. Dr. Bharat Jain<br>3. Dr. Aditya Bari	1. Ms. Sunita Banger<br>2. Ms. Sunita Mehra<br>3. Ms. Savita Kokre<br>4. Mr. Mohan Ghodke<br>5. Dr. Neha Majgaonkar<br>6. Ms. Pallavi Bankhele<br>7. Ms. Sayali Chavan<br>8. Ms. Sushmita Sarnikar<br>9. Ms. Mohini Upadhyay<br>10. Ms. Prerna<br>11. Ms. Pranjali Ghuguskar
Lokamanya Medical Research Centre, Pune, Maharashtra	ECR/175/Inst/MH/2013/RR-19	Dr. Prakash Shende, MBBS, M.D., CCD	1. Dr. Rakesh Waghamre<br>2. Dr. Rahul Doshi	1. Ms. Sonali Shinde<br>2. Ms. Ambika Ghodmare<br>3. Ms. Pragati Babhulkar<br>4. Ms. Kajal Sarkar<br>5. Mr. Nikhil Bagmare<br>6. Ms. Usha Shinde<br>7. Ms. Anagha Bhagat<br>8. Ms. Asmita Bikkad<br>9. Ms. Geetanjali Rathod<br>10. Ms. Chaitali Shinde
CIMETs Inamdar Multispeciality Hospital, Pune, Maharashtra	ECR/354/Inst/MH/2013/RR-20	Dr. Ravindra Baban Shinde, MBBS, M.D.	1. Dr. Kailash Chandra Joshi<br>2. Dr. Anjali Rajan Pillay<br>3. Dr. Arshiya Raheman	1. Ms. Komal Sabale<br>2. Ms. Yogita Khairnar<br>3. Mrs. Tessy D' Cruz<br>4. Mrs. Rohini Munswami<br>5. Mr. Sachin Patil<br>6. Mrs. Fouzia Deshmukh<br>7. Ms. Rutuja Karve
Dr. D. Y. Patil Medical College Hospital & Research Centre, Pune, Maharashtra	ECR/361/Inst/MH/2013/RR-19	Dr. Vikram Vikhe, MBBS, MD	1. Dr. Shrikant Tripathy<br>2. Dr. Arjun Lal Kakarani<br>3. Dr. Shubhangi Kanitkar<br>4. Dr. Shahzad Mirza<br>5. Dr. Prachi Athavale<br>6. Dr. Harshad Patel	1. Dr. Ahsan Faruqui<br>2. Dr. Avni Reddy<br>3. Dr. Akshata Borle<br>4. Ms. Savita Mahajan<br>5. Dr. Rakesh Kothavale<br>6. Ms. Mannasa Nimmala<br>7. Ms. Shambhavi Ghotankar<br>8. Ms. Ayesha Tamboli<br>9. Ms. Rutuja Ingale<br>10. Ms. Shreya Jekate<br>11. Ms. Rutuja Gudade<br>12. Ms. Priyanka Salve<br>13. Ms. Pramila Mhaske

				14. Ms. Dhanashree Kakad<br>15. Ms. Pooja Sharma<br>16. Ms. Bhagyashri Takbhate
Vedant Multispeciality Hospital, Pune, Maharashtra	ECR/1426/Inst/MH/2020	Dr. Abhishek Karmalkar, MBBS, M.D (Medicine)	1. Dr. Nilesh Patil<br>2. Dr. Amit Kumar Pande	1. Ms. Yasmeen Momin<br>2. Ms. Suchita Mokashi<br>3. Ms. Rachana Shinde<br>4. Mr. Amarsing Pawara<br>5. Mr. Kaustubh Harde<br>6. Ms. Ashiya Siddqui<br>7. Ms. Sonali Tulse<br>8. Ms. Surbhi Gokhale
Baramati Hospital, Pune, Maharashtra	ECR/1449/Inst/MH/2020	Dr. Bhaskar Vilasrao Jedhe-Deshmukh, MBBS, M.D.	1. Dr. Sunil Ramdas Pawar	1. Ms. Tanuja Shinde<br>2. Ms. Vrushali Shinde<br>3. Ms. Pooja Yadav<br>4. Ms. Prajakta Nibalkar
Dhadiwal Hospital in coalition with Shreeji Healthcare, Nashik, Maharashtra	ECR/1149/Inst/MH/2018/RR-21	Dr. Krishna Madhukar Giri, MBBS, MD	1. Dr. Prasant Patole<br>2. Dr. Ganesh Wadgaonkar<br>3. Dr. Rajesh Dhadiwal<br>4. Dr. Deepak Chirmade	1. Ms. Nivedita Kavi<br>2. Ms. Darshana Mahajan<br>3. Ms. Komal Adhangale<br>4. Ms. Vaishnavi Zode<br>5. Ms. Rutuja Bhandekar<br>6. Ms. Vishakha Pandit<br>7. Mr. Chetan Salukhe<br>8. Ms. Harshada Jadav<br>9. Ms. Mayuri Inait<br>10. Ms. Rohini Thakre<br>11. Ms. Jayshri Palve<br>12. Mr. Sandip Ahire
Ashirwad Hospital and research centre Ulhasnagar, Maharashtra	ECR/247/Inst/MH/2013/RR-19	Dr. Shrikant Vishnu Deshpande MBBS, MD	1. Dr. Shreyas Deshpande<br>2. Dr. R.M. Mundada	1. Ms. Kajal Chitte<br>2. Ms. Tejashree Patil<br>3. Ms. Karuna Deshmukh
Meditrina Institute of Medical Sciences, Nagpur, Maharashtra	ECR/608/Inst/MH/2014/RR-20	Dr. Ajay Bulle, MBBS, M.D.	1. Dr. Tapan Bodele	1. Ms. Shreya Mokati<br>2. Mr. Hemant Ghagre<br>3. Mr. Prajwal Landge<br>4. Mr. Shareef Sheikh<br>5. Ms. Awanti Rodge<br>6. Ms. Rutuja Bhandekar
All India Institute of Medical Sciences (AIIMS) Raipur Chhattisgarh	ECR/714/Inst/CT/2015/RR-21	Dr. Md. Sabah Siddiqui, MBBS, MD	1. Dr. Kambagiri Pratusha<br>2. Dr.Atul Jindal<br>3. Dr. Sanjay Samria<br>4. Dr. Abinaya Kannan	1. Mr. Ankit Vishwakarma<br>2. Mr. Lagendra Kumar Sahu

Downtown hospital ltd, Guwahati Assam.	ECR/549/Inst/AS/2014/RR-20	Dr.Swapna v Borthakur, MBBS, M.D.	1. Dr. Rupam Das<br>2. Dr. Akshay Salunke<br>3. Dr. Aman Pandey<br>4. Dr. Jithin Shaji	1. Mr. Nimit Patel<br>2. Mr. Sharit Shekar Baruha<br>3. Ms. Swapna Das
Induss Hospital, Hyderabad Telangana	ECR/1606/Inst/TG/2021	Dr. V. Reddy Tummuru, MD, Pulmonary medicine	1. Dr. P. Venkatesh<br>2. Dr. Srinivasulu.M	1. Ms. Afsha Begum<br>2. Ms. R. Jwala<br>3. Ms. T.Vinay Sree<br>4. Mr. Yashwanth Reddy<br>5. Ms. Indubala devi<br>6. Ms. G.Madhavi<br>7. Mr. E.Prashanth
St Theresa's Hospital, Hyderabad, Telangana	ECR/230/Inst/AP/2013/RR-22	Dr. A. Venkateshwar Rao, MBBS, DNB, Internal Medicine	1. Dr. G. Sainath Sunil Baba<br>2. Dr. K. Surendar Reddy<br>3. Dr. J. Sheshayamma	1. Mr. Upendra Chowdary<br>2. Ms. A.Sravani<br>3. Mr. Ch.Sai Sasidhar Raju<br>4. Mr. Md.Nishar Basha<br>5. Ms. Yamina Edula<br>6. Ms. Sujatha Sasi
V S General Hospital, Ahmedabad, Gujarat	ECR/886/Inst/GJ/2016/RR-19	Dr. Dhaiwat Shukla, MBBS, MD	1. Dr. Devang Rana<br>2. Dr. Moh. Rafe Khan Pathan<br>3. Dr. Kunal Sathwara<br>4. Dr. Bushra Quereshi<br>5. Dr. Kamal Jadav	1. Mr. Mudit Mayank<br>2. Mr. Shashank Patel<br>3. Mr. Moh. Saddam<br>4. Mr. Bhupendra Pandya
Maharaja Agrasen Super specialty Hospital, Jaipur Rajasthan	ECR/1222/Inst/RJ/2019/RR-22	Dr. Manish Kumar Jain, MBBS	1. Dr. Sanjay Kumar Sharma<br>2. Dr. P.K Sharma<br>3. Dr. Madhvender Jain	1. Mr. Khushwant Khatri<br>2. Mr. Kapil Soni<br>3. Mr. Sanjeev Kumar Vimal<br>4. Ms. Manisha Singh<br>5. Ms. Preeti Kanwar<br>6. Mr. Sunil Yogi<br>7. Ms. Mehboob Khan
All India Institute of Medical Sciences (AIIMS) Jodhpur Rajasthan.	ECR/866/Inst/RJ/2016/RR-19	Dr. Pankaj Bhardwaj, MBBS, M.D.	1. Dr. Akhil D. Goyal<br>2. Dr. Manoj Kumar Gupta<br>3. Dr. Deepak Sharma<br>4. Dr. Jay Karan Charan	1. Mr. Pyarelal Swami<br>2. Mr. Dheerak Bohra<br>3. Mr. Nipun Guar<br>4. Mr. Suraj Singh<br>5. Mr. Roop Singh<br>6. Mr. Deep Chand

Supe Heart & Diabetes Hospital and Research Centre, Nashik, Maharashtra	ECR/272/Inst/MH/2013/RR-19	Dr. Pravin Dinkar Supe, MBBS, MD	1. Dr. Pranav Vijay Deore<br>2. Dr. Manoj Damodar More	1. Ms. Subhada Shinde<br>2. Ms. Kalyani Landge<br>3. Ms. Ashwini Bhandekar<br>4. Ms. Mayuri Dilip Kamdi<br>5. Ms. Yogita Mengane<br>6. Ms. Mamata Gavit<br>7. Mr. Sayyad Afroz Sajeed<br>8. Ms. Komal Mahajan<br>9. Ms. Bharati Nana Sabale
SRMSIMS Hospital, Bareilly, Uttar Pradesh	ECR/364/Indt/UP/2022	Dr. Manoja Kumar Das	1. Dr. Abhishek Agarwal<br>2. Dr. S.B. Gupta<br>3. Dr. M.P. Rawal<br>4. Dr. Sumita Gupta<br>5. Dr. Rahul Goyal	1. Mr. Shiv Kumar<br>2. Mr. Kapil<br>3. Mr. Mukesh<br>4. Mr. Devendra Sherawat<br>5. Dr. Abdul Hafeez Khanzada<br>6. Ms. Sheetal Kumari<br>7. Mr. Pankaj Gupta
Suyash Institute of Medical Sciences Pvt Ltd, Raipur, Chhattisgarh	ECR/1546/Inst/CG/2021	Dr. Manoj Lahoti, MBBS	1. Dr.Vivek Kesharwani<br>2. Dr.Amit Joshi<br>3. Dr.Gaurav Tripathi<br>4. Dr.Bhuwan Sharma	1. Ms. Swati Singh Thakur<br>2. Ms. Ritu Mishra<br>3. Ms. Ritu Rahile<br>4. Mr. Kartikey Yadav<br>5. Mr. Rekhram Sonkar<br>6. Ms. Rashmi Chanda<br>7. Ms. Bharati Pradhan<br>8. Ms. Tarani Sahu
Rajarshee Chhatrapati Shahu Maharaj Government Medical College & Chhatrapati Pramila Raje Hospital, Kolhapur Maharashtra	ECR/703/Inst/MH/2015/RR-20	Dr. Vijaykumar Barge, MBBS	1. Dr. Vasim Mulla<br>2. Dr. Vitthal Karande<br>3. Dr. Varun Bafna<br>4. Dr. R. R. Bhosale	1. Mr. Ratan Patil<br>2. Ms. Ankita Jagtap<br>3. Mr. Shivprasad Patil<br>4. Mr. Vishwajit Patil<br>5. Ms. Nupur Shewale<br>6. Ms. Padmaja Huprikar

JSS Contributors: Dr. Jayashri Krishnan, Dr. Neeraj Prasad, Dr. Sonika Newar, Dr. Skandashree BS, Dr. Achal P Thul, Dr. Ghazanfar, Ms. Anjali Yadav, Ms. Ekta Baranwal, Ms. Palaghat Swathi.

Gennova Biopharmaceuticals Contributors: Dr. Shadab Tamboli, Ms. Himani Kumar, Ms. Kalyani Mhaske, Mr. Saurabh Mahajan, Mr. Sandeep Vishwakarma, Mr. Kshitij Gaikwad, Mr. Atul Darwatkar, Mr. Rushank Gunnale, Mr. Sunil Raut, Mr. Yogesh Phadke, Ms. Shalu Shukla,

Mr. Niket Shede, Mr. Akshay Mandlecha, Mr. Yash Sonawane, Ms. Ankita Raut, Ms. Swati Pandit, Ms. Bhavana Kharote.

We would also like to thank **PharmaJet** for not only providing us with the devices for the intra-dermal delivery of the vaccine, but also for their support in conducting the clinical trial.

Supplementary Table 2. LSGMR and seroconversion of anti-spike IgG antibodies (phase 2)

	BBV152		ChAdOx1 nCoV-19		OVERALL	
	**GEMCOVAC-OM (N=14)**	**GEMCOVAC-19 (N=14)**	**GEMCOVAC-OM (N=56)**	**GEMCOVAC-19 (N=56)**	**GEMCOVAC-OM (N=70)**	**GEMCOVAC-19 (N=70)**
**Baseline** GMT (95% CI)[a]	23227 (13801-39091)	30137 (19780-45916)	32046 (24722-41541)	37213 (29787-46491)	30048 (23910-37763)	35676 (29401-43289)
**Day 29** GMT (95% CI)[a]	229202 (197702-265721)	59528 (36959-95878)	248405 (230771-267386)	80365 (63797-101234)	244440 (229122-260782)	75683 (61687-92853)
GMFR[b]	9.87	1.98	7.75	2.16	8.13	2.12
p value[c]	< 0.0001	0.0105	< 0.0001	< 0.0001	< 0.0001	< 0.0001
LSGMR (95% CI)[d]	4.08 (2.55-6.52)		3.23 (2.60-4.02)		3.40 (2.79-4.13)	
p value[d]			< 0.0001		< 0.0001	
Seroconversion (≥ 2-fold rise from baseline,) % (95% CI)[e]	100 (76.84-100)	57.1 (28.86-82.34)	91.1 (80.38-97.04)	57.1 (43.22-70.29)	92.9 (84.11-97.64)	57.1 (44.75-68.91)
Difference in seroconversion (95% CI)[f]	42.86 (16.42-67.78)		33.93 (18.48-48.48)		35.71 (22.35-48.52)	
**Day 90** GMT (95% CI)[a]	140843 (115347-171974)	40831 (28338-58831)	166887 (142982-194788)	53875 (43950-66040)	161074 (141693-183106)	50928 (42690-60756)
GMFR[b]	6.06	1.35	5.21	1.45	5.36	1.43
p value[c]	< 0.0001	0.1575	< 0.0001	< 0.0001	< 0.0001	< 0.0001
LSGMR, 95% CI[d]	3.61 (2.44-5.35)		3.21 (2.56-4.02)		3.31 (2.72-4.02)	
p value[d]	< 0.0001		< 0.0001		< 0.0001	

a. 95% CI of GMT was calculated by taking the log base 10 transformed titres.
b. GMFR: Geometric mean fold rise was calculated as post/pre of anti-spike IgG antibodies titers.
c. p value for GMFR was calculated using a two-sided Wilcoxon sign ranked test.
d. LSGMR (95% CI) and p-value was calculated using ANCOVA with baseline values as covariates.
e. 95% CI were calculated by Clopper-Pearson Method.
f. Two-sided 95% confidence intervals for difference in proportion of subjects between groups were calculated using Miettinen-Nurminen method.

Supplementary Figure 1. Anti-spike IgG antibodies (phase 2)

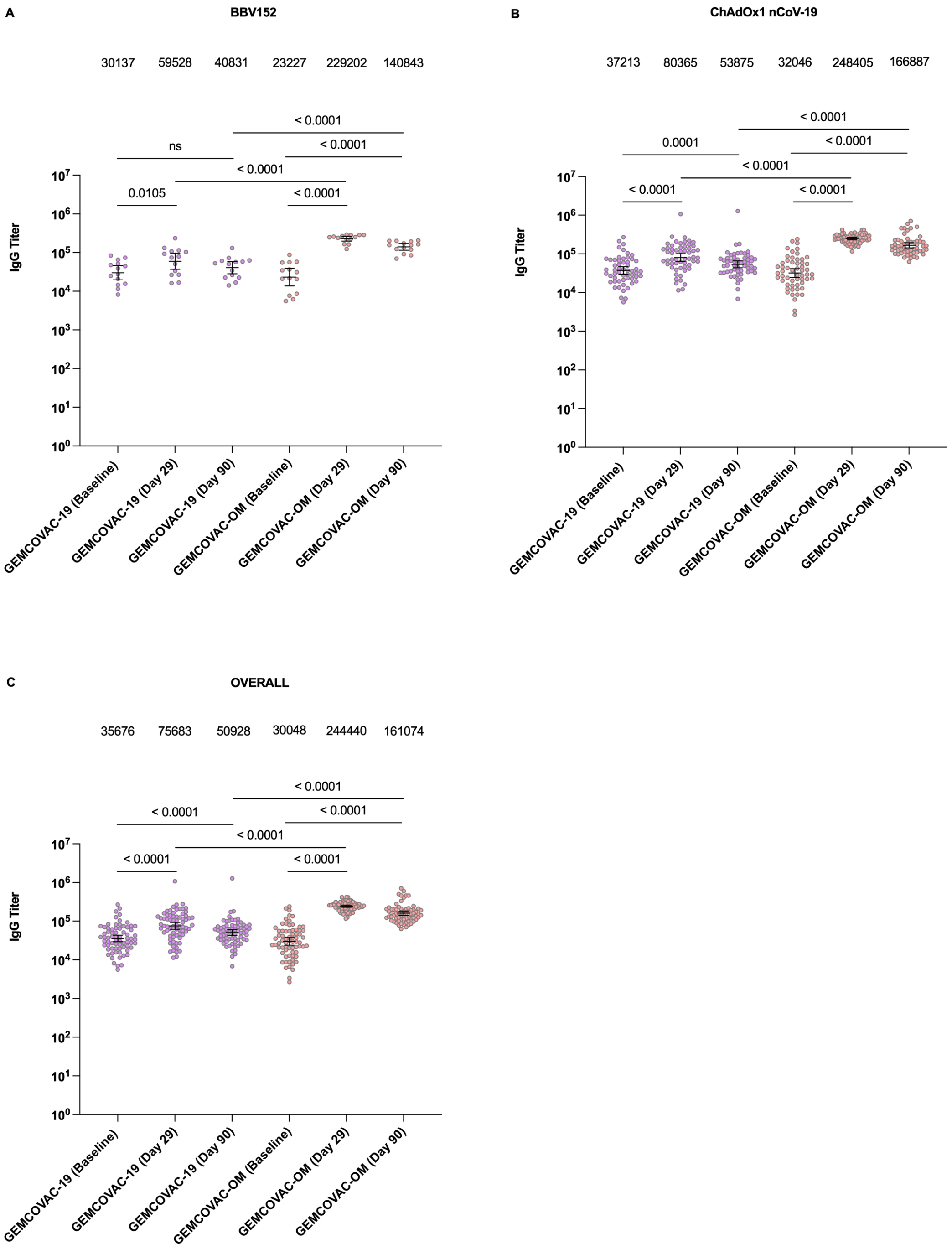

Anti-spike IgG antibodies with GEMCOVAC-19 (n = 70) and GEMCOVAC-OM (n = 70) at baseline, day 29 and day 90 in participants who received (**A**) BBV152, (**B**) ChAdOx1 nCoV-19 or (**C**) or both as their primary vaccination. Data is presented as geometric mean with 95% CI. LSGMR with 95% CI at day 29 and day 90 along with the p value was calculated using ANCOVA with baseline values as covariates. Change in titers from baseline to day 29 and day 90 was calculated by using a two-sided paired t-test or Wilcoxon sign ranked test based on normality. ns = not significant.

Supplementary Table 3. Percent neutralization by cPASS assay (phase 2)

	BBV152		ChAdOx1 nCoV-19		OVERALL	
	**GEMCOVA C-OM (N=14)**	**GEMCOVA C-19 (N=14)**	**GEMCOVA C-OM (N=56)**	**GEMCOVA C-19 (N=56)**	**GEMCOVA C-OM (N=70)**	**GEMCOVA C-19 (N=70)**
**Baseline** neutralization % (SD)	74.9 (22.69)	82.5 (13.76)	73.0 (26.49)	75.5 (24.96)	73.4 (25.63)	76.9 (23.24)
**Day 29** neutralization % (SD)	93.9 (3.82)	88.2 (12.05)	93.9 (5.71)	82.9 (19.64)	93.9 (5.36)	84.0 (18.42)
Mean % change from Baseline (SE)	16.0 (2.20)	8.7 (2.20)	20.1 (1.67)	8.3 (1.67)	19.3 (1.40)	8.4 (1.40)
P value[a]	0.0294		< 0.0001		< 0.0001	
**Day 90** neutralization % (SD)	92.9 (7.43)	85.5 (17.59)	92.2 (8.63)	80.3 (21.94)	92.4 (8.35)	81.3 (21.11)

a. ANCOVA was performed keeping the difference in mean percentage neutralization as the outcome variable, study treatment was main independent variable and baseline neutralization percentage was the covariate.
b. Pre-post vaccination comparison was done by using a two-sided paired sample t-test.

Supplementary Figure 2. Lymphocyte population (phase 2)

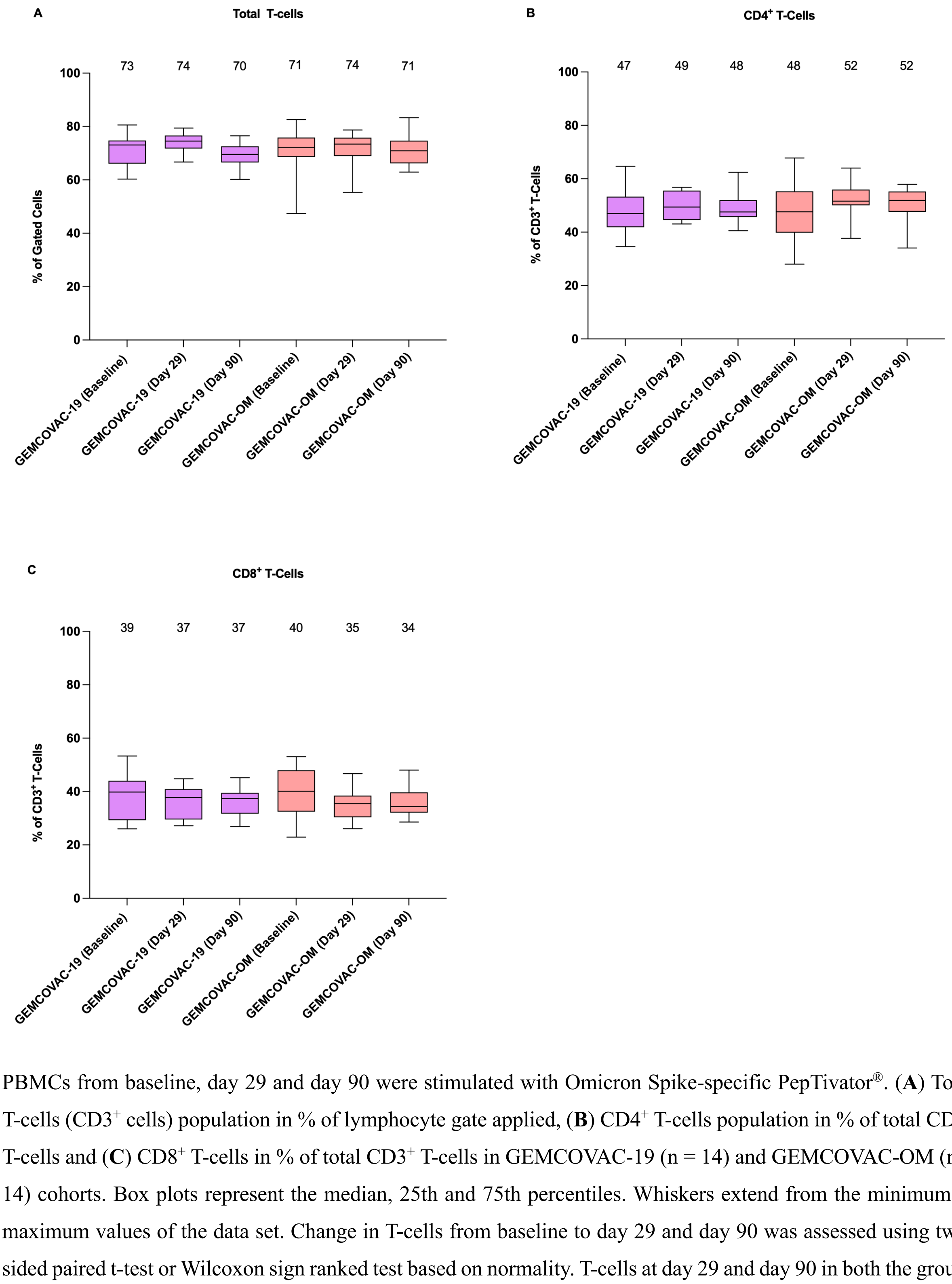

PBMCs from baseline, day 29 and day 90 were stimulated with Omicron Spike-specific PepTivator®. (**A**) Total T-cells ($CD3^+$ cells) population in % of lymphocyte gate applied, (**B**) $CD4^+$ T-cells population in % of total $CD3^+$ T-cells and (**C**) $CD8^+$ T-cells in % of total $CD3^+$ T-cells in GEMCOVAC-19 (n = 14) and GEMCOVAC-OM (n = 14) cohorts. Box plots represent the median, 25th and 75th percentiles. Whiskers extend from the minimum to maximum values of the data set. Change in T-cells from baseline to day 29 and day 90 was assessed using two-sided paired t-test or Wilcoxon sign ranked test based on normality. T-cells at day 29 and day 90 in both the groups was compared using a two-sided t-test or Wilcoxon rank sum test based on normality.

Supplementary Figure 3. Th1 cytokine producing CD4$^+$ T-cells (phase 2)

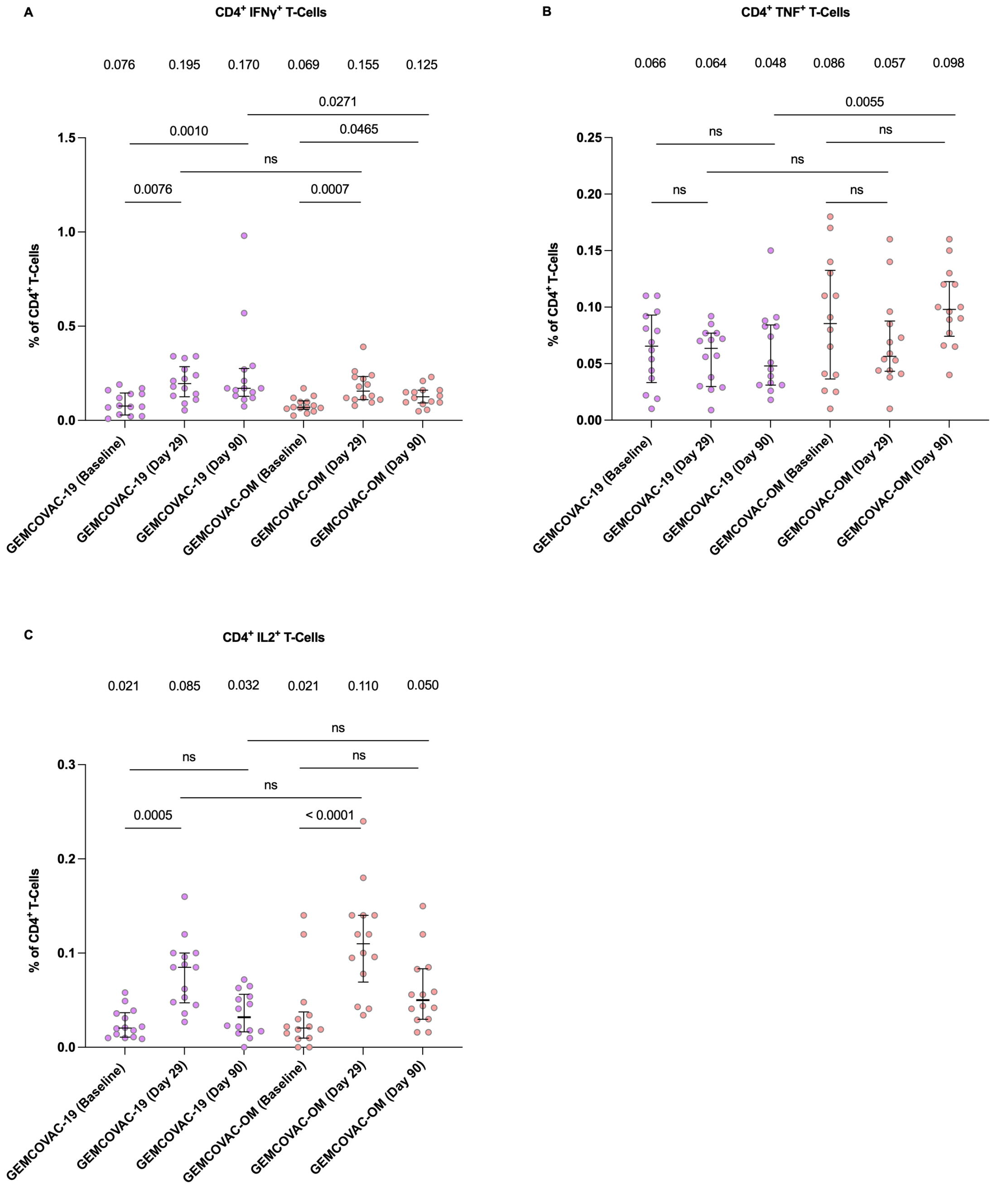

PBMCs from day 1, day 29 and day 90 were stimulated with Omicron Spike-specific PepTivator®. (**A**) IFNγ expressing CD4$^+$ T-cells, (**B**) TNF expressing CD4$^+$ T-cells and (**C**) IL-2 expressing CD4$^+$ T-cells in GEMCOVAC-19 (n = 14) and GEMCOVAC-OM (n = 14) cohorts. Data is presented as median with interquartile range (IQR). Change in cytokine expression from baseline to day 29 and day 90 was assessed using a two-sided paired t-test or Wilcoxon sign ranked test based on normality. Expression at day 29 and day 90 in both the groups was compared using a two-sided t-test or Wilcoxon rank sum test based on normality. ns = not significant.

Supplementary Figure 4. Th1 cytokine producing CD8^{+} T-cells (phase 2)

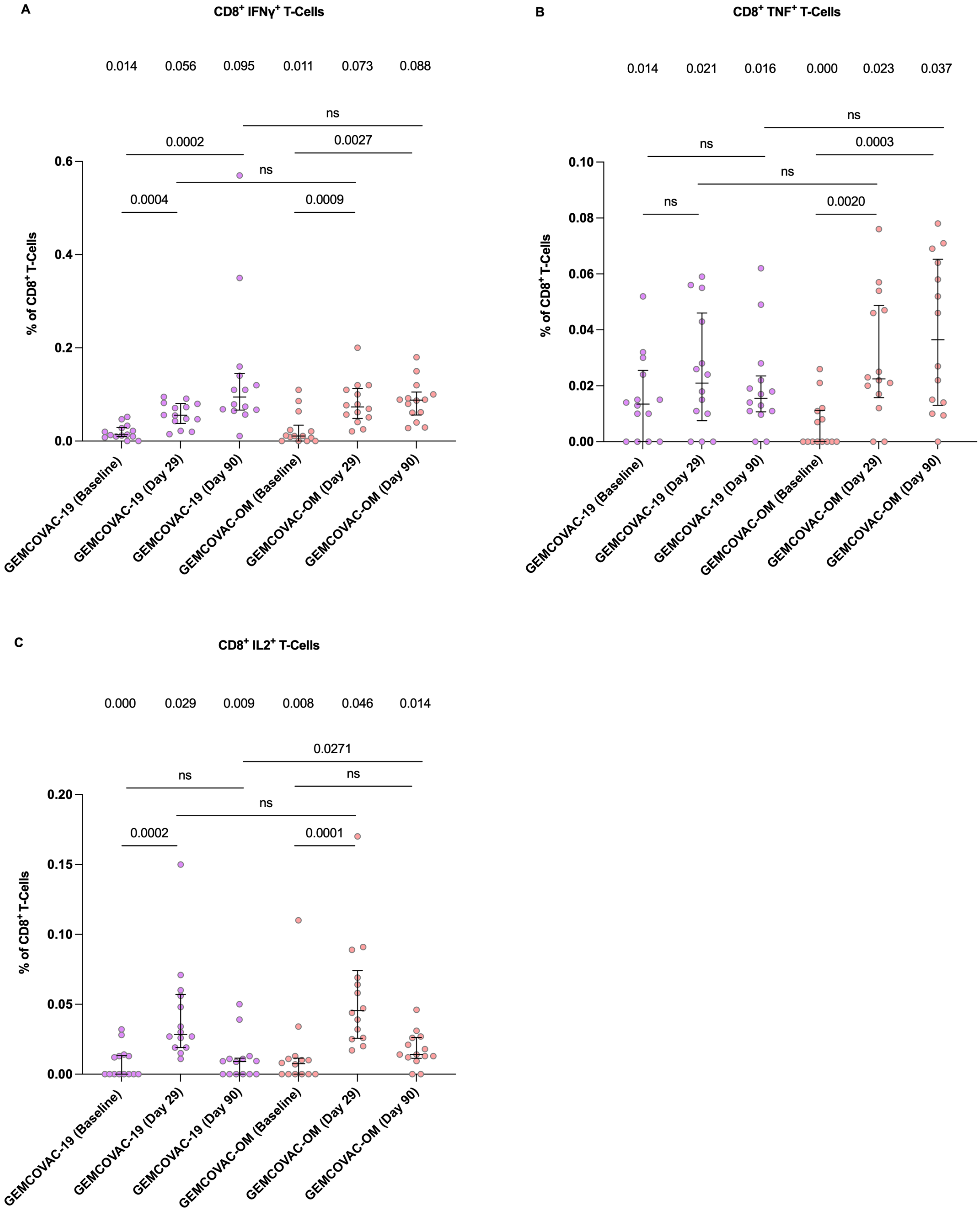

PBMCs from day 1, day 29 and day 90 were stimulated with Omicron Spike-specific PepTivator®. (**A**) IFNγ expressing CD8^{+} T-cells, (**B**) TNF expressing CD8^{+} T-cells population and (**C**) IL-2 expressing CD4^{+} T-cells in GEMCOVAC-19 (n = 14) and GEMCOVAC-OM (n = 14) cohorts. Data is presented as median with interquartile range (IQR). Change in cytokine expression from baseline to day 29 and day 90 was assessed using a two-sided paired t-test or Wilcoxon sign ranked test based on normality. Expression at day 29 and day 90 in both the groups was compared using a two-sided t-test or Wilcoxon rank sum test based on normality. ns = not significant.

Supplementary Figure 5. Th2 cytokine producing CD4+ and CD8+ T cells (phase 2)

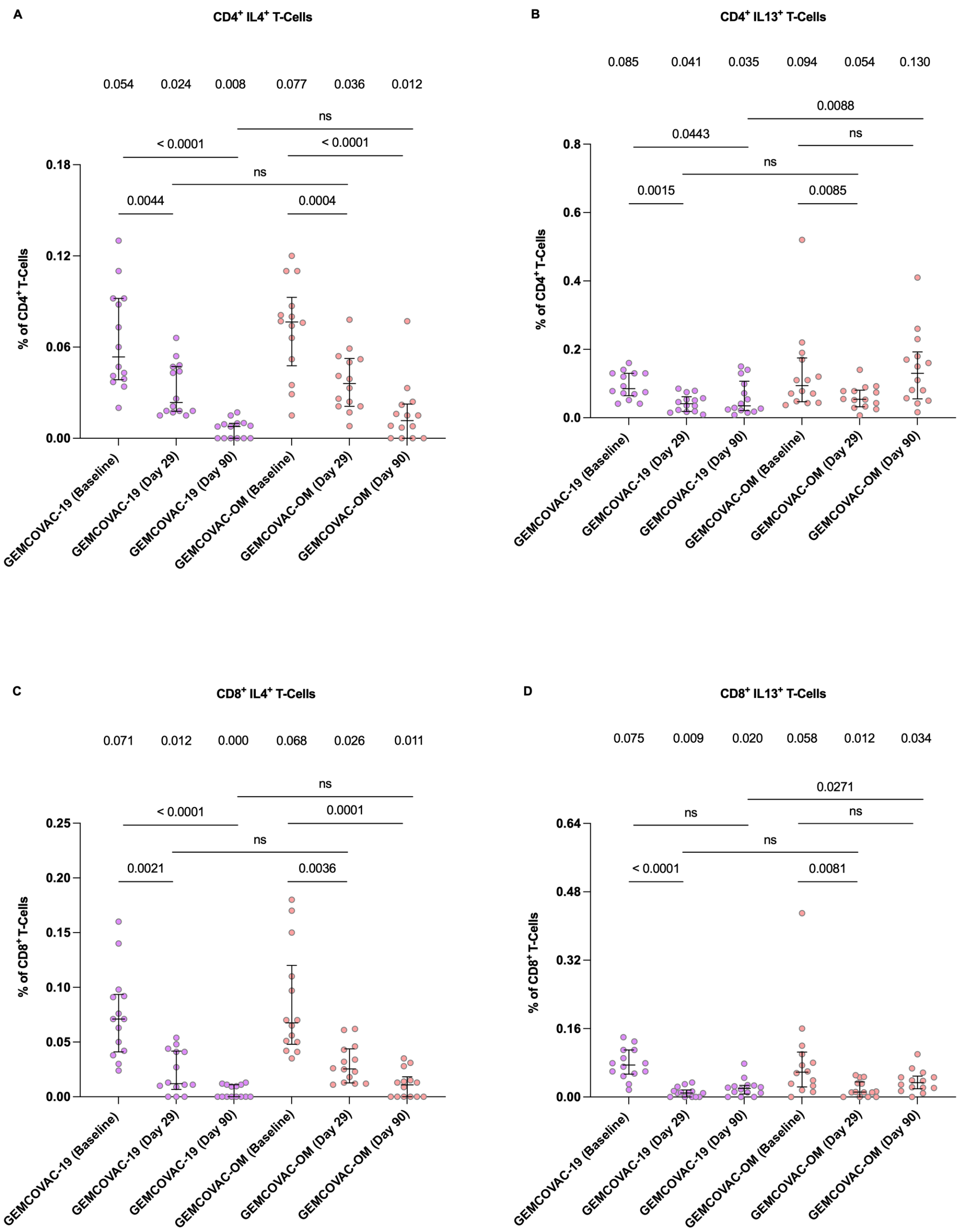

PBMCs from day 1, day 29 and day 90 were stimulated with Omicron Spike-specific PepTivator®. (**A**) IL-4 expressing CD4+ T-cells, (**B**) IL-13 expressing CD4+ T-cells, (**C**) IL-4 expressing CD8+ T-cells and (**D**) IL-13 expressing CD8+ T-cells population in GEMCOVAC-19 (n = 14) and GEMCOVAC-OM (n = 14) cohorts. Data is presented as median with interquartile range (IQR). Change in cytokine expression from baseline to day 29 and day 90 was assessed using a two-sided paired t-test or Wilcoxon sign ranked test based on normality. Expression at day 29 and day 90 in both the groups was compared using a two-sided t-test or Wilcoxon rank sum test based on normality. ns = not significant.

Supplementary Table 4. Summary of adverse events (phase 2)

	BBV152		ChAdOx1 nCoV-19		OVERALL	
**SOC/PT, n (%)**	**GEMCOVAC-OM (N=14)**	**GEMCOVAC-19 (N=14)**	**GEMCOVAC-OM (N=56)**	**GEMCOVAC-19 (N=56)**	**GEMCOVAC-OM (N=70)**	**GEMCOVAC-19 (N=70)**
TOTAL (ALL SOC/PT)	0 (0.0%)	1 (7.1%)	5 (8.9%)	8 (14.3%)	5 (7.1%)	9 (12.9%)
Fatigue	0 (0.0%)	0 (0.0%)	0 (0.0%)	1 (1.8%)	0 (0.0%)	1 (1.4%)
Pain at site	0 (0.0%)	1 (7.1%)	3 (5.4%)	4 (7.1%)	3 (4.3%)	5 (7.1%)
Pyrexia	0 (0.0%)	0 (0.0%)	0 (0.0%)	1 (1.8%)	0 (0.0%)	1 (1.4%)
Myalgia	0 (0.0%)	0 (0.0%)	1 (1.8%)	1 (1.8%)	1 (1.4%)	1 (1.4%)
Headache	0 (0.0%)	0 (0.0%)	1 (1.8%)	1 (1.8%)	1 (1.4%)	1 (1.4%)

Supplementary Table 5. LSGMR and seroconversion of neutralizing antibodies (phase 3)

	BBV152	ChAdOx1 nCoV-19		OVERALL	
	**GEMCOVAC-OM (N=78)**	**GEMCOVAC-OM (N=193)**	**ChAdOx1 nCoV-19 (N=133)**	**GEMCOVAC-OM (N=271)**	**ChAdOx1 nCoV-19 (N=133)**
**Baseline** GMT (95% CI)[a]	511.0 (381.5-684.3)	676.4 (561.3-815.0)	775.3 (620.2-969.2)	623.9 (533.3-729.9)	775.3 (620.2-969.26)
**Day 29** GMT (95% CI)[a]	1043.9 (869.7-1253.1)	1123.4 (1003.8-1257.2)	754.9 (631.5-902.5)	1099.9 (1000.0-1209.9)	754.9 (631.5-902.5)
GMFR[b]	2.04	1.66	0.97	1.76	0.97
p value[c]	0.0006	0.0004	0.149	< 0.0001	0.149
LSGMR (95% CI)[d]		1.57 (1.33-1.85)		1.58 (1.36-1.84)	
p value[d]		< 0.0001		< 0.0001	
Seroconversion assessed by ≥ 2-fold rise from baseline, % (95% CI)[e]	43.6 (32.3-55.3)	37.8% (30.9-45.0)	19.5 (13.1-27.3)	39.5 (33.6-45.5)	19.5 (13.127.3)
Difference in seroconversion (95% CI)		18.27 (8.34-27.60)		19.93 (10.57-28.43)[f]	
**Day 90** GMT (95% CI)[a]	820.4 (665.2-1011.6)	728.8 (650.6-816.4)	383.1 (319.4-459.6)	754.0 (682.1-833.6)	383.1 (319.4-459.6)
GMFR[b]	1.61	1.08	0.49	1.21	0.49
p value[c]	0.0408	0.0423	< 0.0001	0.5618	< 0.0001
LSGMR (95% CI)[d]		1.97 (1.64-2.37)		2.09 (1.75-2.49)	
p value[d]		< 0.0001		< 0.0001	

a. 95% CI of GMT was calculated by taking the log base 10 transformed titres.
b. GMFR: Geometric mean fold rise was calculated as post/pre of neutralizing antibodies titers.
c. p value for GMFR was calculated using a two-sided Wilcoxon sign ranked test.
d. LSGMR: Least Square Geometric Mean Ratio (95% CI) and p-value was calculated using ANCOVA with baseline values as covariates.
e. 95% CI were calculated by Clopper-Pearson Method.
f. Two-sided 95% confidence intervals for difference in proportion of subjects between groups were calculated using Miettinen-Nurminen method.

Supplementary Table 6. LSGMR and seroconversion of anti-spike IgG antibodies (phase 3)

	BBV152	ChAdOx1 nCoV-19		OVERALL	
	GEMCOVAC-OM (N=78)	GEMCOVAC-OM (N=193)	ChAdOx1 nCoV-19 (N=133)	GEMCOVAC-OM (N=271)	ChAdOx1 nCoV-19 (N=133)
**Baseline** GMT (95% CI)[a]	28736 (22858-36126)	41351 (36677-46621)	39206 (34052-45140)	37239 (33398-41522)	39206 (34052-45140)
**Day 29** GMT (95% CI)[a]	262673 (210500-327777)	273022 (244074-305404)	128916 (109548-151707)	270002 (243972-298810)	128916 (109548-151707)
GMFR[b]	9.14	6.60	3.29	7.25	3.29
p value[c]	< 0.0001	< 0.0001	< 0.0001	< 0.0001	< 0.0001
LSGMR (95% CI)[d]		2.06 (1.75:2.42)		2.15 (1.83:2.52)	
p value[d]		< 0.0001		< 0.0001	
Seroconversion (≥ 2-fold rise from baseline,) % (95% CI)[e]	93.6 (85.6-97.8)	92.7 (88.1-95.9)	76.7 (68.5-83.5)	93.0 (89.2-95.7)	76.7 (68.583.5)
Difference in seroconversion (95% CI)[f]		16.05 (8.34-24.57)		16.30 (9.02-24.64)	
**Day 90** GMT (95% CI)[a]	156008 (126196-192862)	137650 (121006-156582)	65182 (55341-76773)	142700 (127874-159246)	65182 (55341-76773)
GMFR[b]	5.43	3.33	1.66	3.83	1.66
p value[c]	< 0.0001	< 0.0001	< 0.0001	< 0.0001	< 0.0001
LSGMR (95% CI)[d]		2.05 (1.70-2.48)		2.23 (1.87-2.66)	
p value[d]		< 0.0001		< 0.0001	

a. 95% CI of GMT was calculated by taking the log base 10 transformed titres.
b. GMFR: Geometric mean fold rise was calculated as post/pre of anti-spike IgG antibodies titers.
c. p value for GMFR was calculated using a two-sided Wilcoxon sign ranked test.
d. LSGMR (95% CI) and p-value was calculated using ANCOVA with baseline values as covariates.
e. 95% CI were calculated by Clopper-Pearson Method.
f. Two-sided 95% confidence intervals for difference in proportion of subjects between groups were calculated using Miettinen-Nurminen method.

Supplementary Table 7. Percent neutralization by cPass assay (phase 3)

	BBV152	ChAdOx1 nCoV-19		OVERALL	
	GEMCOVAC-OM (N=78)	GEMCOVAC-OM (N=193)	ChAdOx1 nCoV-19 (N=133)	GEMCOVAC-OM (N=271)	ChAdOx1 nCoV-19 (N=133)
**Baseline** neutralization % (SD)	62.2 (26.79)	70.5 (26.90)	68.6 (26.10)	68.1 (27.07)	68.6 (26.10)
**Day 29** neutralization % (SD)	89.5 (16.23)	95.8 (7.92)	94.3 (12.26)	94.0 (11.30)	94.3 (12.26)
Mean % change from Baseline (SE)	27.3 (1.72)	26.0 (0.69)	24.7 (0.83)	25.7 (0.67)	26.0 (0.96)
P value[a]		0.2228		0.8559	
**Day 90** neutralization % (SD)	92.8 (10.68)	91.3 (11.75)	81.3 (19.74)	91.7 (11.45)	81.3 (19.74)

a. ANCOVA was performed keeping the difference in mean percentage neutralization as the outcome variable, study treatment was main independent variable and baseline neutralization percentage was the covariate.
b. Pre-post vaccination comparison was done by using a two-sided paired sample t-test.

Supplementary Figure 6. Live Virus Neutralization by PRNT (phase 3)

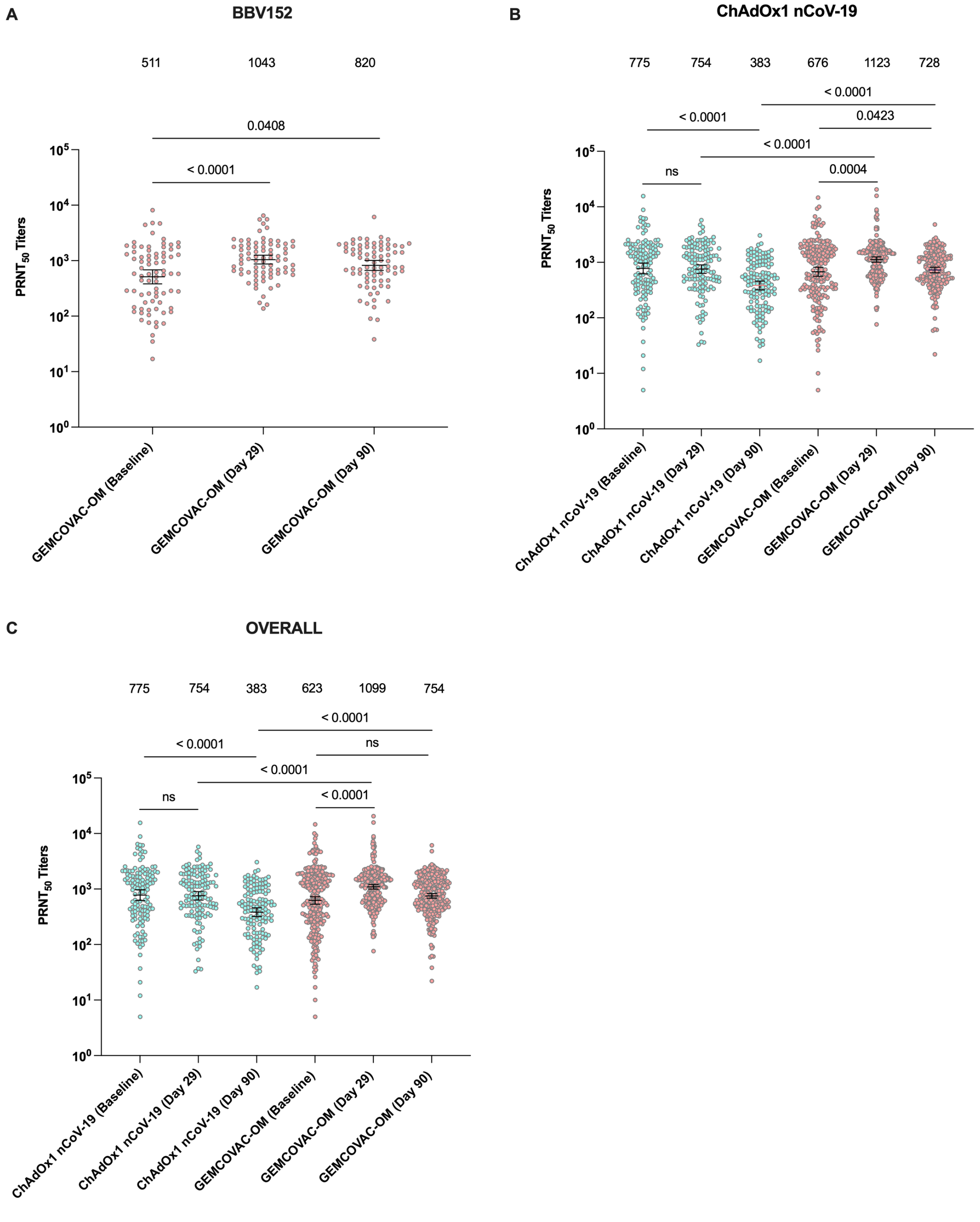

$PRNT_{50}$ titers at baseline, day 29 and day 90 in ChAdOx1 nCoV-19 (n = 133) and GEMCOVAC-OM (n = 271) cohorts who previously received 2 doses of (**A**) BBV152, (**B**) ChAdOx1 nCoV-19 or (**C**) both as their primary vaccination. Data is presented as geometric mean with 95% CI. LSGMR with 95% CI at day 29 and day 90 along with the p value was calculated using ANCOVA with baseline values as covariates. Change in titers from baseline to day 29 and day 90 was calculated by using a two-sided paired t-test or Wilcoxon sign ranked test based on normality. ns = not significant.

Supplementary Figure 7. Anti-spike IgG antibodies (phase 3)

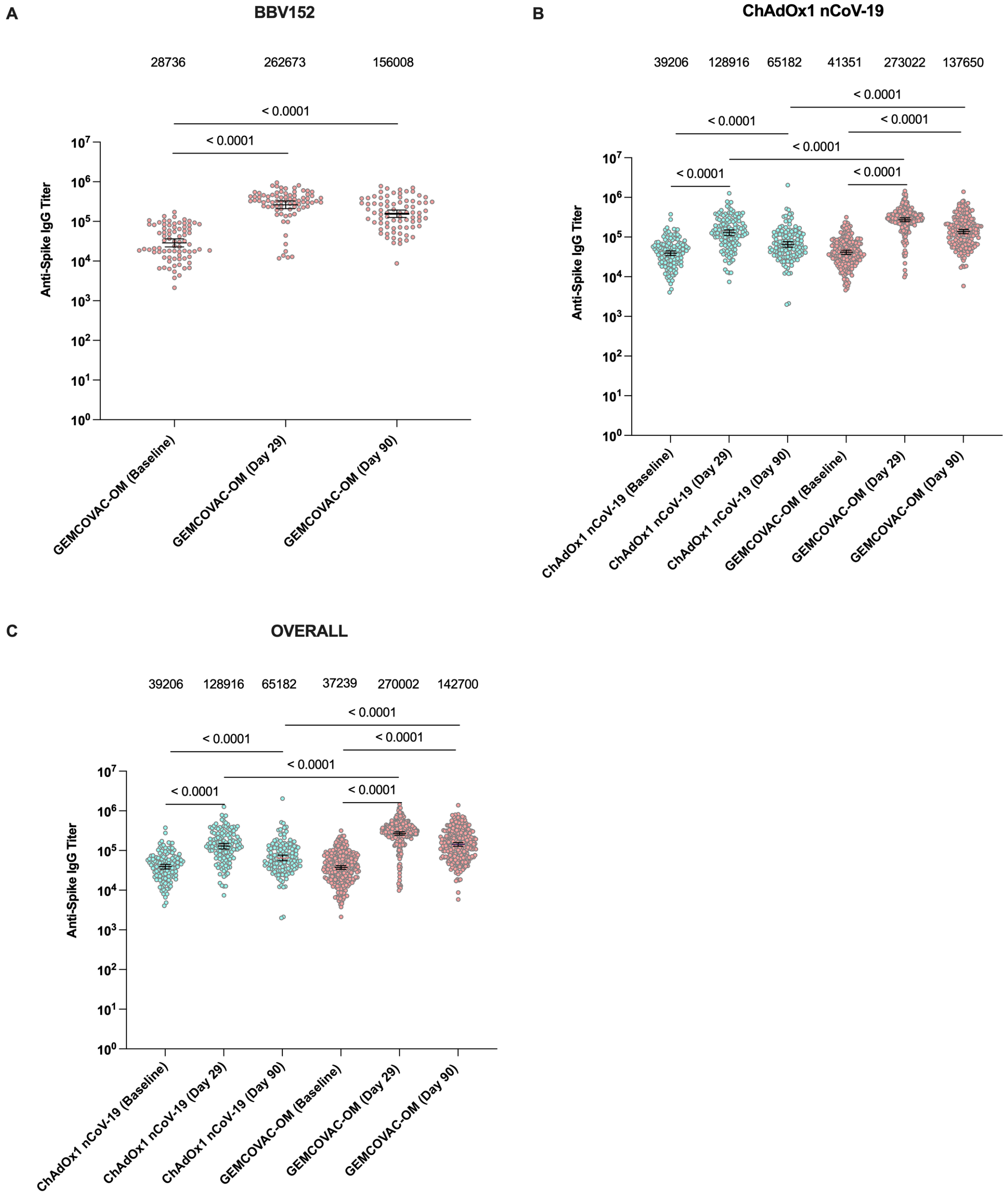

Anti-spike IgG titers at baseline, day 29 and day 90 in ChAdOx1 nCoV-19 (n = 133) and GEMCOVAC-OM (n = 271) cohorts who previously received 2 doses of (**A**) BBV152, (**B**) ChAdOx1 nCoV-19 or (**C**) both as their primary vaccination. Data is presented as geometric mean with 95% CI. LSGMR with 95% CI at day 29 and day 90 along with the p value was calculated using ANCOVA with baseline values as covariates. Change in titers from baseline to day 29 and day 90 was calculated by using a two-sided paired t-test or Wilcoxon sign ranked test based on normality. ns = not significant.

Supplementary Figure 8. Adverse events (phase 3)

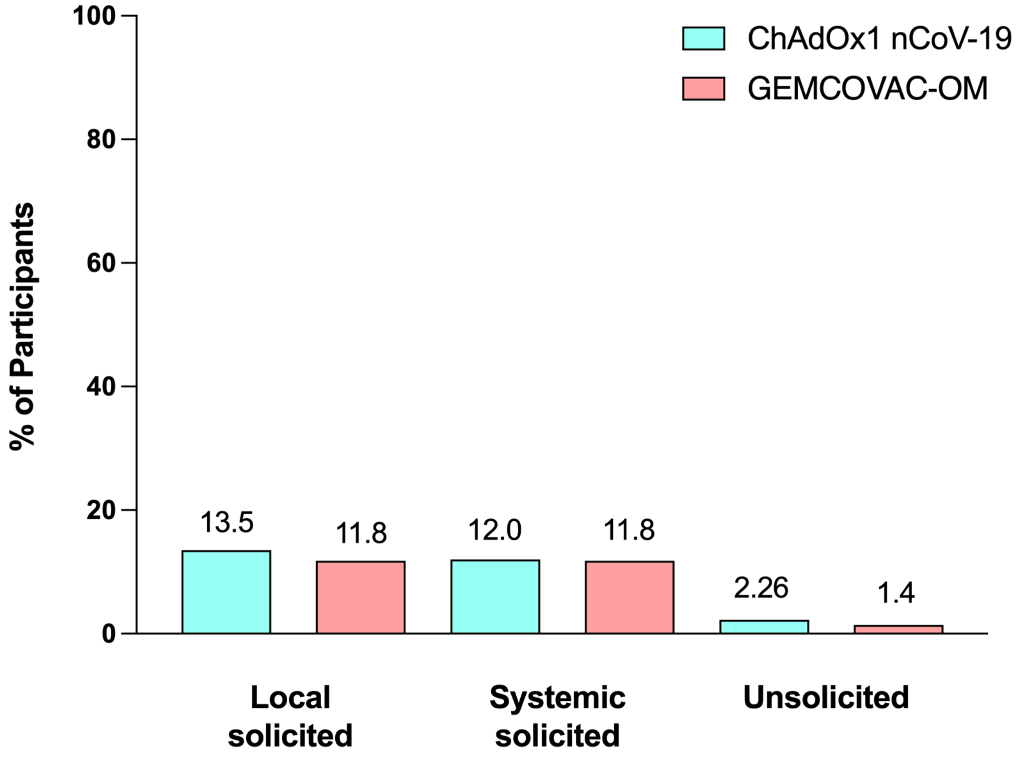

Supplementary Table 8. Demography disaggregated by sex (phase 3)

	GEMCOVAC-OM (N=2990)		ChAdOx1 nCoV-19 (N=133)	
	**Male (N=2039)**	**Female (N=951)**	**Male (N=106)**	**Female (N=27)**
Age, median years (range)	31 (24-39)	34 (25-43)	30 (26-34)	37 (29-43)
Weight, mean in kg (SD)	64.9 (9.49)	57.8 (9.66)	65.1 (10.02)	59.2 (11.79)
BMI, mean (SD)	23.4 (3.17)	23.8 (3.92)	23.4 (3.21)	25.1 (4.36)

Supplementary Table 9. Neutralizing antibodies disaggregated by sex (phase 3)

	GEMCOVAC-OM (N=271)		ChAdOx1 nCoV-19 (N=133)	
	Male (N=206)	Female (N=65)	Male (N=106)	Female (N=27)
**Baseline** GMT (95% CI)[a]	556.5 (464.5-666.8)	896.4 (659.3-1218.7)	655.8 (507.9-846.7)	1496.5 (1023.0-2189.1)
**Day 29** GMT (95% CI)[a]	1073.7 (961.0-1199.8)	1187.3 (983.0-1434.1)	685.4 (557.3-842.8)	1103.4 (800.8-1520.2)
GMFR[b]	1.93 (1.65-2.25)	1.32 (1.04-1.68)	1.05 (0.84-1.29)	0.74 (0.53-1.03)
p value[c]	< 0.0001	0.9254	0.8008	0.0197
LSGMR 95% CI[d]	1.06 (0.88-1.28)		0.91 (0.6-1.32)	
p value[d]	0.5469		0.6253	
Seroconversion % (95% CI)[e]	41.7 (34.9-48.80)	32.3 (21.2-45.05)	21.7 (14.28-30.76)	11.1 (2.35:29.16)
Difference in seroconversion (95% CI)[f]	9.44		10.59	
**Day 90** GMT (95% CI)[a]	694.0 (616.9-780.90)	980.4 (820.1-1172.0)	356.1 (289.0-439.0)	508.7 (352.8-733.6)
GMFR[b]	1.25 (1.04-1.49)	1.09 (0.82-1.46)	0.54 (0.44-0.70)	0.34 (0.24-0.49)
p value[c]	0.7971	0.1136	< 0.0001	< 0.0001
LSGMR 95% CI[d]	0.79 (0.63-0.98)		0.98 (0.65-1.48)	
p value[d]	0.0333		0.9315	

a. 95% CI of GMT was calculated by taking the log base 10 transformed titres.
b. GMFR: Geometric mean fold rise was calculated as post/pre of neutralizing antibodies titers.
c. p value for GMFR was calculated using a two-sided Wilcoxon sign ranked test.
d. LSGMR: Least Square Geometric Mean Ratio (95% CI) and p-value was calculated using ANCOVA with baseline values as covariates.
e. 95% CI were calculated by Clopper-Pearson Method.
f. Two-sided 95% confidence intervals for difference in proportion of subjects between groups were calculated using Miettinen-Nurminen method.

Supplementary Figure 9. Neutralizing antibodies disaggregated by sex (phase 3).

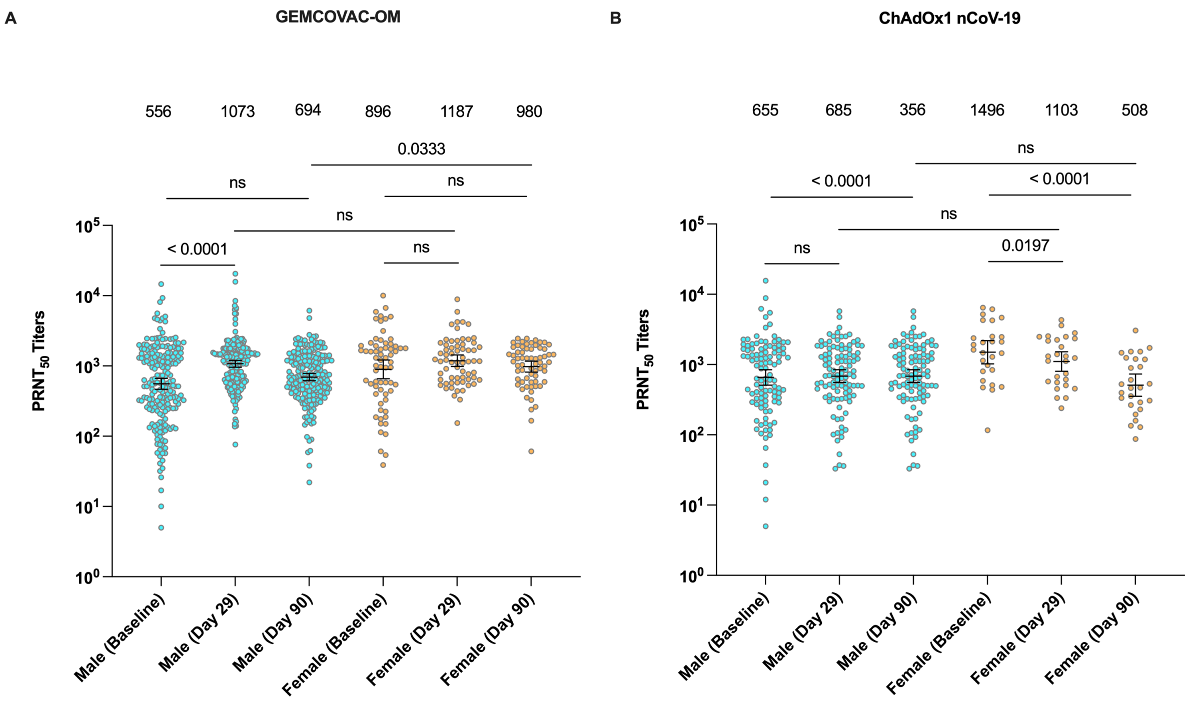

Neutralizing antibodies with disaggregated by sex in (**A**) GEMCOVAC-OM (n = 271) and (**B**) ChAdOx1 nCoV-19 (n = 133). Data is presented as geometric mean with 95% CI. LSGMR with 95% CI at day 29 and day 90 along with the p value was calculated using ANCOVA with baseline values as covariates. Change in titers from baseline to day 29 and day 90 was calculated by using a two-sided paired t-test or Wilcoxon sign ranked test based on normality. ns = not significant.

Supplementary Table 10. Anti-spike IgG antibodies disaggregated by sex (phase 3)

	GEMCOVAC-OM (N=271)		ChAdOx1 nCoV-19 (N=133)	
	**Male (N=206)**	**Female (N=65)**	**Male (N=106)**	**Female (N=27)**
**Baseline** GMT (95% CI)[a]	33818 (29749-38443)	50537 (41833-61051)	38528 (32865-45166)	41989 (30374-58045)
**Day 29** GMT (95% CI)[a]	249887 (220849-282744)	345086 (297380-400444)	126760 (106125-151408)	137736 (90446-209754)
GMFR[b]	7.39 (6.50-8.40)	6.83 (5.63-8.29)	3.29 (2.81-3.85)	3.28 (2.31-4.65)
p value[c]	< 0.0001	< 0.0001	< 0.0001	< 0.0001
LSGMR 95% CI[d]	0.86 (0.69-1.06)		0.97 (0.69-1.37)	
p value[d]	0.1623		0.8701	
Seroconversion % (95% CI)[e]	92.7 (88.27-95.87)	93.8 (84.99-98.30)	76.4 (67.18-84.12)	77.8 (57.74-91.38)
Difference in seroconversion (95% CI)[f]	-1.13		-1.36	
**Day 90** GMT (95% CI)[a]	133040 (116968-151320)	178199 (145488-218265)	63419 (52678-76350)	72519 (50253-104650)
GMFR[b]	3.93 (3.42-4.52)	3.53 (2.79-4.46)	1.65 (1.39-1.98)	1.73 (1.20-2.48)
p value[c]	< 0.0001	< 0.0001	< 0.0001	0.0033
LSGMR 95% CI[d]	0.87 (0.68-1.11)		0.92 (0.64-1.32)	
p value[d]	0.2600		0.6504	

a. 95% CI of GMT was calculated by taking the log base 10 transformed titres.
b. GMFR: Geometric mean fold rise was calculated as post/pre of neutralizing antibodies titers.
c. p value for GMFR was calculated using a two-sided Wilcoxon sign ranked test.
d. LSGMR: Least Square Geometric Mean Ratio (95% CI) and p-value was calculated using ANCOVA with baseline values as covariates.
e. 95% CI were calculated by Clopper-Pearson Method.
f. Two-sided 95% confidence intervals for difference in proportion of subjects between groups were calculated using Miettinen-Nurminen method.

Supplementary Figure 10. Anti-spike IgG antibodies disaggregated by sex (phase 3).

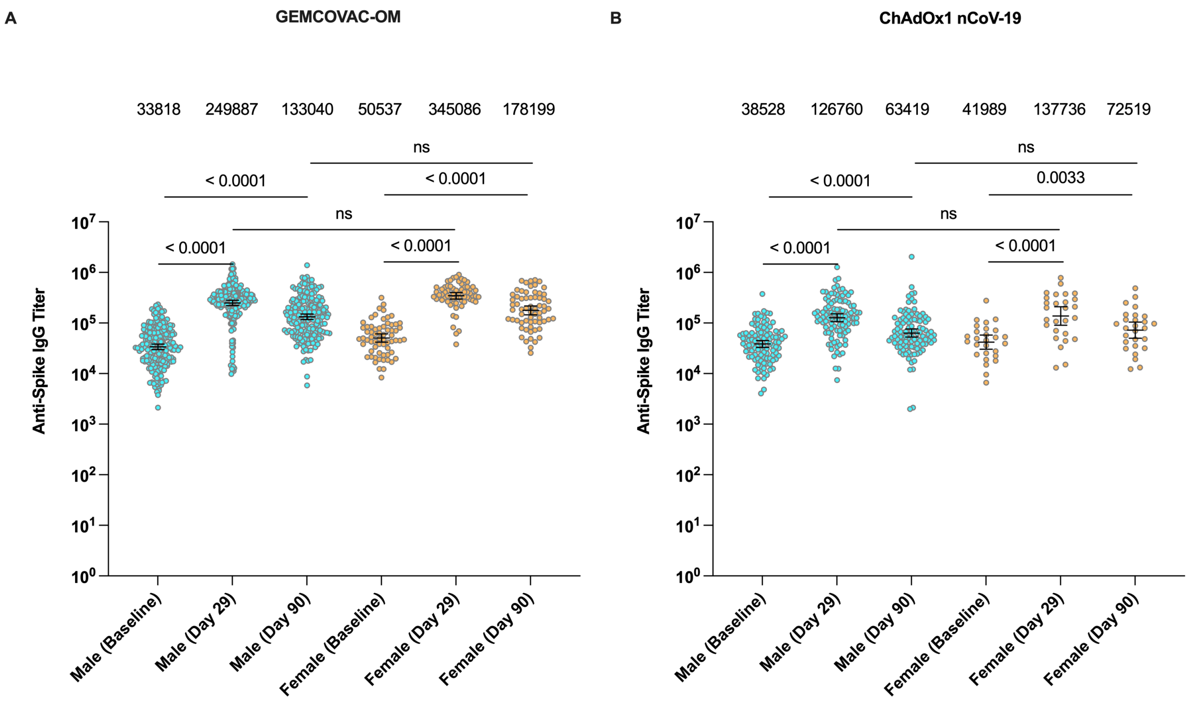

Anti-spike IgG antibodies with disaggregated by sex in (**A**) GEMCOVAC-OM (n = 271) and (**B**) ChAdOx1 nCoV-19 (n = 133). Data is presented as geometric mean with 95% CI. LSGMR with 95% CI at day 29 and day 90 along with the p value was calculated using ANCOVA with baseline values as covariates. Change in titers from baseline to day 29 and day 90 was calculated by using a two-sided paired t-test or Wilcoxon sign ranked test based on normality. ns = not significant.

Supplementary Table 11. Sex-disaggregated adverse events (phase 3)

	**GEMCOVAC-OM (N=2990, Male=2039, Female=951)**		**ChAdOx1 nCoV-19 (N=133, Male=106, Female=27)**	
	Unadjusted Odds Ratio of male vs female (95% CI)	P value	Unadjusted Odds Ratio of male vs female (95% CI)	P value
Any adverse event	0.71 (0.61-0.83)	< 0.0001	1.65 (0.65-4.20)	0.2907
Local AE	0.78 (0.64-0.96)	0.0191	0.70 (0.23-2.10)	0.5267
Systemic AE	0.73 (0.60-0.90)	0.0027	5.67 (0.72-44.06)	0.0972

2. Antibodies, reagents, cells, and virus used for analysis.

Supplementary Table 12. List of antibodies, reagents, cells, and virus used for analysis.

REAGENT/ RESOURCE	SOURCE	IDENTIFIER
**Antibodies**		
SK7 [CD3] (PE CY7)	BD Biosciences	Cat# 557851 RRID: AB_396896
SK3 [CD4] (BV480)	BD Biosciences	Cat# 566104 RRID: AB_2739506
RPA-T8 [CD8] (FITC)	BD Biosciences	Cat# 555366
B27 [IFNg] (PE)	BD Biosciences	Cat# 559327 RRID: AB_397224
MAb11 [TNFa] (APC)	BD Biosciences	Cat# 551384 RRID: AB_2204110
5344.111 [IL2] (BV421)	BD Biosciences	Cat# 562914 RRID: AB_2737888
MP4-25D2 [IL4] (BV786)	BD Biosciences	Cat# 564113 RRID: AB_2738601
JES10-5A2 [IL13] (BV711)	BD Biosciences	Cat# 564288 RRID: AB_2738731
HIB19 [CD19] (PerCP-Cy5.5)	BD Biosciences	Cat# 561295 RRID: AB_10644017
[CD28/49d]	BD Biosciences	Cat# 347690
SK7 [CD3] (BV605)	BD Biosciences	Cat# 563219 RRID: AB_2714001
2H7 [CD20] (APC-H7)	BD Biosciences	Cat# 560734 RRID: AB_1727449
Anti-Human IgG (Fc specific) −Peroxidase antibody	Merck	Cat#A0170
**Reagents, peptides, and recombinant proteins**		
PepTivator® SARS-COV-2 Prot_S B.1.1.529/BA.1 Mutation Pool	Milteyni Biotec	Cat# 130-129-928
Recombinant SARS-CoV-2 Spike-Prot B.1.1.529/BA.1 (HEK)-Biotin	Milteyni Biotec	Cat# 130-130-417
Brilliant Stain Buffer	BD Biosciences	Cat# 566349, RRID: AB_2869750
Dulbecco's phosphate buffer saline (DPBS)	Merck	Cat# D8537
RPMI1640 culture medium (RPMI)	Merck	Cat# R8758
Penicillin-Streptomycin	Thermofisher Scientific	Cat# 15140-122
Heat Inactivated fetal bovine serum (HI-FBS)	Thermofisher Scientific	Cat# 16140-071
BD GolgiPlug™	BD Biosciences	Cat# 555029
BD GolgiStop™	BD Biosciences	Cat# 554724
eBioscience™ Cell Stimulation Cocktail (500X)	Thermofisher Scientific	Cat# 00-4970-93
Cytofix/Cytoperm™ Plus fixation/Permeabilization Kit	BD Biosciences	Cat# 555028

autoMACS® Running Buffer – MACS® Separation Buffer	Milteyni Biotec	Cat# 130-091-221
BD® FC Beads 7-Color Kit	BD Biosciences	Cat# 656867
BD® FC Beads 5-Color Kit	BD Biosciences	Cat# 661564
BD® FC Beads 2-Color Kit	BD Biosciences	Cat# 662996
BD® CS&T Beads	BD Biosciences	Cat# 656505
Human AB serum	Merck	Cat# H4522
Sterile water	Merck	W4502
Streptavidin, PE	Miltenyi Biotech	Cat# 130-106-789
SARS-CoV-2 B.1.1.529 (Omicron) S1+S2 trimer Protein (ECD, His Tag)	Sino biologicals	Cat#40589-V08H26
TMB substrate	Surmodics	Cat# TMBW-1000-01
Blotting grade blocker (Non-fat dry milk)	Bio-Rad	Cat# 1706404
Minimum Essential Medium	Thermofisher Scientific	Cat#11095114
Foetal Bovine Serum	Thermofisher Scientific	Cat#16000044
Penicillin-Streptomycin	Thermofisher Scientific	Cat#15140-122
Aquacide	Merck	Cat#17851
Formaldehyde	Merck	Cat#F1635-4L
Crystal violet	Merck	Cat#C0775
**Virus strains**		
SARS-CoV-2-IND/0005/2022 (B.1.1.529.1 lineage i.e Omicron BA.1 variant)	Virus isolated from clinical sample of COVID-19 patient infected with Omicron BA.1 variant. Propagated and characterized in-house at IRSHA.	Not applicable
**Cell lines**		
Vero CCL81	ATCC	CCL-81
**Tubes and strainers**		
MACS SmartStrainer (70 µm)	Milteyni Biotec	Cat# 130-098-462
MACS SmartStrainer (30 µm)	Milteyni Biotec	Cat# 130-110-915
BD Vacutainer® CPT™ Mononuclear Cell Preparation Tube	BD Biosciences	Cat# 362761

3. Self-Amplifying mRNA Vaccine Platform for GEMCOVAC-OM

The self-amplifying mRNA (SAM) platforms by nature is designed to amplify the sub-genomic mRNA. Upon vaccination or introduction of the “+” strand of mRNA (in the form of vaccine), the polycistronic stretch P1234 (**Supplementary Figure 11**) is translated from non-structural regions, NSP1, NSP, NSP3, and NSP4. Later, it undergoes proteolytic cleavage via NSP2 and forms a strand of replicase complex (P123 and P4). The P123 and P4 initiate the synthesis of “-” strand of mRNA, and it serves as a template for subsequent synthesis. Collectively, these proteins perform the function of RNA-dependent-RNA-polymerase (RdRp) which has a proofreading activity that determines how correctly the subsequent amplified products are generated. The confirmation of generation of the minus strand can be established through a few approaches.

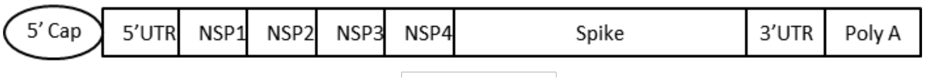

Supplementary Figure 11. Schematic of mRNA-628.2.

We have recently published our research entitled “Evaluation of self-amplifying mRNA platform for protein expression and genetic stability: Implication for mRNA therapies” in the Biochemical and Biophysical Research Communications 680 (2023) 108–118. The article focuses on highlighting the advantages of the SAM-based platform and providing more evidence for its long-term safety and genetic stability.

In this study, first, the longevity and proofreading of the SAM-based platform was assessed. Self-amplification of the antigenic mRNA is a function of nsPs that are provided in *cis*- or *trans*-design. A *cis*-cassette (mRNA-614) containing all five structural motifs of the mRNA was designed. The ORF of our designed cassette of mRNA-614 contains the genetic elements to encode the nsPs from the TC-83 VEEV strain followed by the spike protein of the SARS-CoV-2 B.1 variant. The spike protein expression was encoded by mRNA-614 into Expi-293F™ up to day 10 post-transfection. Further, proofreading activity of SAM-encoded RNA-dependent-RNA-polymerase was verified by sequencing the mRNA transcripts generated inside the transfected cells till day 5. Further, the surface expression of the spike in the

transfected HEK-293T cells was confirmed by FACS as mRNA-614 was designed in a way to express the spike protein on the surface membrane of the cells.

The second section of the study was designed to verify the safety of SAM by evaluating the genomic integration of the mRNA-S1LC into the transfected HEK-293T and ARPE-19 cells. mRNA-S1LC consisted of all the structural components and its ORF encoded for the nsPs and luciferase. The luciferase expression was evaluated by its functional activity and western blot. Our study confirms that the SAM-based mRNA-S1LC was present inside the cell and was able to express the protein for the tested duration; however, it was not able to reverse transcribe and integrate into the genomic DNA of the transfected cells.

The final section of the study was designed for the future refinement of the SAM-based mRNA for various modalities. Herein, *in silico* 3D structure for each nsP variant was generated and analyzed. These 3D structures will be validated in our future crystallographic studies using the *in vitro* expressed proteins. We also designed, cloned, and expressed different nsPs in BL21 (λDE3) cells using the T7 expression system for our future crystallographic studies.

The research confirms the longer expression of the antigenic protein and the genetic stability of the SAM-platform. A limitation of the study was the absence of non-amplifying mRNA as a control. Further, this study does not provide the direct evidence of the minus strand generated for the SAM but confirms the functional attributes.

Recently, we have confirmed the minus strand of mRNA in a cell-based assay using qPCR as described below.

<u>Methods</u>

The oligonucleotides (ML462, ML463, ML464, ML465, ML466, ML467, ML468, ML469 and ML470) were designed to specifically binds and amplify the first strand DNA from the minus-strand sequence of the mRNA. These oligonucleotides were synthesized from Sigma Aldrich and sequences are mentioned in **Supplementary Table 13**. mRNA-628.2 used for GEMCOVAC-OM was used for transfecting the HEK-293T cells and expression was quantified by flow cytometry as described previously. The spike-protein expression was monitored for 48 hours and 72 hrs. Further, to verify the minus-strand, the total RNA was isolated using the PureLink™ RNA Mini kit as per the manufacturer protocols (Thermo

Scientific). Next, the first-strand DNA encoding the spike antigenic region was synthesized using the ML462 alone, combination of ML462 and ML465, or pool of all the nine-oligonucleotides using SuperScript™ II Reverse Transcriptase (Thermo Scientific). The amplified strand (50 ng) was later used into the qPCR using SsoFast EvaGreen Supermix (Bio-Rad) on QuantStudio 5 Real time PCR system (Applied Biosystems). The qPCR amplification was carried out using forward and reverse primers (ML470 and ML479). Linearized plasmid DNA of mRNA-628.2 and first-strand from the total RNA of luciferase encoding self-amplifying mRNA (mRNA-S1LC) transfected HEK-293T cells were used as control samples.

Results

The mRNA-628.2 encoding the spike protein was transfected in HEK-293T cells and the expression was monitored. The flow analysis showed that 59 ± 5 % of total HEK-293T cells were positive for anti-spike IgG staining at 48 hours post transfection and 23 ± 0.4 % at 72 hrs. Next, transfected cells were harvested, total RNA was isolated, and used for first-strand generation. The minus-strand was confirmed though the qPCR method wherein we specifically generated the first-strand DNA using the precisely designed oligonucleotides that were targeted to the minus-strand of the spike encoding mRNA only. The oligonucleotides ML470 and ML479 were used for the qPCR that generated the product of 228 base pairs and cycle threshold (Ct) is compiled into **Supplementary Table 14**. The qPCR of first-strand DNA amplified from the RNA of mRNA-628.2 transfected HEK-293T cells generated the cycle threshold from 21-28 for all the tested time points and primer combinations. However, there was no amplification found in the case of negative control wherein mRNA-S1LC was used for the transfection. This data confirms the generation of minus-strand of the mRNA-628.2 due to its self-amplifying nature.

Supplementary Table 13. Oligonucleotides designed to detect the (-) strand of mRNA.

Primer name	Primer sequence (5'---3')	Size (bp)
ML462	TGTTCGTGTTTCTGGTGC	18
ML463	TCCTGGACCACAAAAACAAC	20
ML464	CGTGGAAAAGGGCATCTAC	19
ML465	CTTCAACTGCTACTTCCCAC	20
ML466	CTCAGAGCATCATTGCCTAC	20
ML467	ACCAGCGGATGGACATTTG	19
ML468	ATCAGAGCCGCCGAGATTAGAG	22
ML469	TAAGAACCACACAAGCCCC	19
ML470	CAAGAATCTGAACGAGAGCC	20

Supplementary Table 14. qPCR data of mRNA transfected cells.

Cell line	mRNA	Post-transfection (Hrs)	First-strand oligonucleotides	Cycle threshold (Ct)
HEK-293T	GEMCOVAC-OM	48	ML462	21.2
		48	ML462 + ML465	21.9
		48	Pool of 9 oligos	28.4
		72	ML462	27.3
		72	ML462 + ML465	23.1
		72	Pool of 9 oligos	23.4
	Luciferase	48	ML462	Not detected
		48	ML462 + ML465	Not detected
		48	Pool of 9 oligos	Not detected
		72	ML462	Not detected
		72	ML462 + ML465	Not detected
		72	Pool of 9 oligos	Not detected
Linearized plasmid DNA (50 ng)		-		6.9

4. Methods used for the characterization of the GEMCOVAC-OM.

Below are the tables listing all the methods that are used for the release of our mRNA drug substance (only mRNA) and mRNA drug product (mRNA complexed with the carrier) from the cGMP production.

Supplementary Table 15. mRNA drug substance characterization

Sr. No.	Test	Acceptance Criteria	Result
1	Appearance by visual inspection	Should be clear and colourless solution, free from any particle	Clear and colourless solution, free from any particle
2	pH	6.2-6.8	6.42
3	mRNA concentration by Fluroscence assay	NLT 1.0mg/mL	1.28 mg/mL
4	mRNA purity by UV-spectrophotometry.Absorbance at 260/absorbance at 280 nm	Not less than 1.8	2.16
5	mRNA purity by UV-spectrophotometry.Absorbance at 260/absorbance at 230nm	Not less than 1.9	2.55
6	Identity by EtBr stained agarose gel electrophoresis	Single band should be observed above 9kb marker band	Single band observed above 9kb marker band
7	mRNA purity by SE-HPLC	Not less than 90%	100%
8	Residual RNase content by enzymatic method	Not more than 0.083pg/µL	Less than 0.083 pg/µL
9	Residual protein by silver stained SDS-PAGE	No band should be observed	Band is not observed
10	Plasmid DNA contamination by RT-PCR	Should be less than 10ng/µg mRNA	0.0021 ng/10µg mRNA
11	Bacterial endotoxin	Should be less than 200 EU/mL	Less than 100 EU/mL
12	Bioburden	Should be less than 1 CFU/mL	00 CFU/mL
13	Sequencing of spike protein coding region of mRNA	Should be 100% match with reference	100% match with reference

Supplementary Table 16. mRNA drug product characterization.

Sr. No	Test	Acceptance Criteria	Result
1	Appearance of cake	Should be off white amorphous intact cake with uniform appearance	Complies
2	Appearance after reconstitution	Should be off white translucent liquid free from any visible particles	Complies
3	Reconstitution Time	Not more than 180 seconds	55 seconds
4	pH	6.0 ± 0.5	6.12
5	Particle size	100 nm - 200 nm	138.77
6	Polydispersity index	0.2-0.5	0.3809
7	RNA integrity in complex by agarose gel electrophoresis	Free RNA should not be detected in the gel below the loading well for non-extracted sample and the size of RNA should be above 9 kb marker in extracted sample; should be consistent with internal reference standard	Complies
8	RNase protection assay by agarose gel electrophoresis	The size of extracted RNA from RNase treated sample and the extracted RNA from untreated sample should be above 9kb marker	Complies
9	Extracted mRNA recovery by fluorescence	90 - 120 µg/mL	94.16 µg/mL
10	DOTAP content by RP-HPLC	2.4 to 3.6 mg/mL	2.82 mg/mL
11	Squalene content by RP-HPLC	2.9 to 4.5 mg/mL	3.34mg/mL
12	Osmolality	400 - 500 mOsm/kg	460 mOsm/kg
13	Sucrose Content	80-120 mg/mL	107.65 mg/mL
14	Spike protein specific IgG titre	NLT 80% seroconversion (8 out of 10 mice)	100% seroconversion (10 out of 10 mice)
15	Container closure integrity test	None of the container should fail in the leak test	Complies

16	Uniformity of weight	Check and record	0.128 gm
17	Extractable volume	NLT 0.5 mL	0.6 mL
18	Moisture content	NMT 5%	1.24%
19	Particulate matter	≥ 10µm diameter: NMT 6000/container, ≥ 25µm diameter: NMT 600/container	≥ 10µm diameter: 21/container. ≥ 25µm diameter: 01/container
20	Bacterial Endotoxin	< 100 EU/mL	< 50 EU/mL
21	Sterility	No growth should be observed on FTGM and SCDM	Complies

Methods used for Vaccine Production

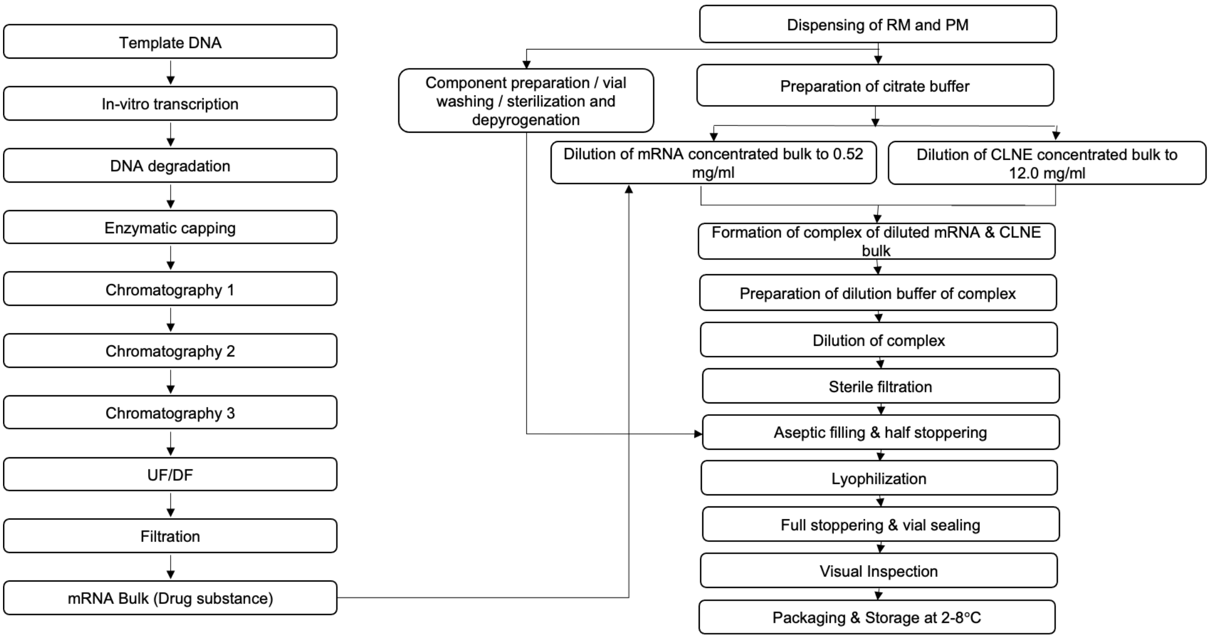

Supplementary Figure 12. Manufacturing process of GEMCOVAC-OM
