# Supplementary material for: An Omicron-specific, self-amplifying mRNA booster vaccine for COVID-19: a phase 2/3 randomized trial
Source: Nat Med. 2024 Apr 18;30(5):1363–72. doi: 10.1038/s41591-024-02955-2 (PMC11108772; doi:10.1038/s41591-024-02955-2)
Supplement: Supplementary file 2 — Reporting Summary [file 41591_2024_2955_MOESM2_ESM.pdf]

nature portfolio

Corresponding author(s): Sanjay Singh

Last updated by author(s): 20/03/2024

Reporting Summary

Nature Portfolio wishes to improve the reproducibility of the work that we publish. This form provides structure for consistency and transparency in reporting. For further information on Nature Portfolio policies, see our Editorial Policies and the Editorial Policy Checklist.

Statistics

For all statistical analyses, confirm that the following items are present in the figure legend, table legend, main text, or Methods section.

n/a	Confirmed	
☐	☒	The exact sample size (*n*) for each experimental group/condition, given as a discrete number and unit of measurement
☐	☒	A statement on whether measurements were taken from distinct samples or whether the same sample was measured repeatedly
☐	☒	The statistical test(s) used AND whether they are one- or two-sided<br>*Only common tests should be described solely by name; describe more complex techniques in the Methods section.*
☐	☒	A description of all covariates tested
☐	☒	A description of any assumptions or corrections, such as tests of normality and adjustment for multiple comparisons
☐	☒	A full description of the statistical parameters including central tendency (e.g. means) or other basic estimates (e.g. regression coefficient) AND variation (e.g. standard deviation) or associated estimates of uncertainty (e.g. confidence intervals)
☐	☒	For null hypothesis testing, the test statistic (e.g. *F*, *t*, *r*) with confidence intervals, effect sizes, degrees of freedom and *P* value noted<br>*Give P values as exact values whenever suitable.*
☒	☐	For Bayesian analysis, information on the choice of priors and Markov chain Monte Carlo settings
☒	☐	For hierarchical and complex designs, identification of the appropriate level for tests and full reporting of outcomes
☒	☐	Estimates of effect sizes (e.g. Cohen's *d*, Pearson's *r*), indicating how they were calculated

Our web collection on statistics for biologists contains articles on many of the points above.

Software and code

Policy information about availability of computer code

Data collection	Clinion Version 3.1
Data analysis	SAS® Version 9.4 (SAS Institute Inc, Cary, North Carolina), GraphPad Prism (Version 9.5.1), corrplot package (Version 0.92) within Rstudio (Version 2022.12.0.0), FlowJo software version 10.8.1

For manuscripts utilizing custom algorithms or software that are central to the research but not yet described in published literature, software must be made available to editors and reviewers. We strongly encourage code deposition in a community repository (e.g. GitHub). See the Nature Portfolio guidelines for submitting code & software for further information.

Data

Policy information about availability of data

All manuscripts must include a data availability statement. This statement should provide the following information, where applicable:

- Accession codes, unique identifiers, or web links for publicly available datasets
- A description of any restrictions on data availability
- For clinical datasets or third party data, please ensure that the statement adheres to our policy

Individual participant data will be made available for meta-analysis when the trial is complete. The request should be approved by an Ethics Committee or the Institutional Review Board of the institution to which the person requesting the information belongs. If approved, it should be directed to the corresponding author at sanjay.singh@gennova.co.in. The requester will need to sign a data access agreement. Data will be shared through a secure online platform within 2 months of

the data access agreement. The aggregated data is included in this manuscript.
DDBJ datasets were used to design GEMCOVAC-19 (Accession no. - LC776732.1) and GEMCOVAC-OM (Accession no. - LC769018).

Research involving human participants, their data, or biological material

Policy information about studies with human participants or human data. See also policy information about sex, gender (identity/presentation), and sexual orientation and race, ethnicity and racism.

Reporting on sex and gender	In this clinical trial, gender was recorded based on self-reporting of the participant. The concept of sex and gender being separate is not widely accepted in India and asking such questions could be regarded as offensive. Hence, we did not actively record the sex and gender separately. However, if a participant were to actively report a difference in the sex and gender, we would have recorded it. In our study, none of the participants reported differences in their sex and gender.
Reporting on race, ethnicity, or other socially relevant groupings	The study was conducted in India. All the participants were of Indian ethnicity.
Population characteristics	The mean age of subjects in the GEMCOVAC-OM and COVISHIELD groups was comparable (33.8 and 32.5 years, respectively). Male subjects in the COVISHIELD arm (79.7%) were slightly higher as compared to the GEMCOVAC-OM arm (68.2%). There was no significant difference in the mean weight, height and BMI in participants receiving GEMCOVAC-OM (62.6 kg, 163.1 cm and 23.6 kg/m2) and COVISHIELD (63.9 kg, 163.9 cm and 23.8 kg/m2).<br>Total 29 subjects (GEMCOVAC-OM [n = 27] and COVISHIELD [n = 2]) reported at least one medical history. Surgical And Medical Procedures (GEMCOVAC-OM [n = 15] and COVISHIELD [n=1]) and vascular disorders (GEMCOVAC-OM [n = 7] and COVISHIELD [n=0]) were most commonly observed.
Recruitment	Subjects who meet the inclusion/exclusion criteria and have successfully completed all screening procedures were randomized in the study by using interactive web response system (IWRS) method into two arms. Since this was digitized, changes of bias in recruitment are low.
Ethics oversight	The clinical trial protocol was approved by the Central Drugs Standard Control Organization (CDSCO) and the local independent Ethics Committee for each clinical trial site. The list of Ethics Committee of each site is provided in the Supplementary Information Table 1. A Data Safety Monitoring Board oversaw the safety of the study.

Note that full information on the approval of the study protocol must also be provided in the manuscript.

Field-specific reporting

Please select the one below that is the best fit for your research. If you are not sure, read the appropriate sections before making your selection.

☒ Life sciences ☐ Behavioural & social sciences ☐ Ecological, evolutionary & environmental sciences

For a reference copy of the document with all sections, see nature.com/documents/nr-reporting-summary-flat.pdf

Life sciences study design

All studies must disclose on these points even when the disclosure is negative.

Sample size	Phase 3 consisted of a safety and an immunogenicity cohort. The safety cohort consisted of 3140 participants of which 3000 were included in the GEMCOVAC-OM arm. The immunogenicity cohort was analyzed for two primary endpoints based on WHO guidelines, with individuals randomized to GEMCOVAC-OM and COVISHIELD in a 2:1 ratio. A sample size of 420 (280 in GEMCOVAC-OM and 140 in COVISHIELD) was found adequate for assessing non-inferiority of neutralizing antibody titers if the lower limit of the two sided 95% CI of the LSGMR (GMT of GEMCOVAC-OM / GMT of COVISHIELD) was > 0.67 considering a standard deviation of 1.82, alpha error of 5%, power of 90% and a dropout rate of 20%. A sample size of 381 (254 in GEMCOVAC-OM and 127 in COVISHEILD) was found adequate for assessing the non-inferiority of seroconversion difference considering a margin of -10%, alpha error of 5%, power of 90% and a 20% drop out rate. The sample size of 420 (280 in GEMCOVAC-OM and 140 in COVISHIELD arm) was considered in this study to provide adequate numbers for the statistical analysis of both the primary endpoints.
Data exclusions	There were 14 participants who were excluded from the safety and immunogenicity analysis due to a major protocol deviation.
Replication	This was a Phase 2/3 confirmatory study based on which GEMCOVAC-OM received Emergency Use Authorization. There was no requirement of replicating this study.
Randomization	Subjects who met the inclusion criteria and successfully completed all screening procedures were randomized in the study by using interactive web response system (IWRS). Unique randomization codes were assigned to the subjects and remained unchanged until the completion of the trial. The randomization codes were generated through Proc Plan using SAS® version 9.4 or higher (SAS Institute Inc, Cary, North Carolina) by an independent biostatistician. Final randomization list was filed securely by the independent biostatistician and accessible to authorized persons only. Participants were enrolled by investigators with the help of the IWRS.<br>In phase 3, 420 subjects in the immunogenicity cohort were randomized in 2:1 ratio through stratified block randomization by using IWRS into GEMCOVAC-OM and COVISHIELD arms respectively. A randomisation code was assigned to each participant in sequence in the order of enrolment, and then the participants received the investigational products labelled with the same code.
Blinding	The route of administration for GEMCOVAC-OM was different compared to the comparator vaccine GEMCOVAC-19 and COVISHIELD.

Blinding	GEMCOVAC-OM was delivered using a intra-dermal needle-free device called Tropis while GEMOCVAC-19 and COVISHIELD was administered using a needle and syringe. Since the routes were visually very different, it was not possible to blind the subject and the staff.

Reporting for specific materials, systems and methods

We require information from authors about some types of materials, experimental systems and methods used in many studies. Here, indicate whether each material, system or method listed is relevant to your study. If you are not sure if a list item applies to your research, read the appropriate section before selecting a response.

Materials & experimental systems

n/a	Involved in the study
☐	☒ Antibodies
☐	☒ Eukaryotic cell lines
☒	☐ Palaeontology and archaeology
☒	☐ Animals and other organisms
☐	☒ Clinical data
☒	☐ Dual use research of concern
☒	☐ Plants

Methods

n/a	Involved in the study
☒	☐ ChIP-seq
☐	☒ Flow cytometry
☒	☐ MRI-based neuroimaging

Antibodies

Antibodies used	CD3 PE-Cy7 (BD 557851, clone SK7, 1:20),<br>CD4 BV480 (BD 566104, clone SK3, 1:20),<br>CD8 FITC (BD 555366, clone RPA-T8, 1:5),<br>IFNg PE (BD 559327, clone B27, 1:5),<br>TNFa APC (BD 551384, clone MAb11, 1:5),<br>IL2 BV421 (BD 562914, clone 5344.111, 1:20),<br>IL2 BV786 (BD 564113, clone MP4-25D2, 1:10),<br>IL13 BV711 (BD 564288, clone JES10-5A2, 1:10),<br>CD19 PerCP-Cy5.5 (BD 561295, clone HIB19, 1:20).<br>CD3 BV605 (BD 563219, clone SK7, 1:20),<br>CD19 PerCP-Cy5.5 (BD 561295, clone HIB19, 1:20),<br>CD20 APC-H7 (BD 560734, clone 2H7, 1:20).<br>Anti-Human IgG (Merck A0170, 1:5000)
Validation	All the antibodies were used as per manufactures specifications. Anti-CD28/49d (FastImmune) from BD biosciences was used in 1 micro-gram per mL concentration as reported earlier (PMID: 31110348).

Eukaryotic cell lines

Policy information about cell lines and Sex and Gender in Research

Cell line source(s)	Vero CCL81 from ATCC
Authentication	Authentication for Vero CCL-81 done by ATCC and grown/passaged according to ATCC recommended protocols. Vero CCL-81 cell lines, lot ID 63803287 was used in this study. According to ATCC certificate of analysis, total cell counts and post-freezing viability were tested using trypan blue based method. Cell growth properties and morphology were tested by visual observation. The sterility test was conducted using the BacT/ALERT 3D system, employing both the iAST bottle (for aerobic conditions) and the iNST bottle (for anaerobic conditions) methods at 32°C temperature. Mycoplasma contamination was determined using Hoechst DNA stain (indirect method), agar culture (direct method), and a PCR-based assay.
Mycoplasma contamination	Vero CCL-81 cells were negative for mycoplasma as confirmed by ATCC and later by PCR testing at IRSHA facility. Vero CCL-81 working cell cultures are screened for detection of mycoplasma contamination by PCR. The method is based on amplification of 16S rRNA genomic DNA target sequence ~270bp in size. Myco-F primer (GGGAGCAAACAGGATTAGATA) and Myco-R primer TGCACCATCTGTCACTCTGTTT) were used in PCR testing.
Commonly misidentified lines<br>(See ICLAC register)	This study did not include any misidentified cell lines.

Clinical data

Policy information about clinical studies

All manuscripts should comply with the ICMJE guidelines for publication of clinical research and a completed CONSORT checklist must be included with all submissions.

Clinical trial registration	Clinical Trial Registry India, CTRI/2022/10/046475.

Study protocol

The protocol has been provided as supplementary material

Data collection

Healthy volunteers were enrolled in 20 hospitals across India between 18th October 2022 and 24th November 2022. Initially, safety and immunogenicity data upto day 29 post-vaccination was collected and analyzed as per the planned interim analysis. Later, clinical data was conducted upto 6 months from the booster vaccination via electronic capture. Blood was collected at various time points during the follow-up till day 90 post after the booster vaccination. Blood was analysed for immunogenicity in respective labs and then transfered to the statistician for analysis.

Outcomes

In phase 2, the primary endpoint were to compare the safety and anti-spike IgG antibodies between the two vaccinated arms at day 29. Secondary endpoints included comparison of seroconversion as assessed by ≥ 2- fold rise in anti-spike IgG antibody titers from baseline, percent neutralization by a surrogate neutralization (cPass™) assay and cellular immune responses at day 29. Exploratory endpoints included comparison of anti-spike IgG antibodies, percent neutralization by cPass assay and cellular immune responses at day 90.
In phase 3, the primary endpoint was the demonstration of non-inferiority of neutralizing antibody GMT assessed by a plaque reduction neutralization test (PRNT50) assay in terms of LSGMR at day 29 and difference in seroconversion (≥ 2-fold rise in antibody titers at day 29 from baseline) between GEMCOVAC-OM and ChAdOx1 nCoV-19. Secondary endpoints included comparison of safety, LSGMR and seroconversion in terms of anti-spike IgG antibody titers, percent neutralization by a surrogate virus neutralization assay (cPass™ assay, GenScript) and cell mediated immunity assessment by intracellular cytokine expression at day 29. Exploratory endpoints included humoral and cellular immune response assessment at day 90.
Differences in the anti-spike IgG and neutralization titers (PRNT) were assessed by LSGMR using ANCOVA test of non-inferiority where baseline titers were used as covariates. Seroconversion rate difference was calculated using the calculated using the Miettinen-Nurminen method. Mean change in surrogate virus neutralization from baseline in the two arms was compared using ANCOVA. Differences in cellular responses was compared by t-test or a Wilcoxon's test based on normality.

Flow Cytometry

Plots

Confirm that:

☒ The axis labels state the marker and fluorochrome used (e.g. CD4-FITC).

☒ The axis scales are clearly visible. Include numbers along axes only for bottom left plot of group (a 'group' is an analysis of identical markers).

☒ All plots are contour plots with outliers or pseudocolor plots.

☒ A numerical value for number of cells or percentage (with statistics) is provided.

Methodology

Sample preparation

Peripheral blood mononuclear cells (PBMCs) were isolated using BD Vacutainer® CPT™ (Cell Preparation Tube) with sodium citrate tubes following the manufacturer's guidelines. Surface and Intracellular cytokine staining (ICS) was performed to measure T-cell effector responses in the splenocytes stimulated with 15-mer peptide pool of Omicron B.1.1.529/BA.1 (spike glycoprotein) peptides viz. PepTivator® SARS-CoV-2 Prot_S B.1.1.529/BA.1. To measure variant spike-protein specific B-cell population, 0.5*106 splenocytes were first labelled with biotinylated Omicron B.1.1529 variant-spike tetramer (Miltenyi Biotech, Germany) followed by surface staining with common B-cell surface marker

Instrument

FACSLyric™ system (3 Laser, 12 Color system)

Software

FlowJo software version 10.8.1 was used for data analysis

Cell population abundance

Cell stock was prepared considering the viable cell population. Cell viability was assessed by trypan blue based method. Non-viable cells were removed based on the cell size and granularity characteristics as indicated by the FSC/SSC plot.

Gating strategy

Lymphocyte cluster was gated using FSC-A/SSC-A plot followed by singlet selection using FSC-A/FSC-H plot. Detailed gating strategies was also shown as extended data figuredin this manuscript.

☒ Tick this box to confirm that a figure exemplifying the gating strategy is provided in the Supplementary Information.
