# Supplementary material for: An Omicron-specific, self-amplifying mRNA booster vaccine for COVID-19: a phase 2/3 randomized trial
Source: Nat Med. 2024 Apr 18;30(5):1363–72. doi: 10.1038/s41591-024-02955-2 (PMC11108772; doi:10.1038/s41591-024-02955-2)
Supplement: Supplementary file 3 — Phase 2 and 3 clinical trial protocol. [file 41591_2024_2955_MOESM3_ESM.pdf]

Supplementary Information: Protocol for "An omicron-specific, self-amplifying mRNA booster vaccine for COVID-19: a phase 2/3 randomized trial."

Title Page

Clinical Study Protocol

GBL/GEMCOVAC-OM/2022/02

**Study Title**	A Prospective, Multi-centre, Open-labelled, Randomized, Phase II study seamlessly followed by a Phase III study to evaluate the Safety, Tolerability and Immunogenicity of GEMCOVAC-OM as a booster in Subjects 18 years of age and older
**Study Vaccine**	GEMCOVAC-OM
**Study Phase**	Phase II / III
**Protocol Number**	GBL/GEMCOVAC-OM/2022/02
**Effective Date**	03/10/2022
**Version**	4.0
**Previous version and date**	V 3.0, 29 Sept 2022
**Sponsor**	Gennova Biopharmaceuticals Ltd.
**Name of Sponsor**	Dr Sanjay Singh<br>CEO, Gennova Biopharmaceuticals Limited

Confidentiality Notice

This document contains confidential information of Gennova Biopharmaceuticals Ltd. This document must not be disclosed to anyone other than the study staff and members of the Independent Ethics Committee/Institutional Review Board and Competent authorities. The information in this document cannot be used for any purpose other than the conduct or evaluation of the clinical investigation without the prior written consent of Gennova Biopharmaceuticals Ltd.

APPROVAL OF PROTOCOL

Name: Dr Sanjay Singh
Designation: Chief Executive Officer
Gennova Biopharmaceuticals Ltd
BTS-2 Building, Chrysalis Enclave, Block-2,
Plot-2, International Biotech Park, Phase II,
MIDC Hinjawadi, Pune-411057,
Maharashtra, India

Name	Signature	Date

Sponsor Representative

Name: Dr Amit Saraf
Gennova Biopharmaceuticals Ltd
Pune-411057, Maharashtra, India
Email: amit.saraf@gennova.co.in
Phone: 020-35250010

Study Administrative Structure

**Sponsor and Study Vaccine Developer/ Manufacturer**	Gennova Biopharmaceuticals Ltd<br>BTS-2 Building, Chrysalis Enclave, Block-2,<br>Plot-2, International Biotech Park, Phase II,<br>MIDC Hinjawadi, Pune-411057,<br>Maharashtra, India
**Sponsor Medical Expert**	Dr. Rohan Gurjar, MBBS, MSc (Pharmacology), PhD (Pharmacology)
**Study Investigators / Clinical Trial Sites**	Phase II part of the study will be conducted at approximately 8 sites and Phase III part of the study will be conducted at 20-25 sites across India
**Clinical Research Organization (CRO) responsible for study conduct**	JSS Medical Research Asia Pacific Private Limited<br>Tower 2, 1st Floor, South Wing, L&T Business Park,<br>Plot no 12/4, Sector 27 D,<br>Delhi Mathura Road, Near Sarai Khawaja Metro Station, Faridabad -121003, Haryana, India
**Immunology Laboratory**	BSL-3 facility, IRSHA,<br>Bharati Vidyapeeth University,<br>D-302, Medical College Rd, Shriram Nagar,<br>Dhankawadi, Pune, Maharashtra 411043<br><br>National Institute of Virology,<br>MCC 130/1, Sus Road, Pashan, Pune, Maharashtra, 411021<br><br>Gennova VFC, BTS-2 Building, Chrysalis Enclave, Block-2, International Biotech Park, Phase II, MIDC Hinjawadi, Pune-411057, Maharashtra. India
**Investigational Product Packaging and Labelling**	Seveillar Clinical Supplies Services Pvt Ltd, Plot: D-219, Ranjangaon MIDC, Shirur, Pune 412220, India

**Clinical Study protocol prepared by:**	
Operations	Name: **Dr. Jayashri Krishnan**<br>Director, Operations<br>Address: JSS Medical Research Asia Pacific Private Limited<br>Tower 2, 1st Floor, South Wing, L&T Business Park,<br>Plot no 12/4, Sector 27 D,<br>Delhi Mathura Road, Near Sarai Khawaja Metro Station,<br>Faridabad -121003, Haryana, India<br><br>Date and Signature:
Medical Writing	Name: **Dr. Ashima Nagpal**<br>Senior Medical Writer<br>Address: JSS Medical Research Asia Pacific Private Limited<br>Tower 2, 1st Floor, South Wing, L&T Business Park,<br>Plot no 12/4, Sector 27 D,<br>Delhi Mathura Road, Near Sarai Khawaja Metro Station,<br>Faridabad -121003, Haryana, India<br><br>Date and Signature:
Biostatistician	Name: **Dr. Lakshman Rao**<br>Manager-SAS & Biostatistics<br>Address: JSS Medical Research Asia Pacific Private Limited<br>Tower 2, 1st Floor, South Wing, L&T Business Park,<br>Plot no 12/4, Sector 27 D,<br>Delhi Mathura Road, Near Sarai Khawaja Metro Station,<br>Faridabad -121003, Haryana, India<br><br>Date and Signature:

AGREEMENT ON THE PROTOCOL

I, the undersigned, as Investigator for this study, have read the foregoing protocol, entitled "A Prospective, Multi-centre, Open-labelled, Randomized, Phase II study seamlessly followed by a Phase III study to evaluate the Safety, Tolerability and Immunogenicity of GEMCOVAC-OM as a booster in Subjects 18 years of age and older" agree to conduct the study as outlined herein and in accordance with all applicable requirements of the country where the study is being conducted, the country where the study will be submitted, and the Sponsor's requirements. Applicable guidelines and regulations include, but are not limited to:

1. Permission to allow the Sponsor and/or its agent or regulatory agencies to inspect study facilities and pertinent records at reasonable times and in a reasonable manner that ensures subject's confidentiality.
2. Immediate notification of the Sponsor and/or its agent of any regulatory inspection related to this study.
3. Submission of the proposed clinical investigation, including the protocol and consent form, to a duly constituted IRB/Ethics Committee for approval, and acquisition of written approval for each, prior to study initiation.
4. Use of IRB/Ethics Committee-approved written informed consent that is obtained prior to study initiation for each subject.
5. Submission of any proposed change in or deviation from the protocol to the IRB/Ethics Committee using a signed formal amendment document prepared by the Sponsor and/or its agent. Any proposed change(s) or deviation(s) from the protocol require that the informed consent also reflect such change(s) or deviation(s), and that the revised informed consent be approved by the IRB/Ethics Committee.
6. Documentation and explanation of the individual protocol deviations on the appropriate CRF page or other Sponsor-approved document.
7. Submission of written reports of serious or unusual adverse events to the Sponsor as defined in the protocol.
8. The Principal Investigator will individually assess the impact of all protocol deviations and adverse events on the conduct of the study, subject's safety, and the suitability of the clinical data and provide to the Sponsor a summary statement.

Signature (Investigator)

……………………………………………………………………………………………….

Print name

………………………………………………………………………………………………

Title

……………………………………………………………………………………………….

Contact Details

……………………………………………………………………………………………….

Date: 03/10/2022

TABLE OF CONTENTS

APPROVAL OF PROTOCOL **3**

AGREEMENT ON THE PROTOCOL **6**

TABLE OF CONTENTS **7**

1 INTRODUCTION **25**

1.1 Background **25**

1.2 Study Product **26**

1.3 Preclinical Data **28**

1.4 Clinical Data till Date **29**

1.4.1 Study Design Justification 29

1.4.2 Risk-Benefit Ratio 30

2 STUDY OBJECTIVES AND ENDPOINTS **32**

3 STUDY DESIGN **35**

3.1 General Design **35**

3.1.1 Interim Analysis 37

3.1.2 Duration of Study 37

3.2 Early Termination of the Study **38**

3.3 Study Procedures **42**

3.3.1 Visit 1 Screening/ Baseline and Randomization Visit (Day 1) 42

3.3.2 Telephonic Visit (Safety) ([Day 7+3] 43

3.3.3 In-person visit ([Safety & Immunogenicity] [Visit 2-Day 29+7]/ [Visit 3-Day 90+14]/ [End of Study (Visit 4-Day 180+14)] 43

3.3.4 Telephonic Follow-up/ Consultation 44

3.3.5 Unscheduled Visits 44

3.4 Study Assessments **44**

3.4.1 Demographics 44

3.4.2 Medical/Surgical History and Concomitant Medications 45

3.4.3 Vital Signs 45

3.4.4 Physical Examination 45

3.4.5 Pregnancy Test 45

3.4.6 Immunogenicity Assessment 45

3.4.7 RT-PCR testing for SARS-CoV-2 46

3.4.8 Adverse Events 46

4 SUBJECT SELECTION AND WITHDRAWAL **47**

4.1 Subject Population **47**

4.2 Inclusion Criteria **47**

4.3 Exclusion Criteria **47**

4.4 Recommendations for Subjects **48**

4.5 Randomization **48**

4.6 Early Withdrawal of Subjects from the Study **49**
5 STUDY PRODUCT 50
5.1 Description **50**
5.2 Treatment Regimen **51**
5.3 Method for Assigning Subjects to Treatment Groups **51**
5.4 Preparation and Administration of Study Product **51**
5.5 Study Product Accountability **52**
5.6 Subject Compliance Monitoring **52**
5.7 Prohibited and Concomitant Therapy **53**
5.7.1 Concomitant Medications 53
5.7.2 Prohibited Medications 53
6 STATISTICAL PLAN 53
6.1 Demographic and Baseline Characteristics **54**
6.1.1 Medical/ Surgical History, Concomitant Medication and Other Safety Evaluations 54
6.2 Analysis of Endpoints **54**
6.2.1 Analysis of Primary Endpoints 54
6.2.2 Analysis of Secondary Endpoints 56
6.3 Sample Size Determination **58**
6.4 Subject Populations for Analysis **59**
7 ADVERSE EVENTS AND SERIOUS ADVERSE EVENTS 59
7.1 Definition **59**
7.1.1 Adverse Events 59
7.1.2 Adverse Events of Special Interest 62
7.1.3 Recording of COVID 19 Events 62
7.1.4 Serious Adverse Events 62
7.2 Procedure for Reporting of Occurrence of Serious Adverse Events **62**
7.3 Pregnancy **63**
8 STUDY PRODUCT MANAGEMENT 64
8.1 Packaging **64**
8.2 Blinding of Study Product **64**
8.2.1 Receipt of Study Product Supplies 64
8.2.2 Storage 64
8.2.3 Dispensing of Study Products 65
8.2.4 Return or Destruction of Study Product 65
9 STUDY ADMINISTRATION 65
9.1 Regulatory and Ethical Considerations, Including the Informed Consent Process **66**
9.1.1 Institutional Ethics Committee 66
9.1.2 Informed consent of the subject 66

9.2 Medical Monitoring 66
9.3 Unblinding Procedures 66
9.4 Pause Rules 66
10 DATA HANDLING AND RECORD KEEPING 67
10.1 Confidentiality 67
10.2 Source Documents 67
10.3 Documentation storage 68
10.4 Case Report Forms 68
10.5 Records Retention 69
11 STUDY MONITORING, AUDITING, AND INSPECTING 69
11.1 Study Monitoring Plan 69
11.2 Auditing and Inspecting 70
11.3 Protocol Deviations 70
11.4 Study and Site Closure 70
12 STUDY FINANCES 71
12.1 Funding Source 71
12.2 Conflict of Interest 71
13 REPORTS ON STUDY RESULTS 71
14 PUBLICATION PLAN 71
15 REFERENCES 72
16 APPENDICES 75
APPENDIX 1. COVID-19 SEVERITY AND SYMPTOMS (MOHFW GUIDELINES)[36] 75
APPENDIX 2. VACCINATION ADVERSE EVENTS ASSESSMENT SCALE[38] 75

LIST OF TABLES

Table 1 Schedule of Visits (Phase II & Phase III) 39
Table 2 Total Blood Sample for Assessment of Immunogenicity 46

LIST OF FIGURES

Figure 1 Flowchart of the study. 37

LIST OF ABBREVIATIONS

ADR	Adverse Drug Reaction
AE	Adverse Event
ANCOVA	Analysis of Co-Variance
ARDS	Acute Respiratory Distress Syndrome
ARF	Acute Respiratory Failure
ARVI	Acute Respiratory Viral Infection
BMI	Body Mass Index
CI	Confidence Interval
COVID-19	Coronavirus 2019
CRF	Case Report Form
CRO	Contract Research Organization
CTCAE	Common Terminology Criteria for Adverse Events
eCFR	Electronic Case Report Form
EDC	Electronic Data Capture
ELISA	Enzyme-Linked Immunosorbent Assay
ESR	Erythrocyte Sedimentation Rate
GMT	Geometric Mean Titre
ICF	Informed Consent Form
ICH-GCP	International Council on Harmonization-Good Clinical Practice
IEC	Institutional Ethics Committee
IgG	Immunoglobulin G
IgM	Immunoglobulin M
IWRS	Interactive Web Response System
MedDRA	Medical Dictionary for Regulatory Activities
MERS-CoV	Middle East Respiratory Syndrome Coronavirus
mRNA	Messenger Ribonucleic Acid
NAbs	Neutralizing Antibodies
PBMC	Peripheral Blood Mononuclear Cells
PI	Principal Investigator
PRNT	Plaque Reduction Neutralization Tests

PT	Preferred Term
RNA	Ribonucleic Acid
RT-PCR	Reverse Transcriptase-Polymerase Chain Reaction
SAE	Serious Adverse Event
SAP	Statistical Analysis Plan
SARS	Severe Acute Respiratory Syndrome
SARS-CoV	Severe Acute Respiratory Syndrome Coronavirus
SARS-CoV-2	Severe Acute Respiratory Syndrome Coronavirus-2
SAS	Statistical Analysis System
SD	Standard Deviation
VNA	Virus Neutralizing Antibody
WHO	World Health Organization

STUDY SYNOPSIS

**Title of Study**	A Prospective, Multi-centre, Open-labelled, Randomized, Phase II Study Seamlessly Followed by a Phase III Study to Evaluate the Safety, Tolerability and Immunogenicity of GEMCOVAC-OM as a booster in Subjects 18 Years of Age and Older
**Protocol ID**	GBL/GEMCOVAC-OM/2022/02
**Sponsor**	Gennova Biopharmaceuticals Ltd
**Phase of Study**	Phase II / III
**Brief Summary**	This is a Phase II seamlessly followed by Phase III study to evaluate the immunogenicity and safety of a booster dose of the mRNA vaccine – GEMCOVAC-OM. The study participants will be adult subjects who are fully vaccinated against COVID-19 with either COVAXIN™ or COVISHIELD™ and received last dose of primary vaccination at least 4 months prior to screening. This booster dose of GEMCOVAC-OM is based on the sequence of Omicron variant of SARS-CoV-2. The study will be conducted in two parts: The phase II part will be conducted in 140 subjects while the Phase III part of the study will enrol 3140 subjects.<br><br>Phase II<br><br>Approximately 140 subjects will be randomized, and the enrolment will be competitive.<br><br>The subjects will be randomized in 1:1 ratio into two arms:<br><br>Arm I: 70 Subjects who have received either COVAXIN™ or COVISHIELD™ as primary vaccination (both doses) will receive a booster dose of the mRNA vaccine GEMCOVAC-OM (intradermal).<br><br>Arm II: 70 Subjects who have received either COVAXIN™ or COVISHIELD™ as primary vaccination (both doses) will receive a booster dose of GEMCOVAC-19 (Intramuscular).<br><br>It will be ensured that at least 20% (n ~14) of the participants in each arm with receive COVAXIN™ and COVISHIELD™.<br><br>All the subjects randomized in Arm I will receive 1 dose of GEMCOVAC-OM and those who are randomized in Arm II will receive 1 dose of GEMCOVAC-19 on Day 1.<br><br>An interim analysis will be performed post Day 29.<br><br>Phase III

	Approximately 3140 subjects will be randomized, and the enrolment will be competitive.<br>The subjects will be randomized into two arms:<br>Arm I: 3000 Subjects who have received either COVAXIN™ or COVISHIELD™ as primary vaccination (both doses) will receive a booster dose of GEMCOVAC-OM.<br>Arm II: 140 Subjects who have received COVISHIELD™ as primary vaccination (both doses) will receive a booster dose of COVISHIELD™.<br>In the immunogenicity cohort of Arm I, a minimum of 42 participants (15%) will have received COVAXIN™ and COVISHIELD™ as their primary vaccination. In the safety cohort of Arm I, a minimum of 300 participants (10%) will have receievd receive COVAXIN™ and COVISHIELD™ as their primary vaccination.<br>All the subjects randomized in Arm I will receive 1 dose of GEMCOVAC-OM, subjects randomized in Arm II will receive 1 dose of COVISHIELD™.<br>The immunogenicity subset will include 280 subjects from Arm I and all the subjects from Arm II (n=140)
**Study Vaccine**	**mRNA vaccine –** GEMCOVAC-OM**:**<br>**Form:** Lyophilized powder to be reconstituted with water for injection (WFI) to be given as intra-dermal injection.<br>**Dose:** 0.1 mL (10 µg) on Day 1.<br>**Composition:** GEMCOVAC-OM consists of an *in-vitro* transcribed mRNA encoding for the Spike (S)-protein of the Omicron variant of the SARS-CoV-2 virus and cationic lipid nanonemulsion (CLNE) in a buffer containing 10% sucrose in 10 mM sodium citrate, pH 6.5.<br>**Excipients:** CLNE is composed of DOTAP chloride, Squalene, Sorbitan Monostearate, Polysorbate 80 and Sodium Citrate Dehydrate.
**Reference Therapy**	**Comparator I**<br>**mRNA vaccine** – GEMCOVAC-19:<br>**Form:** Lyophilized powder for **s**olution for injection to be given intra-muscular.<br>**Dose:** 0.5 mL (10 µg) on Day 1.<br>**Composition:** GEMCOVAC-19 formulation consists of an *in-vitro* transcribed mRNA encoding for the S-protein and CLNE in a buffer containing 10% sucrose in 10 mM sodium citrate, pH 6.5.<br>**Excipients:** CLNE is composed of DOTAP chloride, Squalene, Sorbitan Monostearate, Polysorbate 80 and Sodium Citrate Dehydrate.

	**Comparator II:**<br>**Trade Name**: COVISHIELD™ (manufactured by Serum Institute of India Pvt Ltd)<br>**Dose:** 0.5 mL on Day 1<br>**Composition:** It contains ChAdOx1 nCoV- 19 Corona Virus Vaccine (Recombinant) 5×10^{10} viral particles (vp)<br>Recombinant, replication-deficient chimpanzee adenovirus vector encoding the SARS-CoV-2 Spike (S) glycoprotein. Produced in genetically modified human embryonic kidney (HEK) 293 cells.
**Study Type**	Interventional
**Study Design**	Multi-centre, Open-labelled, Randomized, Phase II study seamlessly followed by a Phase III study
**Primary Objective (s)**	Phase II<br>• To assess the safety of adult subjects who received GEMCOVAC-OM as a booster dose till Day 180<br>• To evaluate the immunogenicity as detected by Immunoglobulin G- Enzyme-linked immunosorbent assay (IgG ELISA) against the SARS-CoV-2 Spike protein of GEMCOVAC-OM in comparison with GEMCOVAC-19 at Day 29<br>Phase III<br>• To evaluate the neutralizing antibody (NAb) titers against SARS-CoV-2 post vaccination with GEMCOVAC-OM in comparison with COVISHIELD™ at Day 29
**Secondary Objectives**	Phase II<br>• To evaluate the neutralizing antibody (NAb) against SARS-CoV-2 post vaccination with GEMCOVAC-OM in comparison with GEMCOVAC-19 at Day 29<br>• To evaluate cellular immune response from GEMCOVAC-OM in comparison with GEMCOVAC-19 at Day 29<br>Phase III<br>• To evaluate the immunogenicity as detected by IgG ELISA against the SARS-CoV-2 Spike protein of GEMCOVAC-OM in comparison with COVISHIELD™ at Day 29<br>• To evaluate the neutralizing antibody (NAb) against SARS-CoV-2 post vaccination with GEMCOVAC-OM in comparison with COVISHIELD™ at Day 29<br>• To evaluate the cellular immune response from GEMCOVAC-OM in comparison with COVISHIELD™ at Day 29<br>• To assess the safety of adult subjects who received

	GEMCOVAC-OM as booster dose till Day 180
**Exploratory Objectives**	Phase II<br>• To evaluate the immunogenicity detected by IgG ELISA against the SARS-CoV-2 Spike protein of GEMCOVAC-OM at Day 90<br>• To evaluate the NAb against SARS-CoV-2 post vaccination with GEMCOVAC-OM at Day 90<br>• To evaluate the cellular immune response from GEMCOVAC-OM at Day 90<br>• To assess symptomatic COVID-19 events till end of the study<br>Phase III<br>• To evaluate the immunogenicity detected by IgG ELISA against the SARS-CoV-2 Spike protein of GEMCOVAC-OM at Day 90<br>• To evaluate the NAb titers against SARS-CoV-2 post vaccination with GEMCOVAC-OM at Day 90<br>• To evaluate the cellular immune response GEMCOVAC-OM at Day 90<br>• To assess symptomatic COVID-19 events till end of the study
**Sample size calculation**	No formal sample size has been calculated for Phase II. The 7 day safety post booster dose will be analyzed and presented to the DSMB. After their favourable opinion, the Phase III will commence.<br>The Phase III study consists of safety and the immunogenicity cohort. The safety cohort will consist of 3140 participants of which 3000 will receive GEMCOVAC-OM. This was calculated to ensure that a safety database of 3000 participants who received GEMCOVAC-OM is available.<br>The immunogenicity cohort was calculated for the two primary endpoints<br>1. Non-inferiority of neutralizing antibody (PRNT) Geometric Mean Titer (GMT) ratio<br>The immunogenicity cohort was calculated based on the WHO guidelines of non-inferiority defined as lower bound of 95% CI in the neutralizing antibody (PRNT) Geometric Mean Titer (GMT) ratio (GMT in GEMCOVAC-OM / GMT in COVISHIELD™) > 0.67.<br>The sample size will be allocated in to 2:1 ratio between GEMCOVAC-OM and COVISHIELD™ arms and includes 20% dropout rate.

	A sample size of 420 (280 in GEMCOVAC-OM and 140 in COVISHIELD™ arm) in the immunogenicity cohort will provide adequate numbers for the statistical analysis considering a non-inferiority margin of 0.67, standard deviation of 1.82 , alpha error of 5% and power of 90%.<br><br>2. Non-inferiority of difference in seroresponse rate<br><br>The immunogenicity cohort was calculated based on the WHO guideline of non-inferiority defined as lower bound of 95% CI in seroresponse rate difference (Seroconversion Rate GEMCOVAC-OM - Seroconversion Rate COVISHIELD™) >-10%.<br><br>The sample size is allocated in a 2:1 ratio between GEMCOVAC-OM and COVISHIELD™ arms and includes 20% dropout rate.<br><br>A sample size of 381 (254 in GEMCOVAC-OM and 127 in COVISHEILD™) in the immunogenicity cohort will provide adequate numbers for the statistical analysis considering a non-inferiority of -10%, alpha error of 5% and power of 90%.<br><br>The sample size of 420 (280 in GEMCOVAC-OM and 140 in COVISHIELD™ arm) is considered in this study to provide adequate numbers for the statistical analysis of both the primary endpoints.
**Number of Subjects**	140 subjects will be randomized in Phase II part of the study while 3140 subjects will be randomized in Phase III study.
**Inclusion Criteria**	Subjects will be randomized in the study who meet all the following criteria:<br>1. Male and female aged ≥ 18 years<br>2. Subject who had received primary vaccination (both doses completed) with either<br>a. COVAXIN™ OR<br>b. COVISHIELD™<br>Wherein last dose of primary vaccination taken at least 4 months prior to the screening visit<br>3. Subject or their legally acceptable representative (LAR) should be capable and willing to give voluntary written informed consent prior to inclusion in the study<br>4. Inclusion of subjects based on clinical judgment by the investigator<br>5. Subjects who had COVID-19 infection after primary vaccination, should have been asymptomatic or RT-PCR negative for at least 3 months

	6. Consent for using effective methods of contraception during the entire study period<br>7. No medical history of pronounced vaccine-induced reactions or complications after receiving immunobiological products<br>8. No acute infectious and/or respiratory diseases within 14 days prior to screening<br>9. Subjects able to comprehend and comply with study requirements and procedures and willing to complete subject diary
**Exclusion Criteria**	Subjects will be entered into the study only if they meet none of the following criteria:<br>1. Prior receipt of any COVID-19 vaccine in less than 4 months of duration<br>2. Pregnant or lactating mothers<br>3. Any significant illness or any other current or pre-existing health condition (e.g. any major pulmonary, cardiovascular, renal, neurological, metabolic, gastro-intestinal, hepato-biliary, haematological functional abnormality, mental or physical disability, blood dyscrasia, major congenital defects, etc.) which in the opinion of the Investigator may affect the safety of the subject or the study endpoints.<br>4. History of chronic infections in immunocompromised subjects<br>5. History of chronic immune disease, Splenectomy or systemic collagenosis<br>6. Subjects with oncological disease within 5 years prior to inclusion into the study<br>7. History of the human immunodeficiency virus, syphilis, hepatitis B, or C<br>8. Acute Kidney injury or dialysis, had transplant and on immunosuppressive therapy<br>9. Currently receiving or have received (in last 4 weeks) medication intended to prevent COVID-19 except for multi-vitamin supplements<br>10. Receipt of steroids and/or immunoglobulins or other blood products within 30 days prior to randomization<br>11. Tattoos or scars at the injection site, which in the medical opinion of the investigator does not allow assessing the local response to the study vaccine administration

	12. Participation in other interventional clinical trial within the previous 90 days prior to randomization and over duration of the trial<br>13. Any other condition that the study physician considers as a barrier to the trial completion as per the protocol
**Randomization**	**Phase II**<br>Approximately 140 subjects will be randomized, and the enrolment will be competitive.<br>The subjects will be randomized in 1:1 ratio into two arms:<br>Arm I: 70 Subjects who have received either COVAXIN™ or COVISHIELD™ as primary vaccination (both doses) will receive a booster dose of GEMCOVAC-OM.<br>Arm II: 70 Subjects who have received either COVAXIN™ or COVISHIELD™ as primary vaccination (both doses) will receive a booster dose of GEMCOVAC-19.<br>It will be ensured that at least 20% (n ~14) of the participants in each arm with receive COVAXIN™ and COVISHIELD™.<br>**Phase III**<br>Approximately 3140 subjects will be randomized, and the enrolment will be competitive.<br>The subjects will be randomized into two arms:<br>Arm I: 3000 Subjects who have received either COVAXIN™ or COVISHIELD™ as primary vaccination (both doses) will receive a booster dose of GEMCOVAC-OM.<br>Arm II: 140 Subjects who have received COVISHIELD™ as primary vaccination (both doses) will receive a booster dose of COVISHIELD™<br>In the immunogenicity cohort of Arm I, a minimum of 42 participants (15%) will have received COVAXIN™ and COVISHIELD™ as their primary vaccination. In the safety cohort of Arm I, a minimum of 300 participants (10%) will have receieved COVAXIN™ and COVISHIELD™ as their primary vaccination.
**Dosing Schedule**	**Phase II**: All the subjects randomized in Arm I will receive 1 dose of GEMCOVAC-OM, and those who are randomized in Arm II will receive 1 dose of GEMCOVAC-19 vaccine on Visit 1 (Day 1).<br>**Phase III:** All the subjects randomized in Arm I will receive 1 dose of GEMCOVAC-OM and subjects randomized in Arm II will receive 1 dose of COVISHIELD™.
**Treatment Duration and Follow-up period**	Study Vaccine will be administered as a single dose on Day 1. Vaccinated subjects included in the study will be followed up to approximately 6 months (180+ 14 days) of study period thereafter.

Visits and study procedures

Explained in detail in Schedule of procedures Table 1.

All subjects receiving booster vaccine will have 4 on-site visits during the trial period. The subjects will be screened on Visit 1 and then the GEMCOVAC-OM/comparator vaccine will be administered on the same day (Day 1). Subsequently, all subjects included in the study will visit study site for safety and immunogenicity assessments on Visit 2 (Day 29+7) , Visit 3 (Day 90+14) and Visit 4 (Day 180+14) i.e., End of Study visit.

Blood samples will be taken to assess the immunogenicity parameters as listed below:

Immunogenicity parameter	Blood Vol. (mL)	Blood Vol. (mL)	Blood Vol. (mL)
Day	Day 1	Day 29+7	Day 90+14
Humoral Immune Response	5	5	5
Cellular Immunity	16	16	16

[1]Blood sampling will be performed on Day 1 after the subject is randomized to either of the arms. Sampling will be performed immediately prior to the Study vaccine/Reference vaccine administration.

[2]Approximately 65 mL of blood will be drawn from subjects receiving Study vaccine/comparator vaccine during the period of 6 months from Visit 1.

There will be remote/telephonic consultation for all subjects to record their well-being including experience of any adverse events on Day 7.

All subjects will have to complete subject diary up to 7-days post vaccination.

Where necessary, unscheduled visits and/or Telephonic consultation will be performed to evaluate potential COVID-19 as well as to ensure safety and well-being of all trial subjects.

The following safety evaluation will be done. Vitals; physical examination (general and systemic examination) at all onsite visits, and specific symptoms for COVID-19 will be asked at all visits].

In Phase II part of the study, immunogenicity assessments will be performed in all subjects included in Arm I and Arm II as per schedule of events.

	In Phase III part of the study, immunogenicity assessments will be performed in 280 subjects in Arm I, and 140 subjects in Arm II as per schedule of events.
**Criteria for Evaluation**	**Phase II**<br>**Primary Endpoints**<br>• Comparison of anti-Spike (omicron variant) IgG Antibodies (GMT) at Day 29 with GEMCOVAC-OM against GEMCOVAC-19<br>• Occurrence and severity of local and systemic reactogenicity adverse events (AEs) for 7 days following vaccination<br>• Occurrence of unsolicited adverse events up to day 29 post vaccination<br>• Occurrence of related unsolicited adverse events throughout the duration of the study<br>• Occurrence of serious adverse events (SAEs): throughout the duration of the study<br>**Secondary Endpoints**<br>• Comparison of seroconversion rates as assessed by ≥ 2- fold rise in antibody titers at Day 29 from baseline<br>• Comparison of neutralizing antibodies against SARS-CoV-2 using a surrogate virus assay (cPASS™ neutralization antibody kit) at Day 29<br>• Cell mediated immunity assessment by cytokine expression from stimulated PBMCs at Day 29 (20% of participants)<br>**Exploratory Endpoints**<br>• GMT measured by IgG-ELISA against SARS-CoV-2 Spike protein (omicron variant) post booster administration at Day 90<br>• Neutralisation antibodies against SARS-CoV-2 using a surrogate virus assay (cPASS™ neutralization antibody kit) post booster administration at Day 90<br>• Assessment of cellular immune responses from stimulated PBMCs at Day 90 (20% of participants)<br>• Symptomatic laboratory confirmed COVID-19 cases throughout the duration of the study<br><br>**Phase III**<br>**Primary Endpoints**<br>• Comparison of neutralizing antibody titers against SARS-CoV-2 (omicron variant) using plaque reduction neutralization test (PRNT) assay at Day 29 by non-inferiority

	• Comparison of seroconversion rates as assessed by ≥ 2- fold rise in neutralizing antibodies against SARS-CoV-2 (omicron variant) using PRNT at Day 29 between GEMCOVAC-OM and COVISHIELD™ by non-inferiority<br>**Secondary Endpoints**<br>• Comparion of anti-Spike (omicron variant) IgG antibodies (GMT) between GEMCOVAC-OM and COVISHIELD™ at Day 29 by non-inferiority<br>• Comparison of seroconversion rates as assessed by ≥ 2- fold rise in IgG antibody titers at Day 29 between GEMCOVAC-OM and COVISHIELD™ by non-inferiority<br>• Comparison of neutralizing antibodies against SARS-CoV-2 using a surrogate virus assay (cPASS™) at Day 29<br>• Assessment of cellular immune responses from stimulated PBMCs at Day 29 (25% of participants)<br>• Occurrence and severity of local and systemic reactogenicity adverse events (AEs) for 7 days following vaccination<br>• Occurrence of unsolicited adverse events up to day 29 post vaccination<br>• Occurrence of related unsolicited adverse events throughout the duration of the study<br>• Occurrence of serious adverse events (SAEs): throughout the duration of the study<br>**Exploratory Endpoints**<br>• GMT measured by IgG-ELISA against SARS-CoV-2 Spike (omicron variant) protein post booster administration at Day 90<br>• Neutralisation antibodies against SARS-CoV-2 using a surrogate virus assay (cPASS™ neutralization antibody kit) post booster administration at Day 90<br>• GMT of SARS-CoV-2 (omicron variant) specific serum neutralizing antibody levels using live virus (PRNT) assay, at Day 90<br>• Assessment of cellular immune responses from stimulated PBMCs at Day 90 (25% of participants)<br>• Symptomatic laboratory confirmed COVID-19 cases throughout the duration of the study
**Criteria for Evaluation: Safety**	All adverse events, whether previously known or not, will be recorded with their description, intensity, action taken, duration, outcome, and opinion about causal relationship. COVID-19 will not be recorded as AE, however, the subject will be followed up till resolution of COVID-19.
**Statistical Analysis Plan and Data handling/management**	A Statistical Analysis Plan (SAP) will be prepared as a separate document and finalized before database lock. Any deviation from the

	original statistical plan will be described and justified in the final report, as appropriate. The procedure for accounting for missing, unused, and spurious data will be included in the Statistical Analysis Plan. All statistical analysis will be conducted using SAS®, Version 9.4 or higher.<br>Electronic Case Report Forms (eCRFs) will be used to collect information required for statistical analysis. The eCRF will be designed as per protocol, protocol amendment(s). CDM team will set up the study database/application as specified in Data Management Plan. Data will be cleaned through query generation and resolution. Medical coding, handling of external data etc. will be done as per Data Management Plan. Medical coding will be done by using standard medical dictionaries like Med-DRA and WHODD.
**Statistical Methods**	**Statistical Analysis**<br>In general, the variables will be summarized by using standard descriptive statistics. Continuous variables will be summarized with the number (n) of non-missing observations, mean, standard deviation, median, and minimum and maximum, unless otherwise specified. For categorical data, descriptive statistics will be presented with the number and percentage of subjects in the various categories of the endpoint. All the comparative analysis will be considered statistically significant at 5% level of significance unless stated otherwise. Statistical software SAS® version 9.4 or higher (SAS Institute Inc, Cary, North Carolina) will be used for the analyses.<br>**Analysis of Primary Endpoint**<br>**Phase - II**<br>• Comparison of Anti-Spike (omicron variant) IgG Antibodies (GMT) at Day 29 with with GEMCOVAC-OM against GEMCOVAC-19<br>• Occurrence and severity of local and systemic reactogenicity adverse events (AEs) for 7 days following vaccination<br>• Occurrence of unsolicited adverse events up to day 29 post vaccination<br>• Occurrence of related unsolicited adverse events throughout the duration of the study<br>• Occurrence of serious adverse events (SAEs): throughout the duration of the study<br>Geometric mean titres (GMT) will be calculated at day 29 with Omicron-specific vaccine (GEMCOVAC-OM) with GEMCOVAC-19 assessed by non-inferiority along with their GMT Ratio, 2-sided 95% CI and p-value. The GMT Ratio, 95% CI and p-value will be calculated using ANCOVA model.<br>The percentage of subjects reporting each local and systemic reactogenicity adverse events (AEs) for 7 days following

	vaccination, unsolicited related adverse events up to day 180 post vaccination, serious adverse events (SAEs): throughout the duration of the study and adverse events of special interest (AESI): throughout the duration of the study will be tabulated with exact 95% CI.<br><br>**Phase – III**<br>• Comparison of neutralizing antibody titers against SARS-CoV-2 (omicron variant) using plaque reduction neutralization test (PRNT) assay at Day 29 by non-inferiority<br>• Comparison of seroconversion rates as assessed by ≥ 2-fold rise in neutralizing antibodies against SARS-CoV-2 (omicron variant) using PRNT at Day 29 between GEMCOVAC-OM and COVISHIELD™ by non-inferiority<br>Geometric mean titres (GMT) of neutralizing antibody (NAb) titers against the BA.1 omicron specific SARS-CoV-2 will be calculated at baseline and at day 29 in both the treatment groups. The geometric mean titres (GMT) calculation will be performed by taking the anti-log of the mean of the log transformations using Analysis of Covariance (ANCOVA) in which the log transformed value of titres at Day 29 will be included as outcome variable, Treatment group as fixed effect and baseline log transformed titre as covariates. The GMT will be the anti-log value of least square mean obtained from the ANCOVA model and the GMTR will be the ratio of GMTs of the two groups. A non inferiority margin will be considered as 0.67 as per WHO guidelines, i.e. non inferiority is demonstrated if the lower bound of 95% CI GMT ratio (GEMCOVAC-OM/ COVISHIELD™) is > 0.67.<br><br>Seroconversion using neutralizing antibodies (PRNT) against the omicron specific SARS-CoV-2 (≥2- fold rise) will be calculated at Day 29 for both the groups. 95% CI will be calculated for percentage by using Clopper-Pearson method. Difference between percentages and the 95% CI for the difference will also be calculated by using Meitinen-Nurminen method. Chi-square or Fisher's exact test will be used to compare the subjects who achieve ≥2 fold rise between GEMCOVAC-OM and COVISHIELD™ arms.<br>Non-inferiority margin for difference in seroconversion rates is considered as -10% (Seroconversion Rate GEMCOVAC-OM – Seroconversion Rate COVISHIELD™), i.e. GEMCOVAC-OM vaccine will be considered as non-inferior to the COVISHIELD™ vaccine if the lower bound of 95% CI of the difference in Seroconversion Rates is > -10 %.

1 INTRODUCTION

1.1 Background

Coronaviruses are large group of viruses that cause illness in humans and animals. Four human coronaviruses (HCoV 229E, NL63, OC43, and HKU1) had been endemic globally that result in upper respiratory tract infections in adults. According to WHO, the environmental samples taken from Huanan seafood market (Wuhan, China) were tested positive for SARS-CoV-2, but the specific animals associated with the virus have not been identified. Based on previous evidence, the bats, the host of more than 30 coronaviruses, may be the origin of COVID-19. On 11th February 2020, the International Committee on Taxonomy of Viruses renamed the virus as severe acute respiratory syndrome coronavirus-2 (SARS-CoV-2) and WHO announced the epidemic disease caused by SARS-CoV-2 as coronavirus disease 2019 (COVID-19).[1]

Disease Epidemiology

Coronaviruses are the single-stranded positive-sense RNA viruses, including 4 genera: Alphacoronavirus, Betacoronavirus, Gammacoronavirus, and Deltacoronavirus. Alpha- and Betacoronaviruses mainly infect mammals; the rest of two primarily infect birds. Seven coronaviruses that related to human disease had been identified.[1] Current available evidence for COVID-19 suggests that the causative virus (SARS-CoV-2) has a zoonotic source closely related to bat-origin SARS-like coronavirus. The genome sequence of a SARS-like coronavirus in bats uses human Angiotensin-Converting Enzyme 2 (ACE2) as a receptor, thus having replication potentials in human cells.[2]

The main source of infection is through direct person-to-person transmission occurring through close contact, mainly through respiratory droplets that are released when the infected person coughs, sneezes, or talks. The coronaviruses already identified might only be the tip of the iceberg, with potentially more novel and severe zoonotic events to be revealed [2-5]

The median incubation period is 5.1 days (range 2–14 days).[6-8] The precise interval during which an individual with COVID-19 is infectious is uncertain. As per the current evidence, the period of infectivity starts 2 days prior to onset of symptoms and lasts up to 8 days. The extent and role played by pre-clinical/ asymptomatic infections in transmission still remain under investigation. The progression from prodromes (usually fever, fatigue, and cough) to severe pneumonia, acute respiratory distress syndrome (ARDS) requiring oxygen support, mechanical ventilation, or extracorporeal membrane oxygenation (ECMO) and is most commonly seen in the second week following onset of symptoms of a viral infection.[2] The kinetics of viral replication in the respiratory tract has not been well characterized, but this relatively slow progression provides a potential time window and opportunity for antiviral therapies to influence the course of the disease.

Clinical Presentation

Clinical presentations greatly resemble SARS-CoV. The most common laboratory abnormalities observed were decreased total lymphocytes, prolonged prothrombin time, and elevated lactate

dehydrogenase. Compared with non-ICU patients, patients who received ICU (intensive care unit) care had numerous laboratory abnormalities. These abnormalities suggest that SARS-CoV-2 infection may be associated with cellular immune deficiency, coagulation activation, myocardia injury, hepatic injury, and kidney injury. [9-14] Other abnormal laboratory finding included elevations in C-reactive protein (CRP), erythrocyte sedimentation rate (ESR), serum ferritin, and interleukin-6 (IL6).[13] Many patients also had increased levels of D-dimer, lactate dehydrogenase (LDH), creatine kinase (CK), prolonged prothrombin time, alanine aminotransferase (ALT), and aspartate aminotransferase (AST). [9-15] The typical imaging features of chest computed tomography (CT) for novel coronavirus pneumonia (NCP) included ground-glass opacity, bilateral patchy shadows, and subsegmental areas of consolidation, sometimes with a rounded morphology and a peripheral lung distribution.[10,12-14,16-17] Abnormalities in chest X-ray vary, but typically reveal bilateral multi-focal opacities. Imaging may be normal early in infection and can be abnormal in the absence of symptoms.[18]

Management of Person with COVID-19 [19, 20]

Guidelines have been published by various Health Authorities about the management of COVID-19. With WHO as convener, experts who have contributed to the development of vaccines against COVID-19 are now focused on booster doses. The key aim of worldwide Research and Development (R&D) is to extend the immunity which will help to reduce the risk of hospitalization due to COVID-19 infection. Some drugs and vaccines have been accessed through Emergency Use Authorization, expanded access programs, or compassionate use mechanisms.

Preclinical & clinical development of COVID-19 vaccines:[21]

As per the COVID-19 vaccine tracker and landscape dated 9th Sep 2022, around the world, there are now 171 COVID-19 vaccine candidates undergoing clinical trials and 198 candidates in pre-clinical development.

1.2 Study Product

Study Vaccine

Name: GEMCOVAC-OM

Dosage form: Lyophilized powder to be reconstituted with water for injection (WFI) to be given as intra-dermal injection

Composition per dose (0.1 mL):

Active substance: GEMCOVAC-OM formulation consists of an *in vitro* transcribed mRNA encoding for the BA.1 omicron variant of the S-protein and CLNE in a buffer containing 10% sucrose in 10 mM sodium citrate, pH 6.5.

Excipients: CLNE is composed of DOTAP chloride, Squalene, Sorbitan Monostearate, Polysorbate

Physical Appearance description:

- It is an off-white to translucent homogenous liquid after reconstitution with WFI.

Description: GEMCOVAC-OM is a prophylactic vaccine for COVID-19. The size of the mRNA is 11701 bases. GEMCOVAC-OM is a novel mRNA-based vaccine candidate, which encodes the S-protein of the BA.1 Omicron variant of the SARS-CoV-2 virus as "antigen" and complexed with CLNE as a delivery system.

Pharmacotherapeutic group: medical immunobiological vaccine.

Comparator I:

Name: GEMCOVAC-19 [mRNA Vaccine for Injection (COVID-19)]

Dosage form: Lyophilized powder to be reconstituted with water for injection (WFI) to be given as intra-muscular injection

Composition per dose (0.5 mL):

Active substance: GEMCOVAC-19 formulation consists of an *in vitro* transcribed mRNA encoding for the S-protein and CLNE in a buffer containing 10% sucrose in 10 mM sodium citrate, pH 6.5.

Excipients: CLNE is composed of DOTAP chloride, Squalene, Sorbitan Monostearate, Polysorbate

Physical Appearance description:

It is an off-white to pale translucent homogenous liquid after reconstitution with WFI.

Description: GEMCOVAC-19 is a prophylactic vaccine for COVID-19. The size of the mRNA is 11709 bases. GEMCOVAC-19 is a novel mRNA-based vaccine candidate, which encodes the S-protein of the SARS-CoV-2 virus as "antigen" and complexed with CLNE as a delivery system.

Pharmacotherapeutic group: medical immunobiological vaccine.

Comparator II:

Brand Name: COVISHIELD™ (manufactured by Serum Institute of India Pvt Ltd)

Dose: 0.5 mL on Day 1

Composition: It contains ChAdOx1 nCoV- 19 Corona Virus Vaccine (Recombinant) 5×10^{10} viral particles (vp)

Recombinant, replication-deficient chimpanzee adenovirus vector encoding the SARS-CoV-2 Spike (S) glycoprotein. Produced in genetically modified human embryonic kidney (HEK) 293 cells.

Form:

Solution for injection

The solution is colourless to slightly brown, clear to slightly opaque and particle free with a pH of 6.6.

Pharmacological properties

The vaccine induces the formation of humoral and cellular immunity to the coronavirus infection induced by the SARS-CoV-2 virus.

Indications and usage:

Active immunization to develop immunological protection for prevention of severe acute respiratory syndrome coronavirus 2 (SARS-CoV-2) infection in adults ≥18 years.

1.3 Preclinical Data

Immunogenicity Studies:

The immunogenicity of GEMCOVAC-OM was assessed in mice and guinea pigs. GEMCOVAC-OM was injected into 10 C57BL/6 mice intramuscularly at 4 µg on Day 1 and Day 29. Blood was drawn at baseline, Day 28 and Day 43. There was an increase in the anti-spike IgG antibodies at Day 14, Day 28 and Day 43. Neutralizing antibodies assessed by cPASS and PRNT assay also showed an increase at Day 28 and Day 43 compared to the baseline.

Similarly, the immunogenicity of GEMCOVAC-OM was assessed in guinea pigs. GEMCOVAC-OM was administered intra-muscularly (2 and 5 µg) and intra-dermally (1, 2 and 5 µg) into 6 guinea pigs each at Day 1 and Day 29. Blood was drawn at baseline, Day 14, Day 28, Day 43 and Day 56. Vaccine administered by intradermal and intramuscular route induced immunogenic response in Guinea pigs at day 14, day 28, day 43 and day 56. At day 14, intradermal administration of 1µg, 2µg and 5µg dose generated higher immunogenic response than intramuscular 2µg and 5µg dose. At day 28 and day 43, immune response generated by 1µg intradermal dose was comparable to intramuscular 2µg and 5µg dose. Additionally, immunogenicity generated by intradermal administration of 2µg and 5µg dose was equivalent to intramuscular administration of 2µg and 5 µg at day 43. Intradermal administration of GEMCOVAC-OM shows better immune response at day 14 (after prime dose) and show equivalent immune response after boost, day 43. Therefore, intradermal route of administration can be used for generating IgG titers comparable to intramuscular route of administration. We observed a significantly elevated omicron-spike-specific B cell population as well as IFNγ expressing T cells in the vaccinated guinea pig lymph nodes.

Safety Study:

A GLP-compliant skin irritation study was conducted in new Zealand white rabbits. GEMCOVAC-OM was injected intradermally in a single dose at two sites, while the adjuvant control item was similarly injected at one site. No deaths or clinical signs of systemic toxicity were observed in treated rabbits during the period of this study. Body weights of treated rabbits were not affected during the study period. No gross pathological changes were observed during necropsy in tissues / organs of any of the rabbits in this study, when sacrificed on day-8. Observations of the skin revealed that intradermal injections to rabbits using the PharmaJet Tropis® device resulted in a reversible and a very slight, barely perceptible redness (grade 1) at the injection sites, not amounting to any significant irritation. Moreover, this minimal skin reaction, was comparable between the test vaccine and the adjuvant and hence was attributed not to the ‘antigenic’ components of the test vaccine, but to the ingredients of the adjuvant. It was

found to be reversible in nature. Microscopic examination of all sites of intradermal injections revealed an inflammation in dermis that was minimal in severity, multifocal in spread, and characterized by infiltration of inflammatory cells, predominantly comprising of macrophages, and less of neutrophils. These alterations were of reversible nature and were identified as the desired pharmacological effects of the ingredients of the adjuvant, and non-adverse in nature. GEMCOVAC-OM was found to be well tolerated at the intradermal injection sites in rabbit skin.

1.4 Clinical Data till Date

No clinical data is available for GEMCOVAC-OM. The proposed trial is the Phase II/III clinical study.

The GEMCOVAC-OM is based on the mRNA platform used for the development of GEMCOVAC-19 which has received Emergency Use Authorization for use as a prophylactic vaccine in COVID-19

<u>Summary of Adverse Events reported with GEMCOVAC-19</u>

The Phase I study conducted with GEMCOVAC-19 showed no dose limiting adverse events. No SAEs or death occurred with 5, 10 or 25 µg dose. Majority of the local and systemic solicited events were of Grade 1-2 intensity which resolved with symptomatic treatment. All doses were safe and well tolerated by the participants. Based on the results, the 10 µg dose was chosen.

The Phase II/III study is ongoing which will compare the safety and immunogenicity of GEMCOVAC-19 to COVISHIELD™. In the Phase II, it has been observed that the local and systemic solicited adverse events were a little higher in the participants receiving GEMCOVAC-19 compared to COVISHIELD™. However, this difference was limited to Grade 1 and 2 severities. There was no difference in the Grade ≥ 3 solicited adverse events. There were also no differences in the unsolicited adverse events in the two groups. There were no related serious adverse events in participants receiving GEMCOVAC-19.

In Phase III, there was no significant difference in the solicited and unsolicited adverse events in those receiveing GEMCOVAC-19 and COVISHIELD™.

1.4.1 Study Design Justification

<u>Rationale for booster dose</u>

After getting vaccinated against COVID-19, protection against the virus may be reduced due to decrease in neutralizing antibody levels over time. Moreover, the new variants such as Omicron have been shown to evade antibody recognition resulting in reduced efficacy of the vaccine. Emergence of highly transmissible variants of SARS-CoV-2 like omicron has led to considerations for booster doses to enhance immunity and provide sustained protection from COVID-19.[23] Emerging evidence shows that among healthcare and other frontline workers, vaccine effectiveness against COVID-19 decreases rapidly due to the combination of waning immunity and the greater exposure to virus including variants.

A clinical trial involving booster shot of BNT162b2 (Pfizer's mRNA-based vaccine) showed an increase in the immune response in trial participants, who had completed primary vaccination 6 months before.[23]

The officials involved in public health from USA have taken the decision to encourage coronavirus booster shots, after reviewing data showing that vaccine-produced immunity to milder infection decreases over time.[24] Recent findings from Israel and Qatar[25] reported an increasing proportion of breakthrough cases among the earliest vaccinated individuals. The transmissible delta variant and the observed waning protection against symptomatic infection with time since vaccination justify the need for a booster dose in healthy adult subjects as a booster dose can dramatically increase the amount of circulating antibodies.[26]

US- FDA has already authorized Omicron-specific booster doses of Pfizer and Moderna vaccines to certain high-risk adults, 6 months after the primary vaccination. US FDA has also authorized use of a single booster dose of the Janssen COVID-19 Vaccine that may be administered at least 2 months after completion of the single-dose primary regimen to individuals 18 years of age and older. US-FDA has also allowed "mix and match" vaccines i.e., getting a booster shot of a different vaccine than the one individuals received as their primary vaccines. [24]

In light of the above data and recommendations for variant specific booster doses by different WHO and regulatory authorities, a prospective, multi-centre, open-labelled, randomized, phase II study seamlessly followed by a Phase III study to evaluate the safety, tolerability and immunogenicity of GEMCOVAC-OM as a booster dose is proposed to be conducted in subjects 18 years of age and older.

Rationale of comparator

In the Phase II study, the safety and immunogenicity of GEMCOVAC-OM is being compared to the prototype vaccine GEMCOVAC-19. GEMCOVAC-19 has received emergency use authorization and safety data has already been generated using this vaccine. GEMCOVAC-OM has been designed using the sequence of the Omicron variant of the SARS-COV-2.

In Phase III study, COVISHIELD™ will be used as a comparator. COVISHIELD™ has been approved as a precautionary third dose in India in participants who have received COVISHIELD™ as the primary vaccine. A study has been published that has shown that a third dose of COVISHIELD™ increases the neutralization against all variants of SARS-CoV-2 including Omicron.[27] This makes COVISHIELD™ an ideal comparator.

1.4.2 Risk-Benefit Ratio

Potential benefits

Participants in this study will have already received a 2 dose primary regimen of COVISHIELD™ or COVAXIN™. Administration of the third dose 'boost' of COVID-19 vaccine in this study may be administered earlier than it would be through routine immunisation which is of potential benefit. It is hoped that the information gained from this study will contribute to the development of a safe, effective and versatile vaccine programme against COVID-19.

Potential risks

Associated with phlebotomy

Localised bruising and discomfort can occur at the site of venepuncture. Infrequently fainting may occur. These will not be documented as AEs if they occur. Approximately 65 mL of blood will be drawn from subjects receiving booster dose during the period of 6 months from Visit 1 (blood volumes may vary slightly for participants at different investigator sites due to use of different volume vacutainers, following local SOPs). This should not compromise these otherwise healthy volunteers, as these volumes are within the limits of 470 mL every 3 – 4 months for blood donations to the National Blood Transfusion Service. Participants will be asked to refrain from blood donation for the duration of their involvement in the trial.

Allergic reactions

Allergic reactions from mild to severe may occur in response to any constituent of a medicinal product's preparation. Anaphylaxis is known to occur in approximately 2.5 to 4.7 per million recipients of mRNA COVID-19 vaccines[28], and more generally in around 1 in 1,000,000 doses of all vaccines, but can occur in response to any vaccine or medication[29].

Behaviour change

Participants might feel they can modify their COVID-19 risk behaviours on the assumption that they are protected once vaccinated. Participants will be extensively counselled that they should continue to follow all up to date government advice in relation to COVID-19 precautions during the trial.

Specific risks from vaccines

The most common AEs with the vaccine includes chills, fatigue, headache, joint pain, malaise, and muscle ache, and were mostly seen in the 48 hours post vaccination.

Reactogenicity

Preliminary data from the "Comparing COVID-19 vaccine schedule combinations" study (COMCOV) has indicated that there may be increased reactogenicity at the second dose of vaccine from heterologous 2 dose primary regimens than from homologous.[30] It is possible that participants might experience more reactogenicity if their booster vaccine is different to the vaccine used in their 2 dose primary regimen.

2 STUDY OBJECTIVES AND ENDPOINTS

Phase II

**Study Objectives**	**Study End-points**
**Primary Objective**	**Primary End-points**
1. To assess the safety of adult subjects who received GEMCOVAC-OM as a booster dose till Day 180	• Occurrence and severity of local and systemic reactogenicity adverse events (AEs) for 7 days following vaccination<br>• Occurrence of unsolicited adverse events up to day 29 post vaccination<br>• Occurrence of related unsolicited adverse events throughout the duration of the study<br>• Occurrence of serious adverse events (SAEs): throughout the duration of the study
2. To evaluate the immunogenicity as detected by Immunoglobulin G- Enzyme-linked immunosorbent assay (IgG ELISA) against the SARS-CoV-2 Spike protein of GEMCOVAC-OM in comparison with GEMCOVAC-19 at Day 29	• Comparison of anti-Spike (omicron variant) IgG Antibodies (GMT) at Day 29 with GEMCOVAC-OM against GEMCOVAC-19
**Secondary Objective**	**Secondary End-points**
1. To evaluate the immunogenicity as detected by IgG ELISA against the SARS-CoV-2 Spike protein of GEMCOVAC-OM in comparison with GEMCOVAC-19 at Day 29	• Comparison of seroconversion rates as assessed by ≥ 2- fold rise in antibody titers at Day 29 from baseline
2. To evaluate the NAb against SARS-CoV-2 post vaccination with GEMCOVAC-OM in comparison with GEMCOVAC-19 at Day 29	• Comparison of neutralizing antibodies against SARS-CoV-2 using a surrogate virus assay (cPASS™ neutralization antibody kit) at Day 29
3. To evaluate cellular immune response from GEMCOVAC-OM in comparison with GEMCOVAC-19 at Day 29	• Cell mediated immunity assessment by cytokine expression from stimulated PBMCs at Day 29 (20% of participants)

Study Objectives	Study End-points
**Exploratory Objective**	**Exploratory End- points**
1. To evaluate the immunogenicity detected by IgG ELISA against the SARS-CoV-2 Spike protein of GEMCOVAC-OM at Day 90	• GMT measured by IgG-ELISA against SARS-CoV-2 Spike protein (omicron variant) post booster administration at Day 90
2. To evaluate the NAb against SARS-CoV-2 post vaccination with GEMCOVAC-OM at Day 90	• Neutralisation antibodies against SARS-CoV-2 using a surrogate virus assay (cPASS™ neutralization antibody kit) post booster administration at Day 90
3. To evaluate the cellular immune response from GEMCOVAC-OM at Day 90	• Assessment of cellular immune responses from stimulated PBMCs at Day 90 (20% of participants)
4. To assess symptomatic COVID-19 events till end of the study	• Symptomatic laboratory confirmed COVID-19 cases throughout the duration of the study

Phase III

Study Objectives	Study End-points
**Primary Objective**	**Primary End-points**
1. To evaluate the neutralizing antibody (NAb) titers against SARS-CoV-2 post vaccination with GEMCOVAC-OM in comparison with COVISHIELD™ at Day 29	• Comparison of neutralizing antibody titers against SARS-CoV-2 (omicron variant) using plaque reduction neutralization test (PRNT) assay with COVISHIELD™ at Day 29 by non-inferiority<br>• Comparison of seroconversion rates as assessed by ≥ 2- fold rise in neutralizing antibodies against SARS-CoV-2 (omicron variant) using PRNT at Day 29 between GEMCOVAC-OM and COVISHIELD™ by non-inferiority
**Secondary Objective**	**Secondary End-points**
1. To assess the safety of adult subjects who received GEMCOVAC-OM as booster dose till Day 180	• Occurrence and severity of local and systemic reactogenicity adverse events (AEs) for 7 days following vaccination<br>• Occurrence of unsolicited adverse events up to day 29 post vaccination<br>• Occurrence of related unsolicited adverse events

Study Objectives	Study End-points
	throughout the duration of the study<br>• Occurrence of serious adverse events (SAEs): throughout the duration of the study
2. To evaluate the immunogenicity as detected by IgG ELISA against the SARS-CoV-2 Spike protein with GEMCOVAC-OM in comparison with COVISHIELD™ at Day 29	• Comparion of anti-Spike (omicron variant) IgG antibodies (GMT) between GEMCOVAC-OM and COVISHIELD™ at Day 29<br>• Comparison of seroconversion rates as assessed by ≥ 2- fold rise in antibody titers at Day 29 between GEMCOVAC-OM and COVISHIELD™ using test of non-inferiority
3. To evaluate the NAb against SARS-CoV-2 post vaccination with GEMCOVAC-OM in comparison with COVISHIELD™ at Day 29	• Comparison of neutralizing antibodies against SARS-CoV-2 using a surrogate virus assay (cPASS™) at Day 29
4. To evaluate the cellular immune response from GEMCOVAC-OM in comparison with COVISHIELD™ at Day 29	• Cell mediated immunity assessment by cytokine expression from stimulated PBMCs at Day 29 (25% of participants)
**Exploratory Objective**	**Exploratory End-points**
1. To evaluate the immunogenicity detected by IgG ELISA against the SARS-CoV-2 Spike protein of GEMCOVAC-OM at Day 90	• GMT measured by IgG-ELISA against SARS-CoV-2 Spike (omicron variant) protein post booster administration at Day 90
2. To evaluate the NAb against SARS-CoV-2 post vaccination with GEMCOVAC-OM at Day 90	• Neutralisation antibodies against SARS-CoV-2 using a surrogate virus assay (cPASS™ neutralization antibody kit) post booster administration at Day 90<br>• GMT of SARS-CoV-2 specific serum neutralizing antibody levels using live virus ($PRNT_{50}$) assay, at Day 90
3. To evaluate the cellular immune response GEMCOVAC-OM at Day 90	• Assessment of cellular immune responses from stimulated PBMCs at Day 90 (25% of participants)
4. To evaluate COVID-19 infections	• Symptomatic Laboratory confirmed COVID-19 cases throughout the duration of the study

3 STUDY DESIGN

3.1 General Design

This is a Phase II seamlessly followed by Phase III study to evaluate the immunogenicity and safety of a booster dose of the Omicron-specific mRNA vaccine – GEMCOVAC-OM. The study participants will be adult subjects who are fully vaccinated against COVID-19 with either COVAXIN™ or COVISHIELD™ and received last dose of primary vaccination at least 4 months prior to screening. This booster dose of GEMCOVAC-OM will be based on the sequence of Omicron variant BA.1 of SARS-CoV-2. The study will be conducted in two parts: Phase II part will be conducted in 140 subjects while Phase III part of the study will enrol 3140 subjects.

Phase II

Approximately 140 subjects will be randomized, and the enrolment will be competitive. Hence, the subjects will be randomized in 1:1 ratio into two arms:

Arm I: 70 Subjects who have received either COVAXIN™ or COVISHIELD™ as primary vaccination (both doses) will receive a booster dose of GEMCOVAC-OM (intra-dermal).

Arm II: 70 Subjects who have received either COVAXIN™ or COVISHIELD™ as primary vaccination (both doses) will receive a booster dose of GEMCOVAC-19 (Intramuscular)

It will be ensured that at least 20% (n ~14) of the participants in each arm with receive COVAXIN™ and COVISHIELD™.

All the subjects randomized in Arm I will receive 1 dose of GEMCOVAC-OM and those who are randomized in Arm II will receive 1 dose of GEMCOVAC-19 (Intramuscular) on Day 1. GEMCOVAC-OM will be administered intradermally by PharmaJet Tropis Needle-Free Injector.

Phase III

Approximately 3140 subjects will be randomized, and the enrolment will be competitive. The subjects will be randomized into two arms:

Arm I: 3000 Subjects who have received either COVAXIN™ or COVISHIELD™ as primary vaccination (both doses) will receive a booster dose of GEMCOVAC-OM

Arm II: 140 Subjects who have received COVISHIELD™ as primary vaccination (both doses) will receive a booster dose of COVISHIELD™

All the subjects randomized in Arm I will receive 1 dose of GEMCOVAC-OM and subjects randomized in Arm II will receive 1 dose of COVISHIELD™.

The immunogenicity subset will include 280 subjects from Arm I and all the subjects from Arm II (n=140). The dose of GEMCOVAC-OM will be administered by PharmaJet Tropis Needle-Free Injector.

In the immunogenicity cohort of Arm I, it will be ensured that a minimum of 42 participants (15%) will have receieved COVAXIN™ and COVISHIELD™ as their primary vaccination.

In the safety cohort of Arm I, a minimum of 300 participants (10%) will have receieved COVAXIN™ and COVISHIELD™ as their primary vaccination.

Each subject must agree to participate in screening procedures by signing the most recent Ethics Committee approved Informed Consent Form (ICF) before any screening procedure is initiated. Each subject will be assigned a unique screening number on first cum first basis. Subjects satisfying the inclusion and none of the exclusion criteria will be randomized in the study.

Subjects who qualify screening assessments will be randomized in the study and will receive single dose of booster vaccine on Visit 1 (Day 1).

Subjects will be assessed for infection risk category at screening. Physical examination/ Assessment of the main vital signs will be performed at all onsite visits. /SAEs will be recorded throughout the study. Urine pregnancy test will be performed at screening for female of childbearing potential.

The subjects will have post vaccination telephonic follow-up on Day 7.

A thermometer will be provided to all the randomized subjects, and they will be instructed to record their daily body temperature, for a period of 1 week after booster dose administration/randomization in e-diary/paper diary, wherever usage of e-diary is not feasible.

Immunogenicity assessments will be performed in all the randomized subjects as per schedule of events.

All subjects receiving booster dose will participate in this clinical trial for approximately 6 months (Day 180+14 days) after the administration of the vaccine. The participants will have one screening/ vaccination visit and three on-site follow- up visits during the trial period.

The sites will collect the subject data using appropriate case report forms. Institutional Review Board/Institutional Ethics Committee approval will be taken prior to study initiation.

Study procedures will be as per assessment schedule.

The flowchart for the trial is presented in **Figure 1**

Figure 1 Flowchart of the study.

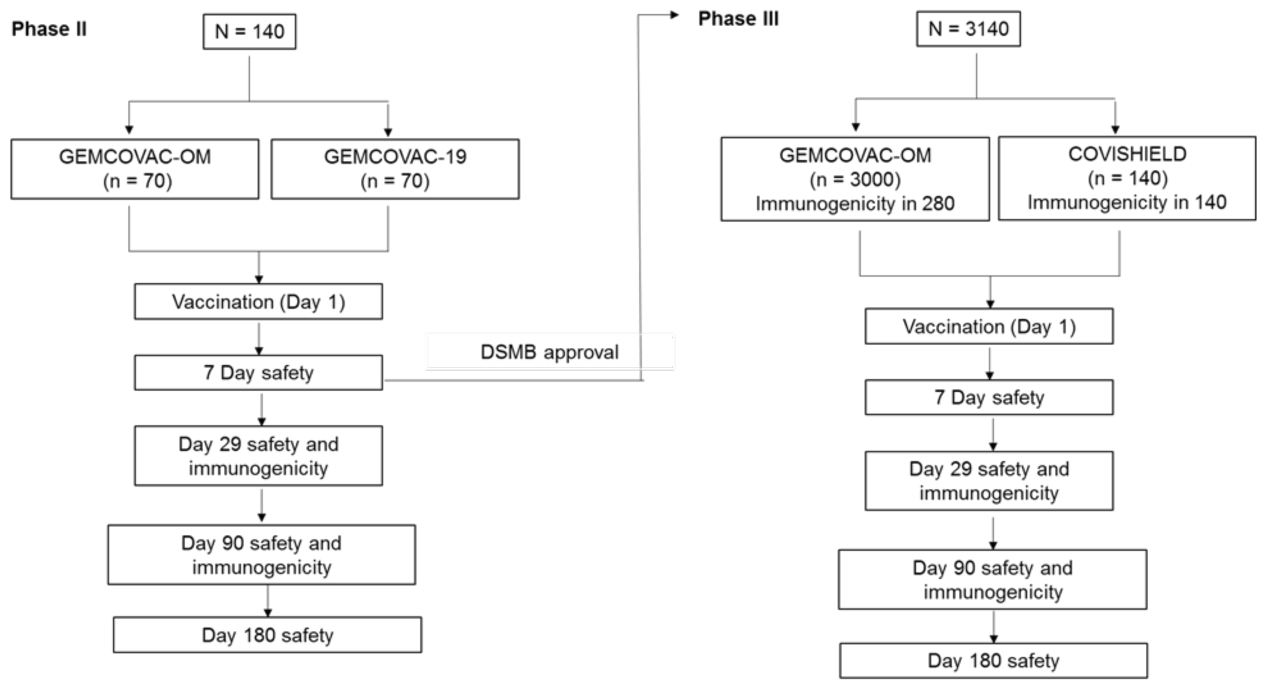

In Phase II, participants who have received 2 doses of either COVAXIN™ or COVISHIELD™ will be enrolled in both the arms. It will be ensured that at least 20% (n ~14) of the participants in each arm with receive COVAXIN™ and COVISHIELD™.

In Phase III, participants who received 2 doses of either COVAXIN™ or COVISHIELD™ will be enrolled in the GEMCOVAC-OM booster arm. Whereas, in the COVISHIELD™ booster arm, participants who have received 2 doses of COVISHIELD™ only will be enrolled. In the immunogenicity cohort of Arm I, a minimum of 42 participants (15%) will have receieved COVAXIN™ and COVISHIELD™ as their primary vaccination. In the safety cohort of Arm I, a minimum of 300 participants (10%) will have receieved COVAXIN™ and COVISHIELD™ as their primary vaccination.

3.1.1 Interim Analysis

An interim analysis for Phase II, and Phase III of the study will be performed when all subjects will complete Visit 2 (Day 29) assessment of immunogenicity. As the primary endpoint assessment is covered in the interim analysis, no adjustment for level of significance has been done in the sample size justification for interim analysis.
The Phase III interim immunogenicity and safety data will be submitted to the office of DCGI for Emergency Use Authorization.

3.1.2 Duration of Study

The duration of study will be approximately 6 months after randomization for each subject.

3.2 Early Termination of the Study

This study may be terminated early at the discretion of the study organizer and/or Regulatory Authority.

Schedule of Visits and Study Procedures

Table 1 Schedule of Visits (Phase II & Phase III)

Visit schedule	V1 (Screening Visit/ Baseline/Randomization Visit & Booster dose administration)	TC (Safety)	V2 (Safety & Immunogenicity)	V3 (Safety & Immunogenicity)	V4 (Safety)
	Day 1	Day 7 + 3	Day 29+7 – in-person visit	Day 90+14– in-person visit	Day 180+14 – in-person visit (EOS)
Informed consent	X				
Evaluation of inclusion/ exclusion criteria, taking the history, demographic[1] and anthropometric data	X				
Assessment of infection risk category[$]	X				
RT-PCR testing for COVID-19[@]	X				
Randomization	X				
Physical examination[2]/ Assessment of the main vital signs[3]	X		X	X	X
Urine pregnancy test[4]	X				
Study Product administration[5]	X				
Subjects filling out diaries[6]	X	X			
Concomitant Medications	X	X	X	X	X

Visit schedule	V1<br>(Screening Visit/ Baseline/Randomization Visit & Booster dose administration)	TC<br>(Safety)	V2<br>(Safety & Immunogenicity)	V3<br>(Safety & Immunogenicity)	V4<br>(Safety)
	Day 1	Day 7 + 3	Day 29+7 – in-person visit	Day 90+14– in-person visit	Day 180+14 – in-person visit (EOS)
AE/SAE's reporting	X	X	X	X	X
Post booster dose administration/randomization telephonic follow-up		X			
Assesment of Neutralizing Antibodies	X[#]		X	X	
Evaluation of Anti-S IgG Antibodies	X[#]		X	X	
Cellular Immunity	X[#]		X	X	

AEs=adverse events; COVID-19=Coronavirus 2019; EOS=End of Study; IC=informed consent; RT-PCR=Reverse transcriptase Polymerase Chain Reaction; SAEs=serious adverse events; V=Visit;.TC= Telephonic Consultation

[1]Demographics include Date of Birth or age, height, weight, and Sex.

[2]Physical examination includes assessment of the following body parameters: condition of skin, locomotive system, gastrointestinal tract, respiratory organs, cardiovascular system, urogenital system. Examination of lymph nodes (submandibular, cervical, cubital, inguinal lymph nodes) by touch should include assessment of their size, consistency, tenderness, mobility, matting with each other and with surrounding tissues and skin as per the investigator's discretion.

[3]Vital signs include Body temperature, Heart rate, Respiratory rate, systolic and diastolic blood pressure.

[4]Urine pregnancy test will be performed and if positive then Serum pregnancy test will be done for confirmation.

Blood sampling will be performed on day 1 after the subject is randomized to either 'booster arm' or 'comparator arm'. The sampling is to be performed immediately prior to the Study Product administration

[5] a)For subjects receiving the booster dose or comparator vaccine, If the body temperature is higher than 37.0°C on the day of administration of booster dose, the randomization visit can be postponed to the following day. Postponing the administration of booster dose for medical reasons (e.g., due to fever, common cold, diarrheal disease) will not be considered a deviation from the trial protocol.

b) GEMCOVAC-OM will be administered by PharmaJet Tropis Needle-Free Injector.

[6]All subjects must complete diary till Day 7 post booster dose administration, as well as when they feel unwell or when their condition deteriorates

[@]Unscheduled visits for subject with suspected COVID-19 (illness visits) should be done with PCR testing. Subjects found RT-PCR positive at screening, will not be included for analysis.

Note: As per study physician's discretion, the subjects may be asked for an extra telephonic follow-up and unscheduled onsite visit at any time during the clinical study for safety reasons to repeat study assessments and procedures. The subjects during the study period can visit the study site over and above the scheduled visits stipulated in the protocol for any safety concerns including COVID-19 infection, influenza, and/or ARVI (Acute Respiratory Viral Infection) clinical signs, who may be subjected to laboratory test

[$] a) A high risk means that the job involves interactions with the patients having the confirmed COVID-19 diagnosis

b) A medium risk means a professional contact with many people (general medical officers, social services employees, shop assistants, etc.)

c) A general risk means that there are no additional risks related to professional activities

3.3 Study Procedures

3.3.1 Visit 1 Screening/ Baseline and Randomization Visit (Day 1)

A screening period in the study begins from signing of the informed consent. No procedures related to the study are allowed with regard to the subject until the informed consent form is signed.

During Visit 1, the subjects will be assessed for inclusion and exclusion criteria. The potentially eligible subjects will be offered to participate in the clinical study.

During the screening visit, the following procedures will be carried out:

- Signing of the informed consent form by the study subject to participate in the study
- Collection of a history, demographic and anthropometric data: collection of data on concomitant and previous diseases, concomitant therapy, collection of information on allergies, study subject's contacts with patients with COVID-19, previous vaccinations/immunizations, blood donation
- Collection of demographic data: age, height, weight, and Sex. Body mass index (BMI) is measured according to the following formula: BMI = weight/height^2 (kg/m^2), where the weight is in kilograms and the height is in centimetres
- Medical/Surgical and medication history: Concomitant diseases include problems found during physical or instrumental examination, which will be described in the study subject's source documents and in an electronic case report form, including information on medical history data of the subjects included in the study. When describing a concomitant disease or medical condition, the date when such condition started, and the therapy (if any) conducted for this reason should be indicated. Medical/Surgical History and Concomitant Medications will be recorded at screening
- Physical examination includes assessment of the following body parameters: condition of skin, locomotive system, gastrointestinal tract, respiratory organs, cardiovascular system, urogenital system. Examination of lymph nodes (submandibular, cervical, cubital, inguinal lymph nodes) by touch should include assessment of their size, consistency, tenderness, mobility, matting with each other and with surrounding tissues and skin as per the investigator's discretion. Auscultation data should include description of breathing pattern (vesicular breathing, rattling)
- Assessment of vitals: heart rate, respiratory rate, systolic and diastolic blood pressure, body temperature. Heart rate and blood pressure should be measured after a subject has 5 minutes of rest sitting
- A swab from nose and/or throat will be collected at Screening for testing of SARS-CoV-2 by RT-PCR
- Urine Pregnancy test will be performed in females of childbearing potential

- Blood sampling
- Study Vaccine /Comparator Vaccine administration [at Day 1]: After the Study Vaccine / Comparator Vaccine administration during randomization visit, drinking as well as motion activity (except for heavy physical exertion) are unrestricted. The study subject must abstain from intensive physical exercises and combat sports for 24 hours prior to the beginning of and throughout the study. The study subject should not take any drugs/medications without agreement by the study physician (including botanical medicines and nutritional supplements). Physical examination will be performed 30 minutes post booster dose

  Note: For subjects receiving the booster dose or comparator vaccine, If the body temperature is higher than 37.0° C on the day of administration of booster dose, the randomization visit can be postponed to the following day. Postponing the administration of booster dose for medical reasons (e.g. due to fever, common cold, diarrheal disease) will not be considered a deviation from the trial protocol.

- Subject diary (E-diary/paper diary, if e diary usage is not feasible) will be issued and training will be given by study Investigator to the subject
- Concomitant medications will be recorded
- Any immediate AEs/SAEs post vaccination will be recorded

3.3.2 Telephonic Visit (Safety) ([Day 7+3]

- AEs/SAEs will be recorded.
- Concomitant Medications will be recorded
- Subjects will fill out their diaries till day 7

Note: Post booster dose administration / randomization telephonic follow-up will be conducted. Subject will be enquired for any complaints

3.3.3 In-person visit ([Safety & Immunogenicity] [Visit 2-Day 29+7]/ [Visit 3-Day 90+14]/ [End of Study (Visit 4-Day 180+14)]

- Physical examination includes assessment of the following body parameters: condition of skin, locomotive system, gastrointestinal tract, respiratory organs, cardiovascular system, urogenital system. Examination of lymph nodes (submandibular, cervical, cubital, inguinal lymph nodes) by touch should include assessment of their size, consistency, tenderness, mobility, matting with each other and with surrounding tissues and skin as per the investigator’s discretion. Auscultation data should include description of breathing pattern (vesicular breathing, rattling).
- Assessment of vitals: heart rate, respiratory rate, systolic and diastolic blood pressure, body temperature. Any changes in the vitals as compared to the source parameters, which are regarded as adverse events, should be duly recorded in the source documents and in the electronic case report form

- Blood sampling*
- Concomitant medications will be recorded
- AEs/SAEs will be recorded

*Blood sampling will not be performed at EOS (Day 180+14)

3.3.4 Telephonic Follow-up/ Consultation

The subjects will also have remote telephonic consultations with the study physician on Day 7 after booster dose administration. Subjects will be interviewed for any new complaints, and/or previous disease, concomitant therapy use, any specific symptoms related to COVID-19 or contact history of SARS-CoV-2 positive patients. In case, subjects have symptoms related to COVID-19 such as fever, chills, rhinorrhea, sore throat, cough, expectoration, fatigue, weakness, malaise, headache, muscle pain, gastrointestinal symptoms-specifically diarrhoea and vomiting, shortness of breath or dyspnoea, loss of smell (anosmia), loss of taste [ageusia]), subjects will be advised to visit the study site for RT-PCR testing.

3.3.5 Unscheduled Visits

At the study team's discretion, the subjects may be invited to a tele-consultation and an in-person unscheduled visit at any time during the clinical study for safety reasons or if the subject has complaints indicative of potential COVID-19, influenza, and/or a common cold.

During unscheduled visits, the study physician may carry out any required assessments, including ordering necessary laboratory and instrumental examinations, not stipulated by this study protocol. If the subject has symptoms indicative of COVID-19 disease, a RT-PCR test will be performed for further confirmation of the disease. Investigators should follow the algorithm for COVID-19 case confirmation as per the guidelines issued by the Ministry of Health and Family Welfare (MoHFW), Govt. of India.

COVID-19 confirmation visit can be performed at subject's home.

Any unscheduled visits must be recorded in the source documents and the electronic case report form. Such unscheduled visits should not affect the schedule of regular visits stipulated by the study protocol.

3.4 Study Assessments

3.4.1 Demographics

Collection of a history, demographic and anthropometric data: collection of data on concomitant and previous diseases, concomitant therapy, collection of information on allergies, study subject's contacts with patients with COVID-19, previous vaccinations/immunizations, blood donation. Collection of demographic data: sex, race, age. Subject's height and weight are measured. The BMI is measured according to the following formula: BMI = weight/height^2 (kg/m^2), where the weight is in kilograms and the height is in centimetres. The demographics will be recorded at screening.

3.4.2 Medical/Surgical History and Concomitant Medications

Concomitant diseases include problems found during physical or instrumental examination, which will be described in the study subject's source documents and in an electronic case report form, including information on medical history data of the subjects included in the study. When describing a concomitant disease or medical condition, the date when such condition started and the therapy (if any) conducted for this reason should be indicated. Medical History and Concomitant Medications will be recorded at screening.

3.4.3 Vital Signs

Assessment of vitals: heart rate, respiratory rate, systolic and diastolic blood pressure, body temperature. Heart rate and blood pressure should be measured after a subject has 5 minutes of rest sitting. Any changes in the vitals as compared to the source parameters, which are regarded as adverse events, should be duly recorded in the source documents and in the electronic case report form. The assessment of vital signs will be performed as specified in the Schedule of Events **Table 1.**

3.4.4 Physical Examination

Physical examination includes assessment of the following body parameters: condition of skin, locomotive system, gastrointestinal tract, respiratory organs, cardiovascular system, urogenital system. Examination of lymph nodes (submandibular, cervical, cubital, inguinal lymph nodes) by touch should include assessment of their size, consistency, tenderness, mobility, matting with each other and with surrounding tissues and skin as per the investigator's discretion. Auscultation data should include description of breathing pattern (vesicular breathing, rattling). The assessment of Physical examination will be performed as specified in the Schedule of Events **Table 1.**

3.4.5 Pregnancy Test

For women of child bearing potential, a urine pregnancy test will be performed at screening. Urine pregnancy test will be performed and if positive then Serum pregnancy test will be done for confirmation.

3.4.6 Immunogenicity Assessment

Immunogenicity after the Study Vaccine/ Comparator Vaccine administration will be assessed by determining the humoral mediated immune responses (specific antibody titers and virus-neutralizing antibody) and evaluating the antigen-specific cell-mediated immune responses.

<u>Blood Sampling</u>

Blood sampling for immunogenicity assessment will be carried out in all the subjects randomized in Phase II part of the study. In Phase III study, blood sampling will be done in 280 subjects included in Arm I and all the subjects included in Arm II (n=140).

Assessment of immunogenicity will be done by detection of IgG antibodies to SARS-CoV-2-S antigen (omicron variant) by ELISA (Enzyme-Linked Immunosorbent Assay); and neutralizing

antibody assay using a surrogate assay (cPASS™ neutralization antibody kit) and plaque reduction neutralization test ($PRNT_{50}$ against the BA.1 Omicron variant) assay.

Cell mediated immune responses will be assessed using the intracellular cytokine staining from stimulated PBMCs. The testing methods will be detailed in the Laboratory Manual.

Methods for Processing, Label and Storage of Blood Samples

Approximately 65 mL of blood will be drawn from subjects included in immunogenicity subset during the period of 6 months (study duration) from Visit 1 for immunogenicity assessment as shown in **Table 2.**

Table 2 Total Blood Sample for Assessment of Immunogenicity

Immunogenicity parameter	Blood Vol. (ml)	Blood Vol. (ml)	Blood Vol. (ml)	Total Vol. (ml)
Day	Day 1	Day 29+7	Day 90+14	
Humoral Immunity Response	5	5	5	15
Cellular Immune Response	16	16	16	48
Total Vol. (ml)	21	21	21	63

The blood will be processed and aliquoted according to the Laboratory Manual. All aliquots will be stored at a temperature of 2-8 °C or below labeled and shipped to the Immunogenicity laboratory for long-term storage (- 20 °C and below).

Complete instructions for labeling and storage of serum samples will be provided in the Laboratory Manual.

3.4.7 RT-PCR testing for SARS-CoV-2

A swab from nose and/or throat will be collected and send to local laboratory for testing of SARS-CoV-2 by RT-PCR at Screening.

*If the subject experiences any of the following symptoms (fever, chills, rhinorrhea, sore throat, cough, expectoration, fatigue, weakness, malaise, headache, muscle pain, gastrointestinal symptoms-specifically diarrhoea and vomiting, shortness of breath or dyspnoea, loss of smell [anosmia], loss of taste [ageusia]) then RT-PCR testing for SARS-CoV-2will be conducted at any time post booster dose administration.

In case subject had contact history of SARS-CoV-2 positive patients, subject will be advised to visit the study site for RT-PCR testing.

The enrollment of participants will not depend on the RT-PCR test. If the subject is positive, he will be a part of the study (safety cohort) but will be excluded from the immunogenicity analysis.

3.4.8 Adverse Events

Requirements for collecting, recording, and reporting of AEs are described in Section 9. Each subject will be evaluated from the day of signing of informed consent form till the End of Study.

4 SUBJECT SELECTION AND WITHDRAWAL

4.1 Subject Population

Subjects will be randomized in the study who meet all the inclusion criteria and none of the exclusion criteria

4.2 Inclusion Criteria

In order to be included in the trial, subjects must meet all the criteria listed below:

1. Male and female aged ≥ 18 years
2. Subject who had received primary vaccination (both doses completed) with either
    a. COVAXIN™ OR
    b. COVISHIELD™

    Wherein last dose of primary vaccination taken at least 4 months prior to the screening visit
3. Subject or their legally acceptable representative (LAR) should be capable and willing to give voluntary written informed consent prior to inclusion in the study
4. Inclusion of subjects based on clinical judgment by the investigator
5. Subjects who had COVID-19 infection after primary vaccination, should have been asymptomatic or RT-PCR negative for at least 3 months
6. Consent for using effective methods of contraception during the entire study period
7. No medical history of pronounced vaccine-induced reactions or complications after receiving immunobiological products
8. No acute infectious and/or respiratory diseases within 14 days prior to screening
9. Subjects able to comprehend and comply with study requirements and procedures and willing to complete subject diary

[1] With use of one of the following methods: abstinence, intrauterine device, oral or injectable contraceptive, subdermal contraceptive implant, double barrier method (for example, a condom with local action contraceptive lubricant). "Selected practice recommendations for contraceptive use, second edition, World Health Organization, 2005: ISBN 92:4 156284.6 (NLM classification: WP 630)

4.3 Exclusion Criteria

Subjects will be entered into the study only if they meet none of the following criteria.

1. Prior receipt of any COVID-19 vaccine in less than 4 months of duration
2. Pregnant or lactating mothers
3. Any significant illness or any other current or pre-existing health condition (e.g. any major pulmonary, cardiovascular, renal, neurological, metabolic, gastro-intestinal, hepato-biliary, haematological functional abnormality, mental or physical disability, blood

dyscrasia, major congenital defects, etc.) which in the opinion of the Investigator may affect the safety of the subject or the study endpoints

4. History of chronic infections in immunocompromised subjects
5. History of chronic immune disease, Splenectomy or systemic collagenosis
6. Subjects with oncological disease within 5 years prior to inclusion into the study
7. History of the human immunodeficiency virus, syphilis, hepatitis B, or C
8. Acute Kidney injury or dialysis, had transplant and on immunosuppressive therapy
9. Currently receiving or have received (in last 4 weeks) medication intended to prevent COVID-19 except for multi-vitamin supplements
10. Receipt of steroids and/or immunoglobulins or other blood products within 30 days prior to randomization
11. Tattoos or scars at the injection site, which in the medical opinion of the investigator does not allow assessing the local response to the study vaccine administration
12. Participation in other interventional clinical trial within the previous 90 days prior to randomization and over duration of the trial
13. Any other condition that the study physician considers as a barrier to the trial completion as per the protocol

4.4 Recommendations for Subjects

For the participation in the trial, each subject should adhere to the following recommendations:

1. Alcohol consumption should be avoided 2 days before and after the study product administration and refrain from alcohol abuse during the study duration
2. Refrain from any activities which require intense focus and fast psysical and motoric reactions within 48 hours after the administration of booster dose
3. Before trial participation, women (of child-bearing potential) must be informed about the importance of contraception during the trial and potential pregnancy risk factors, men must also follow recommendations on adequate contraception
4. Visiting the study centre on prescribed dates and time for tests as required
5. Having a telephone that allows the study physician to contact the subject during the study, access to telephone contact
6. Informing the study physician about your state of health and any change of personal condition
7. Filling out an electronic or paper diary regularly

4.5 Randomization

Subjects who meet the inclusion/exclusion criteria and have successfully completed all screening procedures will be randomized in the study by using interactive web response system (IWRS) method into two arms.

Subjects who have received COVAXIN™/COVISHIELD™ as primary vaccination (both doses) will receive either Study Vaccine/ Comparator Vaccine. However, in Phase III study, the Comparator Vaccine- COVISHIELD™ will be administered only to particpants who have received two doses of the COVISHIELD™ vaccine.

In Phase II, it will be ensured that at least 20% (n ~14) of the participants in each arm with receive COVAXIN™ and COVISHIELD™.

In the immunogenicity cohort of Arm I of Phase III, a minimum of 42 participants (15%) will have receieved COVAXIN™ and COVISHIELD™. In the safety cohort of Arm I of Phase III, a minimum of 300 participants (10%) will have receieved COVAXIN™ and COVISHIELD™.

Unique randomization codes will be assigned to the subjects and will remain unchanged until the completion of the trial. The randomization codes will be generated through Proc Plan using SAS® version 9.4 or higher (SAS Institute Inc, Cary, North Carolina) by an independent biostatistician.

A Randomization Strategy and IWRS implementation document will detail the elaborative randomization strategy for different subset population in the trial such as safety and immunogenicity indicators. Final randomization list will be filed securely by the independent biostatistician, and accessible to authorized persons only.

4.6 Early Withdrawal of Subjects from the Study

Subjects may voluntarily withdraw their consent to participate in this trial at any time for whatever reason. The consent is withdrawn only in case when the subject does not wish to participate in the study, refuses from any subsequent visits or assessments as well as from any subsequent study-related procedures.

The study organizer will continue to keep and use all data of the study subject and biological samples already collected.

In case of the consent withdrawal, the study physician should take reasonable measures to eliminate the main reason of such decision and document this information. In case where the subject is withdrawn within 30 days of the administration of Study Vaccine/ Comparator Vaccine, then Investigator should make all reasonable measures to follow-up the subjects for at least 30 days after the administration of Study Vaccine/ Comparator Vaccine.

For early withdrawal of a subject from the study, all safety assessments related to End of study visit will be performed. If the subject notifies of his/her intention to withdraw the consent, stops visiting the study physician or there will be no communication with him/her during the study, such subject may be deemed as left the study.

The study physician may discontinue the treatment of the subject in accordance with the study protocol, if, in the study physician's opinion, remaining of the subject in the study may negatively

affect the subject. Treatment under the study protocol should also be discontinued in any of the following situations:

- Development of a serious adverse event that, in the opinion of the study physician, prevents the subject from further participating in the study
- Pregnancy
- Protocol deviations having significant risk for the study subject's safety, including application of excluded medication
- Positive RT-PCR test of SARS-CoV-2 post administration of Study Vaccine / Comparator Vaccine: the subject will be followed-up till resolution.

Subjects discontinued the treatment under the study protocol should not be deemed as left the study. The study physician should take all possible measures to continue observation over the subject to assess the disease, his/her condition, and other parameters.

5 STUDY PRODUCT

5.1 Description

Investigational Product (IP)

Description: GEMCOVAC-OM a novel mRNA-based vaccine candidate, which encodes the BA.1 omicron variant of the S- protein of SARS-CoV-2 virus as "antigen" and complexed with 'Cationic Lipid Nanoemulsion (CLNE)' as a delivery system.

Name: GEMCOVAC-OM

Dose & Route: a 0.1 mL [10 µg Lyophilized powder to be reconstituted with water for injection (WFI)] to be given intradermally. The dose will be administered by PharmaJet Tropis Needle-Free Injector.

Composition per dose: The vaccine contains:

Active substance: GEMCOVAC-OM formulation consists of an *in-vitro* transcribed mRNA encoding for the BA.1 omicron variant of the S-protein and CLNE in a buffer containing 10% sucrose in 10 mM sodium citrate, pH 6.5

It is an off-white to pale translucent homogenous liquid after reconstitution with WFI.

Excipients:

CLNE is composed of DOTAP chloride, Squalene, Sorbitan Monostearate, Polysorbate 80 and Sodium Citrate Dehydrate.

Comparator I:

Name: mRNA vaccine – GEMCOVAC-19 (Intramuscular)

Form: Lyophilized powder for solution for injection to be given intra-muscularly.

Dose: 0.5 mL (10 µg) on Day 1.

Composition: GEMCOVAC-19 formulation consists of an in-vitro transcribed mRNA encoding for the S-protein and CLNE in a buffer containing 10% sucrose in 10 mM sodium citrate, pH 6.5.

Excipients: CLNE is composed of DOTAP chloride, Squalene, Sorbitan Monostearate, Polysorbate 80 and Sodium Citrate Dehydrate

Comparator II:

Brand Name: COVISHIELD™ (manufactured by Serum Institute of India Pvt Ltd)

Dose: 0.5 mL on Day 1

Composition: It contains ChAdOx1 nCoV- 19 Corona Virus Vaccine (Recombinant) 5×10^{10} viral particles (vp) *

Recombinant, replication-deficient chimpanzee adenovirus vector encoding the SARS-CoV-2 Spike (S) glycoprotein. Produced in genetically modified human embryonic kidney (HEK) 293 cells.

Form:

Solution for injection

The solution is colourless to slightly brown, clear to slightly opaque and particle free with a pH of 6.6.

5.2 Treatment Regimen

The Study Vaccine/ Comparator Vaccine will be administered to subjects during randomization visit [Day 1]:

5.3 Method for Assigning Subjects to Treatment Groups

All subjects who fulfil the inclusion/exclusion criteria and have successfully completed all screening procedures will be randomized to either receive GEMCOVAC-OM or Comparator Vaccine.

5.4 Preparation and Administration of Study Product

The Study Vaccine/Comparator Vaccine will be administered to the subjects via intra-dermal/intra-muscular injection during randomization visit. Intravenous injection of the Study Vaccine /Comparator Vaccine is not allowed.

Study vaccines should be prepared using aseptic technique.

At Visit 1 (Day 1) the subject will receive the Study Vaccine- GEMCOVAC-OM (Intradermal) packed in multidose vial.

Study Vaccine	Vaccine Administration

GEMCOVAC-OM	The lyophilized powder will be reconstituted in WFI (0.7 mL). Mix till the contents are thoroughly dissolved and no traces of undissolved powder are visible. 0.1 mL of the study vaccine containing 10 µg dose will be administered intradermally.<br>GEMCOVAC-OM will be administered intradermally by PharmaJet Tropis Needle-Free Injector.
GEMCOVAC-19	The lyophilized powder will be reconstituted in WFI (12 mL). Mix till the contents are thoroughly dissolved and no traces of undissolved powder are visible. 0.5 mL of the study vaccine containing 10 µg dose will be administered intramuscularly.
COVISHIELD™	0.5 mL of the vaccine will be administered intramuscularly.

5.5 Study Product Accountability

The study product required for this study will be provided by the Sponsor. Study Vaccine/Comparator Vaccine will be distributed through Seveillar Clinical Supplies Services Pvt Ltd. The study site (i.e., Investigator or other authorized personnel [e.g., Pharmacist]) is responsible for maintaining records of study product delivery to the site, study product inventory at the site, study product use by each subject, and return of unused study product, thus enabling reconciliation of all study product received, and for ensuring that subjects are provided with doses specified by the protocol.

The Investigator or designee will confirm that appropriate temperature conditions have been maintained during transit for the study products received at study site and that any discrepancies have been reported and resolved before use of the study products. All study products will be stored in a secure, environmentally controlled, and monitored (manual or automated) area in accordance with the labeled storage conditions, with access limited to the Investigator and authorized staff.

Separate Investigational product management manual will capture all the details related to order, supply, storage, dispensing, administration, and associated logs.

5.6 Subject Compliance Monitoring

The Study Vaccine /Comparator Vaccine will be administered during randomization visit [Day 1].

After the injection of the Study Vaccine /Comparator Vaccine, the trial subject must remain under medical supervision for 30 minutes.

For subjects receiving the Study Vaccine / Comparator Vaccine, if the body temperature of the subject is higher than 37.0°C on the day of administration of vaccine, the randomization visit can be postponed to the following day. Postponing the administration of booster dose for medical reasons (e.g., due to fever, common cold, diarrheal disease) will not be considered a deviation from the trial protocol.

5.7 Prohibited and Concomitant Therapy

5.7.1 Concomitant Medications

In this study, it is allowed to use drugs that are not listed in the Prohibited Therapy section, including nonsteroidal anti-inflammatory drugs (NSAIDs) and antihistamines to relieve undesirable effects in the subjects.

Drugs for adverse events treatment and emergency therapy (if necessary) shall be prescribed as per the recommendations for use and dosages of such drugs.

Each therapy shall be recorded in the eCRF and primary documentation of the study subject.

Administration of any concomitant drugs shall be recorded in the eCRF, which shall include the concomitant drug name and dosage, and reason for use of such drug.

The study centre shall have emergency medical facilities. In the event of emergency medical care, all measures will be taken, and the drugs used shall be recorded in the primary documentation and eCRF.

5.7.2 Prohibited Medications

- Subjects undergoing immunosuppressive therapy and immunocompromised subjects may not develop a sufficient immune response. Consequently, it is not recommended to use immunosuppressive drugs 30 days prior to and after the booster dose administration
- Prohibited therapy being a basis for excluding a subject from the study will also include administration of immunoglobulins or blood products throughout the study until the end of follow up
- No vaccination with any other vaccines, including in the course of other clinical studies, is permitted
- No transfusion of blood and its components, plasma exchange and donation are allowed throughout the study. No use of immunoglobulins, monoclonal antibodies, interferons, colony-stimulating, and growth factors is allowed. In case of a need for therapy applying the above drugs and procedures, the subject should be removed from the study

6 STATISTICAL PLAN

Prior to the analysis of the final study data, a detailed Statistical Analysis Plan (SAP) will be written describing all analyses that will be performed. The SAP will contain any modifications to the analysis plan described below.

6.1 Demographic and Baseline Characteristics

Demographic and baseline characteristics will be summarized descriptively for the safety set. The demographic variables include age, sex, weight, and height. Variables that are measured on a continuous scale, such as age of the subject at time of enrolment, number of non-missing observations (n), mean, median, SD, minimum, and maximum will be tabulated. Variables that are measured on a categorical scale will be summarized using frequencies and percentages.

6.1.1 Medical/ Surgical History, Concomitant Medication and Other Safety Evaluations

Medical history (recorded at the screening visit) will be coded using MedDRA version 23.0 or higher. The concomitant medications will be classified under ATC classification 2nd level therapeutic class and generic term using version 01 March 2021 or later version of WHO Drug Dictionary (WHODD). In addition, physical examination, vital signs, etc. will be summarized. The medical history, concomitant medications, and other safety evaluations will be tabulated by standard summary statistics based on the safety analysis set.

6.2 Analysis of Endpoints

In general, the variables will be summarized by using standard descriptive statistics. Continuous variables will be summarized with the number (n) of non-missing observations, mean, standard deviation, median, and minimum and maximum, unless otherwise specified. For categorical data, descriptive statistics will be presented with the number and percentage of subjects in the various categories of the endpoint. All the comparative analysis will be considered statistically significant at 5% level of significance unless stated otherwise. Statistical software SAS® version 9.4 or higher (SAS Institute Inc, Cary, North Carolina) will be used for the analyses.

6.2.1 Analysis of Primary Endpoints

The immunogenicity analysis will be performed on immunogenicity analysis set and safety analysis will be performed on safety analysis set.

In Phase II and Phase III, non inferiority margin will be considered as 0.67 as per WHO guidelines, i.e. non inferiority is demonstrated if the lower bound of 95% CI of GMT ratio (GEMCOVAC-OM/ COVISHIELD™) is > 0.67.

In Phase III, non-inferiority margin for seroconversion is defined as lower bound of 95% CI in seroresponse rate difference (Seroconversion Rate GEMCOVAC-OM - Seroconversion Rate COVISHIELD™) being > - 10%.

The interim Phase III immunogenicity and safety data at Day 29 will be analysed and submitted to the office of DCGI for Emergency Use Authorization.

Phase II

Immunogenicity Analysis

- Comparison of Anti-Spike (omicron variant) IgG Antibodies (GMT) at Day 29 with GEMCOVAC-OM against GEMCOVAC-19.

The GMTs of the Anti-Spike (omicron variant) IgG Antibodies (GMT) at baseline and Day 29 will be evaluated among subjects who receive booster dose. Summary statistics of GMTs will be calculated based on base 10 log-transformed titres. GMT ratio from pre-vaccination to post-vaccination in each arm will be compared between the subjects receiving GEMCOVAC-OM and those who received GEMCOVAC-19. The analysis will be performed by using analysis of covariance (ANCOVA). The GMT ratio will be the outcome variable, Study treatment will be main independent variable, baseline titres will be the covariate in the ANCOVA analysis.

- **Safety Analysis**
    - Occurrence and severity of local and systemic reactogenicity adverse events (AEs) for 7 days following vaccination.
    - Occurrence of unsolicited adverse events up to day 29 post vaccination
    - Occurrence of related unsolicited adverse events up to day 180 post vaccination
    - Occurrence of serious adverse events (SAEs): throughout the duration of the study
    - Occurrence of adverse events of special interest (AESI): throughout the duration of the study

Adverse events (AE) will be coded in accordance with the MedDRA version 23.0 or higher. Number of Solicited Aes, Unsolicited Aes, Serious Aes and Aes of special interest reported during the study will be presented with number and percentage of subjects who reported the corresponding events by vaccine and comparator arms. Also, 95% confidence interval (CI) for proportion of subjects will be calculated by using the exact binomial distribution from Clopper-Pearson's method.

Phase-III

- **Geometric Mean Titer Ratio:**
  To evaluate the neutralizing antibody (Nab) titers against SARS-CoV-2 post vaccination with GEMCOVAC-OM against COVISHIELD™ at Day 29.
  Geometric mean titres (GMT) of neutralizing antibody (Nab) titers against the BA.1 omicron specific SARS-CoV-2 will be calculated at baseline and at day 29 in both the treatment groups. The geometric mean titres (GMT) calculation will be performed by taking the anti-log of the mean of the log transformations using Analysis of Covariance (ANCOVA) in which the log transformed value of titres at Day 29 will be included as outcome variable, Treatment group as fixed effect and baseline log transformed titre as covariates. The GMT will be the anti-log value of least square mean obtained from the ANCOVA model and the GMTR will be the ratio of GMTs of the two groups.
  Based on the WHO guidelines, For NI if lower limit of two-sided 95% CI of GMT ratio > 0.67, then there is enough evidence to demonstrate non-inferiority of GEMCOVAC-OM to COVISHIELD™.
- **Seroconversion Rate**
  Seroconversion rate is defined as percentage of subjects who will show ≥ 2-fold rise in neutralizing antibody titers (PRNT assay using omicron variant) at Day 29 compared to baseline.

Number and percentage of subjects who achieve ≥2- fold rise will be presented for GEMCOVAC-OM and COVISHIELD™ group. 95% CI will be calculated for percentage by using Clopper-Pearson method. Difference between percentages and the 95% CI for the difference will also be calculated by using Meitinen-Nurminen method. Chi-square or Fisher's exact test will be used to compare the subjects who achieve ≥2 fold rise between GEMCOVAC-OM and COVISHIELD™ arms.

Non-inferiority margin for difference in seroconversion rates is considered as -10% (Seroconversion Rate $_{\text{GEMCOVAC-OM}}$ – Seroconversion Rate $_{\text{COVISHIELD™}}$), i.e. GEMCOVAC-OM vaccine will be considered as non-inferior to the COVISHIELD™ vaccine if the lower bound of 95% CI of the difference in Seroconversion Rates is > -10%.

6.2.2 Analysis of Secondary Endpoints

Phase-II

- Comparison of seroconversion assessed by ≥ 2 fold rise in antibody titers at Day 29 from baseline.
  Number and percentage of subjects who achieve ≥2 fold rise will be presented by GEMCOVAC-OM and GEMCOVAC-19 group. 95% CI will be calculated for percentage by using Clopper-Pearson method. Difference between percentages and the 95% CI for the difference will also be calculated by using Meitinen-Nurminen method. Chi-square or Fisher's exact test will be used to compare the subjects who achieve ≥2 fold rise between GEMCOVAC-OM and GEMCOVAC-19 arms.
- Comparison of neutralizing antibodies against SARS-CoV-2 (cPASS™) at Day 29.
  The median/ mean neutralization percentage at baseline and Days 29 will be evaluated among subjects who receive booster dose. Median/ mean of percentage from pre-vaccination to post-vaccination in each arm will be compared between the subjects receiving GEMCOVAC-OM and who received GEMCOVAC-19. The analysis will be performed by using analysis of covariance (ANCOVA). The difference in median/ mean percentage neutralization will be the outcome variable, Study treatment will be main independent variable and baseline neutralization percentage will be the covariate in the ANCOVA analysis.
- Cell mediated immunity assessment by cytokine expression from stimulated PBMCs at day 29 (20% of participants). Summary statistics of cell mediated immunity response will be provided by vaccine arms.

Phase-III

- Comparison of Anti-Spike (omicron variant) IgG Antibodies (GMT) at Day 29 with GEMCOVAC-OM against COVISHIELD™
  The GMTs of the Anti-Spike (omicron variant) IgG Antibodies (GMT) at baseline and Day 29 will be evaluated among subjects who receive booster dose. Summary statistics of GMTs will be calculated based on base 10 log-transformed titres. GMT ratio from pre-vaccination to post-vaccination in each arm will be compared between the subjects receiving GEMCOVAC-OM and those who received COVISHIELD™. The analysis will be performed by using analysis of covariance (ANCOVA). The GMT ratio will be the

outcome variable, Study treatment will be main independent variable, baseline titres will be the covariate in the ANCOVA analysis.

In Phase III, non inferiority margin will be considered as 0.67 as per WHO guidelines, i.e. non inferiority is demonstrated if the lower bound of 95% CI GMT ratio (GEMCOVAC-OM/ COVISHIELD™) is > 0.67.

- Seroconversion rate is defined as percentage of subjects who will show ≥ 2-fold rise in anti S-IgG (omicron variant) titers at Day 29 compared to baseline.
  Number and percentage of subjects who achieve ≥2- fold rise will be presented by GEMCOVAC-OM and COVISHIELD™ group. 95% CI will be calculated for percentage by using Clopper-Pearson method. Difference between percentages and the 95% CI for the difference will also be calculated by using Meitinen-Nurminen method. Chi-square or Fisher's exact test will be used to compare the subjects who achieve ≥2 fold rise between GEMCOVAC-OM and COVISHIELD™ arms.
  Non-inferiority margin for difference in seroconversion rates is considered as -10% (Seroconversion Rate $_{\text{GEMCOVAC-OM}}$ – Seroconversion Rate $_{\text{COVISHIELD™}}$), i.e. GEMCOVAC-OM vaccine will be considered as non-inferior to the COVISHIELD™ vaccine if the lower bound of 95% CI of the difference in Seroconversion Rates is > -10 %.
- Comparison of neutralizing antibodies against SARS-CoV-2 (cPASS™ assay) at Day 29.
  The median/ mean neutralization percentage at baseline and Days 29 will be evaluated among subjects who receive booster dose. Median/ mean of percentage from pre-vaccination to post-vaccination in each arm will be compared between the subjects receiving GEMCOVAC-OM and who received COVISHIELD™. The analysis will be performed by using analysis of covariance (ANCOVA). The difference in median percentage neutralization will be the outcome variable, Study treatment will be main independent variable and baseline neutralization percentage will be the covariate in the ANCOVA analysis.
- To evaluate cellular immune responses from GEMCOVAC-OM against COVISHIELD™ at Day 29.
  Summary statistics of cell mediated immunity response will be provided by vaccine arms.
- Occurrence and severity of local and systemic reactogenicity adverse events (AEs) for 7 days following vaccination.
- Occurrence of unsolicited adverse events up to day 29 post vaccination
- Occurrence of related unsolicited adverse events up to day 180 post vaccination
- Occurrence of serious adverse events (SAEs): throughout the duration of the study
- Occurrence of adverse events of special interest (AESI): throughout the duration of the study

Adverse events (AE) will be coded in accordance with the MedDRA version 23.0 or higher. Number of Solicited AEs, Unsolicited AEs, Serious AEs and AEs of special interest reported during the study will be presented with number and percentage of subjects who reported the corresponding events by vaccine and comparator arms. Also, 95% confidence interval (CI) for

proportion of subjects will be calculated by using the exact binomial distribution from Clopper-Pearson's method.

Exploratory Endpoint

Phase-II

- To evaluate the immunogenicity detected by IgG ELISA against the SARS-CoV-2 Spike protein with GEMCOVAC-OM dose at Day 90.
- To evaluate the neutralizing antibody (cPASS™ assay) against SARS-CoV-2 post vaccination with GEMCOVAC-OM at Day 90.
- To evaluate cellular immune response from GEMCOVAC-OM at Day 90.

Phase-III

- To evaluate the immunogenicity detected by IgG ELISA against the SARS-CoV-2 Spike protein with GEMCOVAC-OM at Day 90.
- To evaluate the NAb titers against SARS-CoV-2 post vaccination with GEMCOVAC-OM at Day 90.
- To evaluate cellular immune responses GEMCOVAC-OM at Day 90.

6.3 Sample Size Determination

No formal sample size has been calculated for Phase II. The 7 day safety post booster dose will be analyzed and presented to the DSMB. After their favourable opinion, the Phase III will commence.

The Phase III study consists of safety and the immunogenicity cohort. The safety cohort will consist of 3140 participants of which 3000 will receive GEMCOVAC-OM. This was calculated to ensure that a safety database of 3000 participants who received GEMCOVAC-OM is available.

The immunogenicity cohort was calculated for the two primary endpoints

1. Non-inferiority of neutralizing antibody (PRNT) Geometric Mean Titer (GMT) ratio

The immunogenicity cohort was calculated based on the WHO guidelines of non-inferiority defined as lower bound of 95% CI in the neutralizing antibody (PRNT) Geometric Mean Titer (GMT) ratio (GMT in GEMCOVAC-OM / GMT in COVISHIELD™) > 0.67.

The sample size will be allocated in to 2:1 ratio between GEMCOVAC-OM and COVISHIELD™ arms and includes 20% dropout rate.

A sample size of 420 (280 in GEMCOVAC-OM and 140 in COVISHIELD™ arm) in the immunogenicity cohort will provide adequate numbers for the statistical analysis considering a non-inferiority margin of 0.67, standard deviation of 1.82, alpha error of 5% and power of 90%.

2. Non-inferiority of difference in seroresponse rate

The immunogenicity cohort was calculated based on the WHO guideline of non-inferiority defines as lower bound of 95% CI in seroresponse rate difference (Seroconversion Rate $_{\text{GEMCOVAC-OM}}$ - Seroconversion Rate $_{\text{COVISHIELD™}}$) >-10%.

The sample size is allocated in a 2:1 ratio between GEMCOVAC-OM and COVISHIELD™ arms and includes 20% dropout rate.

A sample size of 381 (254 in GEMCOVAC-OM and 127 in COVISHEILD™) in the immunogenicity cohort will provide adequate numbers for the statistical analysis considering a non-inferiority of -10%, alpha error of 5% and power of 90%.

The sample size of 420 (280 in GEMCOVAC-OM and 140 in COVISHIELD™ arm) is considered in this study to provide adequate numbers for the statistical analysis of both the primary endpoints.

6.4 Subject Populations for Analysis

For the statistical analysis, following populations of the subjects will be considered:

Safety Analysis Set: This population will include all the subjects with confirmation of receiving the study vaccine.

Immunogenicity Analysis Set: This population will comprise of all subjects who will receive the study vaccine and for whom valid pre-dosing and at least one post-dosing blood sample will be received for immunogenicity.

7 ADVERSE EVENTS AND SERIOUS ADVERSE EVENTS

7.1 Definition

7.1.1 Adverse Events

An AE is defined as any untoward medical occurrence in a subject, or clinical investigation subject administered a pharmaceutical product, and which does not necessarily have a causal relationship with this treatment. An AE can therefore be any unfavorable and unintended sign, symptom, or disease temporally associated with the use of a study treatment, whether or not related to the study treatment.

Adverse event, which occur, or get worse, should be registered in separate eCRF. Adverse event should be observed from time a subject sign the informed consent form (ICF) till the end of the study. Events observed between informed consent sign off and first dosing, should be specified in the Concomitant Diseases section of the eCRF except for the study conduct related adverse events. An AE will only be recorded if there is a worsening of the preexisting concomitant condition during study conduct with regards to nature, severity, or frequency.

Solicited events are pre-specified and actively monitored during the trial. Solicited events will be recorded during the 7 days period post Study product administration.

Solicited AEs to be collected post booster dose:

**Local solicited AEs**	Pain/ tenderness, Redness/erythema, Induration/swelling, Warmth, Pruritis, Bruising

Systemic solicited AEs	Fever, Headache, Myalgia, Arthralgia, Fatigue, Malaise, Nausea, Chills

Adverse Drug Reaction

An ADR is an untoward and unintended response to a study treatment related to any dose administered. All AEs judged by either the reporting investigator or the sponsor as having a reasonable causal relationship to a medicinal product qualify as ADRs. The expression of "reasonable causal relationship" means to convey in general that there are facts or arguments meant to suggest a causal relationship.

Adverse events will be assessed and classified in accordance with the Division of AIDS (DAIDS) Table for Grading the Severity of Adult and Pediatric Adverse Events #

#Also for Other AEs: Common Terminology Criteria for Adverse Events (CTCAE) criteria version 5.0 can be used.

If it is impossible to assess AE according to the CTCAE v5.0 classification, then five-level AE severity score should be used, which provides for mild, moderate, severe, potentially life-threatening condition and death related to AE corresponding to grades 1 to 5, accordingly. Information about death of subjects (both related and not related to AE) will be collected in the Obituary Notice form. The possibility of AE development should be taken into account during the interview with the subject, screening procedures after signing the informed consent and at every Visit during the study. AE can also be detected during analysis of complaints made by the subject during screening or other doctor's visits as well as during physical examination, laboratory, or instrumental observations.

Each AE should be assessed as much as possible to determine:

- Severity of AEs (Grades 1–5).
- Its duration (start and end date)
- Assessment of the possible AE connection with the study treatment:

Causality Assessment:

For all AEs, sufficient information should be obtained by the Investigator to determine the causality of the AE based on WHO-UMC causality assessment system.[31] The Investigator is required to assess causality of each AE.

An Investigator's causality assessment is the determination of whether there exists a reasonable possibility that the investigational product caused or contributed to an AE. The Investigator must make an assessment of the relationship of each AE (serious and non-serious) to the study treatment(s) and record this relationship in the e-CRF.

Factors that need to be considered when making a causality assessment include temporal relationship, clinical and pathological characteristics of the event(s), pharmacological plausibility, exclusion of confounding factors (medical and medication history), drug interactions, de-challenge / re-challenge, and dose relationship.

A suspected relationship (definite, probable, possible) between the events and the study medication means, in general, that there are facts (evidence) or arguments to suggest a causal relationship. Receipt of additional or clarifying information may warrant reassessment of causality. The Investigator is responsible for assessing relationship of AEs to study treatment in accordance with the following definitions:

Causality term	Assessment criteria
DEFINITE OR CERTAIN	• Event or laboratory test abnormality, with plausible time relationship to drug intake<br>• Cannot be explained by disease or other drugs<br>• Response to withdrawal plausible (pharmacologically, pathologically)<br>• Event definitive pharmacologically or phenomenologically (i.e., an objective and specific medical disorder or a recognized pharmacological phenomenon)<br>• Rechallenge satisfactory, if necessary
PROBABLE/LIKELY	• Event or laboratory test abnormality, with reasonable time relationship to drug intake<br>• Unlikely to be attributed to disease or other drugs<br>• Response to withdrawal clinically reasonable<br>• Rechallenge not required
POSSIBLE	• Event or laboratory test abnormality, with reasonable time relationship to drug intake<br>• Could also be explained by disease or other drugs<br>• Information on drug withdrawal may be lacking or unclear
UNLIKELY	• Event or laboratory test abnormality, with a time to drug intake that makes a relationship improbable (but not impossible)<br>• Disease or other drugs provide plausible explanations
CONDITIONAL/UNCLASSIFIED	• Event or laboratory test abnormality<br>• More data for proper assessment needed, or<br>• Additional data under examination
UNASSESSABLE / UNCLASSIFIABLE	• Report suggesting an adverse reaction

Causality term	Assessment criteria
	• Cannot be judged because information is insufficient or contradictory<br>• Data cannot be supplemented or verified

For SAEs, if the relationship to the study treatment(s) is considered to be unlikely or not related, an alternative suspected etiology should be provided when possible (e.g., concomitant medications, intercurrent illness/events, study-related procedure).

7.1.2 Adverse Events of Special Interest

Adverse events of special interest are noteworthy events for the IP or class of products that a Sponsor may wish to monitor carefully. AESI includes 'Myocarditis'. Typical symptoms of myocarditis are chest pain, shortness of breath and palpitations, which are in addition to myalgia, fever, nausea reported as solicited events. Any subject presenting with chest pain, breathlessness or palpitations will be investigated for event of 'Myocarditis' as per investigator's clinical judgment. Myocarditis will be collected throughout the study.

7.1.3 Recording of COVID 19 Events

COVID-19 will not be considered as adverse event for the study, however COVID-19 cases will be recorded in database independently and will be processed for the applicable endpoint assessments. The subject follow-up will occur till the resolution of the event. However, if any COVID 19 event will lead to hospitalization or further then it will be considered as SAE.

7.1.4 Serious Adverse Events

A serious adverse event is any AE matching any criterion listed below:

- Requires inpatient hospitalization or prolongation of existing hospitalization*

  **(It should be noted that hospital admission of the subject due to planned inpatient treatment because of concomitant diseases existing before enrolment into the study will not be considered as an SAE)*

- Results in death
- Is life-threatening
- It resulted in permanent disability / persistent or significant disability or work incapacity
- Birth of a child with congenital abnormalities / birth defects
- It is medically significant, i.e., it is an event threatening the subject or requiring surgical or therapeutic intervention in order to prevent one of the events specified above

7.2 Procedure for Reporting of Occurrence of Serious Adverse Events

In order to ensure safety of the subject, each SAE, irrespective of a supposed causal relationship with the treatment with the Study Vaccine, should be reported online to the regulatory authority within 24 hours from the moment when such event became known.

Any additional information about SAE related to its complication, course, occurrence of repeated symptoms, relapses should be additionally reported to the regulatory authority online through SUGAM. If the SAE occurred during the other time interval or is considered as non-related to the previous SAE, occurrence of such SAE should be reported separately.

The information about occurrence of all SAEs should recorded in a special form of SAEs occurrence.

Reporting of Serious Adverse Events as per the New drugs and Clinical Trials Rules 2019

All SAEs will be recorded from the time a subject sign the ICF till the end of the study:

- All SAEs, regardless of causal relationship will be reported by the site to Sponsor/ designee, Central Licensing Authority i.e., the Drugs Controller General of India (DCGI), and Institutional Ethics Committee (IEC) of the site.
- Follow-up information on an existing SAE will be reported by the Investigator after the initial report. Where appropriate, hospitalisation or autopsy reports will be made available. All SAEs will be followed up until resolution (i.e., asymptomatic, stabilisation or death).
- The Investigator and Sponsor/Sponsor designee will prepare and submit a detailed analysed report of all the SAEs to the Head of the Institution where the trial is being conducted, to the Chairman of the IEC that accorded approval for conduct of the trial and the Central Licensing Authority within 14 days of knowledge of occurrence of SAE. This notification will be as per the New drugs and Clinical Trials Rules 2019.
- If the SAE resolves after the submission of the 14 days analysed report, the Investigator will prepare and submit a follow up analysed report upon resolution of the SAE.

Serious adverse events must be reported to the study Sponsor/designee. To report such events, a Serious Adverse Event (SAE) Report form must be completed and signed by the investigator or designee and forwarded to:

CRO (JSS Asia Pacific) Details: safety@jssresearch.com

7.3 Pregnancy

In order to ensure safety of the subject, all cases of pregnancy, during which the subject is treated with the Study Vaccine/Comparator Vaccine, shall be reported to the study organizer within 24 hours from the moment when such information becomes known to the study physician. Pregnancy of the subject or female partner of the male study subject should be tracked to its end, at the same time the information about pregnancy course, its termination, particular details of delivery, availability/absence of any defect by a child and any developed complications of a mother and/or child should be gathered.

The information about pregnancy occurrence should be recorded in a special form of a notice on pregnancy occurrence. The subsequent information on the pregnancy should be put in the same form.

Any SAE developed during the pregnancy should be described in a separate form about occurrence of SAE.

8 STUDY PRODUCT MANAGEMENT

8.1 Packaging

The Study product GEMCOVAC-OM and comparator GEMCOVAC-19 is in the pharmaceutical form of Lyophilized powder to be reconstituted with water for injection (WFI). COVISHIELD™ is a liquid vaccine produced by Serum Institute of India.

The study product manufactured by Gennova Biopharmaceuticals Limited will be used in the study.

The CRO will provide all research centers with adequate amounts of Study Vaccine/ Comparator Vaccine. Empty and unused packages of Study Vaccine/ Comparator Vaccine will be kept in the study center for records.

8.2 Blinding of Study Product

This trial is an open-label study: The treatment is known to the subjects, study physicians and data assessment professionals. Blinding is not applivcable.

A Randomization Strategy and IWRS implementation document will detail the elaborative randomization strategy for different subset population such as safety, immunogenicity and cell-mediated immune response indicators.

8.2.1 Receipt of Study Product Supplies

Upon receipt of the study treatment supplies, an inventory must be performed, and a study product receipt log filled out and signed by the person accepting the shipment. It is important that the designated study staff counts and verifies that the shipment contains all the items noted in the shipment inventory. Any damaged or unusable study product in a given shipment (Study Vaccine or Comparator Vaccine) will be documented in the study files. The investigator must notify study Sponsor of any damaged or unusable study treatments that were supplied to the investigator's site.

8.2.2 Storage

Study Vaccine/Comparator Vaccine should be stored at 2 - 8 °C. After reconstitution, the vial can be stored at 2 - 8 °C for 6 hours during which the dose should be administered. After 6 hours of reconstitution, the vial should be discarded.

An improperly used Study Vaccine/Comparator Vaccine shall not be disposed of and shall be kept at the study centre.

The lyophilized GEMCOVAC-OM has shown to be stable up to 6 months at 2 - 8 °C. The lyophilized GEMCOVAC-19 has been shown to be stable upto 9 months at 2 - 8 °C. The use of expired study Study Vaccine/ Comparator Vaccine is prohibited. Expired Study Vaccine/

Comparator Vaccine shall not be disposed of and shall be kept at the study centre and shall be available for counting in case of monitoring visits.

The amount of used and unused Study Vaccine/ Comparator Vaccine shall be counted during in-person monitoring visits to the study centre. The responsible study centre employee shall keep Study Vaccine/ Comparator Vaccine record books, where study products delivery to the study centre, study products usage, unused study products return, as well as storage conditions shall be recorded.

Study Vaccine/Comparator Vaccine shall be stored under the storage conditions specified in the label.

The Study Vaccine/ Comparator Vaccine should be stored in a room/ area that is only accessible to the study centre employees responsible for using the study products.

8.2.3 Dispensing of Study Products

The Study Vaccine/Comparator Vaccine will be administered to all the randomized subjects during randomization Visit 1 (Day 1).

GEMCOVAC-OM will be administered to the subjects via intra-dermal injection.

GEMCOVAC-19 and COVISHIELD™ will be administred intra-muscularly.

At Visit 1 (Day 1) the trial subjects will receive the Study Vaccine/ Comparator Vaccine packed in individual vial/ampoule marked with randomization number.

Regular study product reconciliation will be performed to document study products assigned, study products consumed, and study products remaining. This reconciliation will be logged on the study products reconciliation form and signed and dated by the study team.

8.2.4 Return or Destruction of Study Product

An improperly used Study Vaccine/Comparator Vaccine shall not be disposed of and shall be kept at the study centre. Expired Study Vaccine/ Comparator Vaccine shall not be disposed of and shall be kept at the study centre and shall be available for counting in case of monitoring visits.

The amount of used and unused Study Vaccine/ Comparator Vaccine shall be counted during in-person monitoring visits to the study centre. The responsible study centre employee shall keep study products record books, where study products delivery to the study centre, study products usage, unused study products return, as well as storage conditions shall be recorded. At the completion of the study, there will be a final reconciliation of study products shipped, study products consumed, and study products remaining. This reconciliation will be logged on the product reconciliation form, signed and dated. Any discrepancies noted will be investigated, resolved, and documented prior to return.

9 STUDY ADMINISTRATION

Sponsor's designee JSS- Asia Pacific will be responsible for study administration as described in this section.

9.1 Regulatory and Ethical Considerations, Including the Informed Consent Process

The study will be conducted in conformity with International Council on Harmonization-Good Clinical Practice (ICH-GCP E6(R2)) guidelines, the Helsinki Declaration, and the local regulatory requirements (Indian GCP, Indian Council of Medical Research (ICMR) and New Drugs and Clinical Trials Rules-2019.[32-36]

9.1.1 Institutional Ethics Committee

The study protocol, amendments to the protocol (if applicable), study treatment information, subject information sheet, ICF, and consent form updates (if applicable) and other study specific documents will be submitted to the IEC, which is constituted according to local law to obtain approval before initiation of the study. This study will be initiated after the protocol is reviewed and approved by the concerned IEC. The approval should be kept in the site master file with a copy in the trial master file. The Investigator will report promptly to the IEC any new information that may adversely affect the safety of the subjects or the conduct of the trial.

9.1.2 Informed consent of the subject

The Investigator (or designee) will obtain freely given written, signed, and dated ICF from each subject participating in this clinical trial, after adequate explanation of the aims, methods, anticipated benefits, potential hazards, and any other aspects of the study, and prior to conduct of any study-related screening procedure on the subject. The subject will be given sufficient time to consider the study's implications before deciding to participate in the study.

A copy of signed and dated ICF along with the subject information sheet will be provided to all subjects enrolled in the study. The confidentiality of the subject's identity and records will be maintained. Should there be any amendments to the protocol, such that they would directly affect the subject's study participation (e.g., change in study procedure), the subject information sheet along with the ICF will be also amended appropriately. The Investigator (or designee) must obtain subject's signature on this amended ICF, indicating that they consent to continue their study participation.

9.2 Medical Monitoring

It is the responsibility of the Investigator to oversee the safety of the study at his/her site. This safety monitoring will include careful assessment and appropriate reporting of adverse events as noted above, as well as the construction and implementation of a site data and safety-monitoring plan (see Section 13: Study Monitoring, Auditing, and Inspecting). Medical monitoring will include a regular assessment of the number and type of serious adverse events.

9.3 Unblinding Procedures

This trial is an open-label study: The treatment is known to the subjects, study physicians and data assessment professionals. Unblinding is not applicable.

9.4 Pause Rules

Rule 1: Three or more subjects experience any vaccine-related SAE.

Rule 2: Five or more subjects experience the following Grade 4 local adverse event classified as related to vaccination by the Investigator: ulceration, necrosis, or sterile abscess at the injection site requiring drainage or surgical intervention.

Rule 3: If three or more subjects experience ≥ Grade 3, related unsolicited AE of same preferred term

Rule 4: The Sponsor may use their discretion, ask for the study to be paused or placed on hold for any single event or combination of multiple events which may jeopardize the safety of the subjects or the reliability of the data.

If the Sponsor decide to implement a study pause, the study team will withhold randomization and vaccination temporarily.

Should a study pause be initiated, subjects will continue with their scheduled visits.

The DSMB will decide on restart/stopping of the trial.

In case if the study trial is stopped based on decision by the DSMB members/ Regulatory Authority/ Sponsor, safety monitoring of subjects will be continued for 30 days after the end of study.

10 DATA HANDLING AND RECORD KEEPING

10.1 Confidentiality

Information about subjects will be kept confidential and managed according to the requirements of the ICH-GCP. The anonymity of participating subjects must be maintained. Subjects will be specified on study documents by their subject number, initial or birth date, not by name. Documents that identify the subject (e.g., the signed informed consent document) must be maintained in confidence by the Investigator.

All clinical study findings and documents will be kept as confidential. Study documents (protocols and other material) will be stored appropriately to ensure their confidentiality. The Investigator and members of his/her research team (including the IEC) must not disclose such information without prior written approval from the Sponsor, except to the extent necessary to obtain informed consent from subjects who wish to participate in the study or to comply with regulatory requirements.

If a subject revokes authorization to collect or use protected health information (PHI), the investigator, by regulation, retains the ability to use all information collected prior to the revocation of subject authorization. For subjects that have revoked authorization to collect or use PHI, attempts should be made to obtain permission to collect at least vital status (i.e., that the subject is alive) at the end of their scheduled study period.

10.2 Source Documents

Source data is all information, original records of clinical findings, observations, or other activities in a clinical trial necessary for the reconstruction and evaluation of the trial. Source data are contained in source documents. Examples of these original documents, and data records include: hospital records, clinical and office charts, laboratory notes, memoranda, subjects' diaries or evaluation checklists, pharmacy dispensing records, recorded data from automated instruments, copies or transcriptions certified after verification as being accurate and complete, microfiches, photographic negatives, microfilm or magnetic media, subject files, and records kept at the pharmacy, at the laboratories, and at medico-technical departments involved in the clinical trial.

10.3 Documentation storage

To ensure safe-keeping of the data for further audits by the regulatory authorities, the principal Investigator undertakes to store all the documentation related to the study for the period determined by the applicable regulatory requirements of the country where the study is being conducted as well as the country where the study will be submitted.

The medical investigator undertakes to store all the primary documentation in the way to ensure onsite as well as remote access to it to the CRO representatives, the Sponsor and the auditors from the regulatory authorities and not to prevent from direct access to the primary data/documentation for monitoring, audit, ethical expert examination and inspection by the authorized bodies.

10.4 Case Report Forms

Data reflecting the subject's participation with the study product under investigation will be reported to the Sponsor (or an authorized representative). The data will be recorded on the designated eCRFs provided or approved by the Sponsor.

The eCRF is essentially considered a data entry form and should not constitute the original (or source) medical records unless otherwise specified. Source documents are all documents used by the Investigator or hospital that relate to the subject's medical history, that verify the existence of the subject, the inclusion and exclusion criteria and all records covering the subject's participation in the study. They include laboratory notes, memoranda, pharmacy dispensing records, subject files, etc.

The Investigator is responsible for maintaining source documents. These will be made available for inspection by the study monitor at each monitoring visit. The Investigator must submit a completed Electronic CRF (eCRF) for each subject who did not fail screening. All supportive documentation submitted with the eCRF, such as laboratory or hospital records, should be clearly identified. All data must be entered in English. The eCRFs should always reflect the latest observations on the subjects participating in the trial. Therefore, the eCRFs are to be completed as soon as possible after the subject's eligibility has been confirmed and thereafter during or after the subject's visit. The Investigator must verify that all data entries in the eCRFs are accurate and correct. If some assessments are not done, or if certain information is not available or not applicable or unknown, the Investigator should indicate this in the eCRF. The Investigator will be required to sign off on the clinical data.

The monitor will review the eCRFs and evaluate them for completeness and consistency. The eCRF will be compared with the source documents to ensure that there are no discrepancies between critical data. All entries, corrections and alterations are to be made by the responsible Investigator or his/her designee. The monitor is not allowed to enter data in the eCRFs.

If additional corrections are needed, the responsible monitor or Data Manager will raise a query. The appropriate investigational staff will answer queries sent to the Investigator.

For each subject that has signed the informed consent but does not qualify for allocation to treatment, i.e., Screen Failure, no data is to be collected in the eCRF but captured on a screening log. This includes at a minimum the subject identification number, the informed consent date, and the reason for screen failure. The screening log is monitored, provided to the Sponsor, and must be included in the Trial Master File.

Electronic Data Capture (EDC) will be used for this trial, meaning that all CRF data will be entered in electronic forms at the investigational site. Data collection will be completed by authorized study site personnel designated by the Investigator. Appropriate training and security measures will be completed with the Investigator and all authorized study site personnel prior to the study being initiated and any data being entered into the system for any subjects. Roles and rights of the site personnel responsible for entering the clinical data into the eCRF will be determined in advance.

Once clinical data of the eCRF have been submitted to the central server, corrections to the data fields will be audit trailed, meaning that the reason for change, the name of the person who performed the change, together with time and date will be logged. Also, the queries and resolutions will be audit trailed by the EDC application meaning that the name of investigational staff, Data Manager and Monitor, time and date stamp are captured. eCRF records will be automatically appended with the identification of the creator, by means of their unique User Identification (ID). Specified records will be electronically signed by the Investigator to document his/her review of the data and acknowledgement that the data are accurate. This will be facilitated by means of the Investigator's unique User Identification (ID) and password; date and time stamps will be added automatically at time of electronic signature. If an entry on an eCRF requires change, the correction should be made in accordance with the relevant software procedures. All changes will be fully recorded in a protected audit trail, and a reason for the change will be required.

10.5 Records Retention

All records pertaining to the receipt and return of study supplies (particularly study product) and copies of final case report forms, worksheets, and other pertinent source documents will be retained in accordance with the applicable regulatory requirements of the country where the study is being conducted as well as the country where the study will be submitted.

11 STUDY MONITORING, AUDITING, AND INSPECTING

11.1 Study Monitoring Plan

This study will be monitored according to the SOPs and study's monitoring plan. The Investigator will allocate adequate time for such monitoring activities. The Investigator will also ensure that the monitor or other compliance or quality assurance reviewer is given access to all the above noted study-related documents and study related facilities (e.g., pharmacy, diagnostic laboratory, etc.), and has adequate space to conduct the monitoring visit.

11.2 Auditing and Inspecting

The Investigator will permit study-related monitoring, audits, and inspections by the IEC, the Sponsor, government regulatory bodies, and quality assurance groups of all study related documents (e.g., source documents, regulatory documents, data collection instruments, study data etc.). The Investigator will ensure the capability for inspections of applicable study-related facilities (e.g., pharmacy, diagnostic laboratory, etc.).

Participation as an Investigator in this study implies acceptance of potential inspection by government regulatory authorities, and quality assurance offices.

11.3 Protocol Deviations

The Investigators will not deviate from the protocol without written approval from the Sponsor or the Sponsor's representative and the same will be notified to the Institutional Ethics Committee.

In medical emergencies, the Investigator will use medical judgment and remove the subject from immediate hazard. Any significant changes or deviations in the protocol will be the subject of a protocol amendment and must be pre-approved by the Institutional Ethics Committee.

If an unexpected major[1]/minor[2] deviation from the protocol occurs the Investigator must notify the Sponsor or the Sponsor's representative immediately and the deviation from the protocol will be documented and resolved on a Protocol Deviation Form issued by Sponsor or the Sponsor's representative.

[1]Major deviation is any departure from the protocol which adversely affect the rights and safety of trial subjects, the study outcomes and the integrity of the study.

[2]Minor deviation is any departure from the protocol which adversely does not result in harm to the trial subjects and does not significantly affect the study outcomes or does not adversely affect study integrity.

11.4 Study and Site Closure

Upon completion or premature discontinuation of the study, the monitor will conduct site closure activities with the Principal Investigator or site staff, as appropriate, in accordance with applicable regulations, ICH-GCP guidelines, Sponsor or its designee's procedures.

In addition, Sponsor reserves the right to temporarily suspend or prematurely discontinue this study at any time for reasons including, but not limited to, safety or ethical issues or severe non-compliance. For multi-centre studies, this can occur at one or more or at all sites. If Sponsor determines such action is needed, Sponsor or its designee will discuss this with the Principal Investigator including the reasons for taking such action, at that time. When feasible, Sponsor or its designee will provide advance notification to the Principal Investigator of the impending action

prior to it taking effect. The Principal Investigator also has the right to temporarily suspend or prematurely discontinue this study for mutually agreed reason(s) with the Sponsor.

Sponsor or its designee will also promptly inform the regulatory authorities of the suspension or termination of the study and the reason(s) for the action. The Principal Investigator must inform the IEC/IRB promptly and provide the reason for the suspension or termination, if required by applicable regulations.

12 STUDY FINANCES

12.1 Funding Source

The clinical trial will be funded by Gennova Biopharmaceuticals Ltd.

12.2 Conflict of Interest

Any Investigator who has a conflict of interest with this study (patent ownership, royalties, or financial gain greater than the minimum allowable by their institution, etc.) must have the conflict reviewed by a properly constituted Conflict of Interest Committee with a Committee-sanctioned conflict management plan that has been reviewed and approved by the study Sponsor prior to participation in this study.

13 REPORTS ON STUDY RESULTS

Final report will be in ICH E3 form.

14 PUBLICATION PLAN

The organizer of this study is Gennova Biopharmaceuticals Ltd. The study results can be published or disclosed only after approval of the study organizer.

15 REFERENCES

1. Ge H, Wang X, Yuan X, et al., The epidemiology and clinical information about COVID-19. European Journal of Clinical Microbiology & Infectious Diseases. 2020 Jun;39(6):1011-9.
2. Huang C, Wang Y, Li X, et al., Clinical features of patients infected with 2019 novel coronavirus in Wuhan, China. The lancet. 2020 Feb 15;395(10223):497-506.
3. Chen N, Zhou M, Dong X, et al., Epidemiological and clinical characteristics of 99 cases of 2019 novel coronavirus pneumonia in Wuhan, China: a descriptive study. The lancet. 2020 Feb 15;395(10223):507-13.
4. Wang D, Hu B, Hu C, et al. Clinical characteristics of 138 hospitalized patients with 2019 novel coronavirus-infected pneumonia in Wuhan, China. JAMA. 2020.
5. Updated Clinical Management Protocol for COVID19 dated 03072020. https://www.mohfw.gov.in/pdf/UpdatedClinicalManagementProtocolforCOVID19dated03072020.pdf
6. Guan WJ, Ni ZY, Hu Y, et al. Clinical characteristics of coronavirus disease 2019 in China. N Engl J Med. 2020.
7. Li Q, Guan X, Wu P, et al. Early transmission dynamics in Wuhan, China, of novel coronavirus-infected pneumonia. N Engl J Med. 2020;382(13):1199-1207.
8. Lauer SA, Grantz KH, Bi Q, et al. The incubation period of coronavirus disease 2019 (COVID-19) from publicly reported confirmed cases: estimation and application. Ann Intern Med. 2020.
9. Xu XW, Wu XX, Jiang XG, et al. Clinical findings in a group of patients infected with the 2019 novel coronavirus (SARS-CoV-2) outside of Wuhan, China: retrospective case series. BMJ (Clin Res Ed) 2020;368:m792.
10. Guan WJ, Ni ZY, Hu Y, et al. Clinical characteristics of 2019 novel coronavirus infection in China. MedRxiv. 2020 Jan 1.
11. Wu Z, McGoogan JM. Characteristics of and important lessons from the coronavirus disease 2019 (COVID-19) outbreak in China: summary of a report of 72,314 cases From the Chinese Center for Disease Control and Prevention. JAMA. 2020.
12. Huang C, Wang Y, Li X, et al. Clinical features of patients infected with 2019 novel coronavirus in Wuhan, China. Lancet. 2020;395:497–506.
13. Chen N, Zhou M, Dong X, et al. Epidemiological and clinical characteristics of 99 cases of 2019 novel coronavirus pneumonia in Wuhan, China: a descriptive study. Lancet. 2020;395:507–13.
14. Wang D, Hu B, Hu C, et al. Clinical characteristics of 138 hospitalized patients with 2019 novel coronavirus-infected pneumonia in Wuhan, China. JAMA. 2020.
15. Omari AA, Rabaan AA, Salih S, et al. MERS coronavirus outbreak: implications for emerging viral infections. Diagn Microbiol Infect Dis. 2019;93:265–285.

16. Zhang JJ, Dong X, Cao YY, et al . Clinical characteristics of 140 patients infected by SARS-CoV-2 in Wuhan, China. Allergy. 202010.1111/all.14238
17. Chung M, Bernheim A, Mei X, et al., (2020) CT imaging features of 2019 novel coronavirus (2019-nCoV). Radiology. 10.1148/radiol.2020200230:200230
18. Shi H, Han X, Jiang N, et al. Radiological findings from 81 patients with COVID-19 pneumonia in Wuhan, China: a descriptive study. Lancet Infect Dis. 2020;20(4):425-434.
19. Covid 19 treatment guidelines by NIH. Available at https://www.covid19treatmentguidelines.nih.gov/
20. Clinical Management Protocol_COVID-19. Jun 2020. Ministry of Health & Family Welfare. Directorate General of Health Services. Available at https://www.mohfw.gov.in/pdf/ClinicalManagementProtocolforCOVID19dated27062020.pdf
21. COVID-19 vaccine tracker and landscape (who.int)
22. https://www.cdc.gov/coronavirus/2019-ncov/vaccines/booster-shot.html.
23. Omer SB, Malani PN. Booster Vaccination to Prevent COVID-19 in the Era of Omicron: An Effective Part of a Layered Public Health Approach. JAMA. Published online January 21, 2022. doi:10.1001/jama.2022.0892
24. https://www.cidrap.umn.edu/news-perspective/2022/01/new-data-show-booster-doses-protect-against-omicron
25. https://www.fda.gov/news-events/press-announcements/coronavirus-covid-19-update-fda-takes-additional-actions-use-booster-dose-covid-19-vaccines)
26. Chemaitelly H, Tang P, Hasan MR, et al., Waning of BNT162b2 vaccine protection against SARS-CoV-2 infection in Qatar. New England Journal of Medicine. 2021 Oct 6.
27. Wanwisa Dejnirattisai, Jiandong Huo, Daming Zhou, et al., SARS-CoV-2 Omicron-B.1.1.529 leads to widespread escape from neutralizing antibody responses. 2022, Cell 185, 467–484.
28. Shimabukuro T, Cole M, Su JR . Reports of Anaphylaxis After Receipt of mRNA COVID-19 Vaccines in the US—December 14, 2020-January 18, 2021. JAMA, 2021.
29. Public Health England, Vaccine safety and adverse events following immunisation: The Green Book, Chapter 8. 2020.
30. Liu X, Shaw RH, Stuart ASV, et al., Com-COV Study Group. Safety and immunogenicity of heterologous versus homologous prime-boost schedules with an adenoviral vectored and mRNA COVID-19 vaccine (Com-COV): a single-blind, randomised, non-inferiority trial. Lancet. 2021 Sep 4;398(10303):856-869.
31. https://www.who.int/medicines/areas/quality_safety/safety_efficacy/WHOcausality_assessment.pdf
32. New Drugs & Clinical Trial Rule 2019 Guidelines.

33. Indian GCP guidance
34. ICH (International Council for Hgrouponization) E6 ‘Guideline for Good Clinical Practice’ (Step 4, 2016).
35. Ethical Principles for Medical Research Involving Human Subjects, Declaration of Helsinki (Brazil 2013).
36. National Ethical Guidelines for Biomedical and Health Research involving Human Participants, ICMR (Indian Council of Medical Research, 2017).
37. ICH (International Council for Harmonization) E3 Structure and Contents of Clinical Study Report (Step 4 1994).
38. https://rsc.niaid.nih.gov/sites/default/files/daidsgradingcorrectedv21.pdf

16 APPENDICES

APPENDIX 1. COVID-19 SEVERITY AND SYMPTOMS (MOHFW GUIDELINES)[36]

Clinical Severity	Clinical Presentation	Clinical Parameters
Mild	Patients with uncomplicated upper respiratory tract infection, may have mild symptoms such as fever, cough, sore throat, nasal congestion, malaise, headache	Without evidence of breathlessness or Hypoxia (normal saturation).
Moderate	Pneumonia with no signs of severe disease	Adults with presence of clinical features of dyspnea and or hypoxia, fever, cough, including SpO2 90 to ≤93% on room air, Respiratory Rate more or equal to 24 per minute.
Severe	Severe Pneumonia	Adults with clinical signs of Pneumonia plus one of the following; respiratory rate >30 breaths/min, severe respiratory distress, SpO2 <90% on room air.
	Acute Respiratory Distress Syndrome	Onset: new or worsening respiratory symptoms within one week of known clinical insult.<br>Chest imaging (Chest X ray and portable bed side lung ultrasound): bilateral opacities, not fully explained by effusions, lobar or lung collapse, or nodules.<br>Origin of Pulmonary infiltrates: respiratory failure not fully explained by cardiac failure or fluid overload. Need objective assessment (e.g., echocardiography) to exclude hydrostatic cause of infiltrates/ oedema if no risk factor present.<br>Oxygenation impairment in adults: Mild ARDS: 200 mmHg < PaO2/FiO2 ≤ 300 mmHg (with PEEP or CPAP ≥5 cm H2O) Moderate ARDS: 100 mmHg < PaO2/FiO2 ≤200 mmHg with PEEP ≥5 cm H2O) Severe ARDS: PaO2/FiO2 ≤ 100 mmHg with PEEP ≥5 cm H2O)
	Sepsis	Adults: Acute life-threatening organ dysfunction caused by a dysregulated host response to suspected or proven infection. Signs of organ dysfunction include: altered mental status, difficult or fast breathing, low oxygen saturation, reduced urine output, fast heart rate, weak pulse, cold extremities or low blood pressure, skin mottling, or laboratory evidence of coagulopathy, thrombocytopenia, acidosis, high lactate, or hyperbilirubinemia.
	Septic shock	Adults: persisting hypotension despite volume resuscitation, requiring vasopressors to maintain MAP ≥65 mmHg and serum lactate level > 2 mmol/L.

Note: If in case the MoHFW guideline is revised, then the latest revised MoHFW guideline will be followed.

APPENDIX 2. VACCINATION ADVERSE EVENTS ASSESSMENT SCALE[38]

Sr. No.	Event Name	Mild (Grade 1)	Moderate (Grade 2)	Severe (Grade 3)	Potentially Life Threatening (Grade 4)
**Local Solicited AEs**					
1.	Pain at injection site or Tenderness (Use only one term)	Pain or tenderness causing no or minimal limitation of use of limb	Pain or tenderness causing greater than minimal limitation of use of limb	Pain or tenderness causing inability to perform usual social and functional activities	Pain or tenderness causing inability to perform basic self-care function or hospitalization indicated
2.	Erythema or Redness* (Use only one term)	2.5 to < 5 cm in diameter and Symptoms causing no or minimal interference with usual social and functional activities	≥ 5 to < 10 cm in diameter or Symptoms causing greater than minimal interference with usual social and functional activities	≥ 10 cm in diameter or Ulceration or Secondary infection or Phlebitis or Sterile abscess or Drainage or Symptoms causing inability to perform usual social and functional activities	Potentially life threatening consequences (e.g., abscess, exfoliative dermatitis, necrosis involving dermis or deeper tissue)
3.	Induration or Swelling* (Use only one term)	2.5 to < 5 cm in diameter and Symptoms causing no or minimal interference with usual social and functional activities	≥ 5 to < 10 cm in diameter or Symptoms causing greater than minimal interference with usual social and functional activities	≥ 10 cm in diameter or Ulceration or Secondary infection or Phlebitis or Sterile abscess or Drainage or Symptoms causing inability to perform usual social and functional activities	Potentially life threatening consequences (e.g., abscess, exfoliative dermatitis, necrosis involving dermis or deeper tissue)
4.	Injection site itching (Pruritus)	Itching causing no or minimal interference with usual social & functional activities	Itching causing greater than minimal interference with usual social & functional activities	Itching causing inability to perform usual social & functional activities	NA
5.	Bruising	Localized to one area	Localized to more than one area	Generalized	NA
**Systemic Solicited AEs**					
6.	Fever (non-axillary temperatures only)	38.0 to < 38.6°C or 100.4 to < 101.5°F	≥ 38.6 to < 39.3°C or ≥ 101.5 to < 102.7°F	≥ 39.3 to < 40.0°C or ≥ 102.7 to < 104.0°F	≥ 40.0°C or ≥ 104.0°F
7.	Headache	Symptoms causing no or minimal interference with usual social &	Symptoms causing greater than minimal interference with usual social &	Symptoms causing inability to perform usual social & functional activities	Symptoms causing inability to perform basic self-care functions OR Hospitalization indicated OR

Sr. No.	Event Name	Mild (Grade 1)	Moderate (Grade 2)	Severe (Grade 3)	Potentially Life Threatening (Grade 4)
		functional activities	functional activities		Headache with significant impairment of alertness or other neurologic function
8.	Fatigue	Symptoms causing no or minimal interference with usual social & functional activities	Symptoms causing greater than minimal interference with usual social & functional activities	Symptoms causing inability to perform usual social & functional activities	Incapacitating symptoms of fatigue causing inability to perform basic self-care functions
9.	Chills	Symptoms causing no or minimal interference with usual social & functional activities	Symptoms causing greater than minimal interference with usual social & functional activities	Symptoms causing inability to perform usual social & functional activities	NA
10.	Nausea	Transient (< 24 hours) or intermittent and No or minimal interference with oral intake	Persistent nausea resulting in decreased oral intake for 24 to 48 hours	Persistent nausea resulting in minimal oral intake for > 48 hours or Rehydration indicated (e.g., IV fluids)	Life-threatening consequences (e.g., hypotensive shock)
11.	Myalgia (generalized)	Muscle pain causing no or minimal interference with usual social and functional activities	Muscle pain causing greater than minimal interference with usual social and functional activities	Muscle pain causing inability to perform usual social and functional activities	Disabling muscle pain causing inability to perform basic self-care functions
12.	Joint pain (Arthralgia)	Joint pain causing no or minimal interference with usual social and functional activities	Joint pain causing greater than minimal interference with usual social and functional activities	Joint pain causing inability to perform usual social and functional activities	Disabling joint pain causing inability to perform basic self-care functions
13.	Vomiting	Transient or intermittent AND No or minimal interference with oral intake	Frequent episodes with no or mild dehydration	Persistent vomiting resulting in orthostatic hypotension OR Aggressive rehydration indicated (e.g., IV fluids)	Life-threatening consequences (e.g., hypotensive shock)
14.	Influenza like illness (Flu	Mild flu-like symptoms	Moderate symptoms;	Severe symptoms; limiting	-

Sr. No.	Event Name	Mild (Grade 1)	Moderate (Grade 2)	Severe (Grade 3)	Potentially Life Threatening (Grade 4)
	like symptoms)	present	limiting instrumental activities of daily living (ADL)	self-care ADL	
*Erythema or Redness and Induration or Swelling should be evaluated and graded using the greatest single diameter and if their diameter is less than 2.5 cm then these events will be considered as non-gradable and labelled as Grade 0; temperature >37.5 to <38°C if assessed as clinically significant by Investigator will be considered as Grade 0.<br>Grade 5 indicates death (AEs leading to death)<br>Reference: This Table is derived from DAIDS (Grading the Severity of Adult and Pediatric Adverse Events, corrected version 2.1, July 2017, of the US National Institutes of Health)<br>For 'Warmth', severity grading will be based on Investigator's judgement based on the overall clinical condition of the subject and/or the criteria given in section 9.1.2.1					
