# Supplementary material for: An Omicron-specific, self-amplifying mRNA booster vaccine for COVID-19: a phase 2/3 randomized trial
Source: Nat Med. 2024 Apr 18;30(5):1363–72. doi: 10.1038/s41591-024-02955-2 (PMC11108772; doi:10.1038/s41591-024-02955-2)
Supplement: Supplementary file 4 — Statistical analysis plan for the clinical study. [file 41591_2024_2955_MOESM4_ESM.pdf]

STATISTICAL ANALYSIS PLAN

**Protocol Title :**	A Prospective, Multi-centre, Open-labelled, Randomized, Phase II study seamlessly followed by a Phase III study to evaluate the Safety, Tolerability and Immunogenicity of GEMCOVAC-OM as a booster in Subjects 18 years of age and older.
**Protocol No.:**	GBL/GEMCOVAC-OM/2022/02
**Protocol Date:**	03/10/2022
**SAP Version:**	2.0
**SAP Date:**	12JUL2023

Prepared By:
CRO
JSS Medical Research Asia Pacific Private Limited
Data Management – BIS Tower 2, 1st Floor, South Wing,
L&T Business Park,
Plot no 12/4, Sector 27 D,
Delhi Mathura Road, Near Sarai Khawaja Metro Station,
Faridabad-121003, Haryana, India.
Telephone- (+91) 0129-6613500
Fax-(+91) 01296613520
Sponsor:
Gennova Biopharmaceuticals Ltd.
Hinjawadi Phase II, Rajiv Gandhi Infotech Park,
Hinjawadi, Pune, Maharashtra-411057

Sponsor Signature

I hereby declare that I have reviewed the statistical analysis plan and agree to its form and content. In addition, I confirm that the outlined statistical analysis plan contains all relevant information for the data analysis to be performed in the Protocol No. **GBL/GEMCOVAC-OM/2022/02** study by the Biostatistics Department.

Represented by:

Name	Designation	Signature & Date
Dr. Rohan Gurjar	Assistant General Manager	

Authorization Document

Anjali Yadav	Senior Biostatistician		
Author	Designation	Signature	Date
Chandrashekhar	Team Lead SAS		
Reviewer	Designation	Signature	Date
Dr. Ghazanfar Hussain	General Manager Data Management		
Reviewer	Designation	Signature	Date

Revision History

Version No. of the SAP	Version Date	Author	Description of Modifications from Previous Version
1.0	02JAN2023	Ekta Baranwal	B-Cell Analysis Added

Table of contents

LIST OF ABBREVIATIONS AND DEFINITION OF TERMS 8

1.0 INTRODUCTION 9

2.0 DESCRIPTION OF THE PROTOCOL 9

2.1 Protocol Number and version 9

2.2 Protocol Title 10

2.3 Date 10

2.4 Amendment 10

3.0 STUDY OBJECTIVES AND ENDPOINTS 10

PHASE III 11

4.0 STUDY METHODS 14

4.1 Study Design and Plan 14

4.2 Study Initiation and Completion 17

4.3 Selection of Study Population 18

4.4 Study Subject Group 18

4.5 Study Background 19

4.6 Study Rationale 20

4.7 Schedule of Study Events: 21

4.8 Schedule of Visits and Procedures 23

5.0 GENERAL CONSIDERATIONS FOR STATISTICAL ANALYSIS 24

5.1 Sample Size Determination 24

5.2 Analysis Population 26

5.2.1 Safety population 26

5.2.2 Immunogenicity Analysis Set: 26

5.3 Method of Treatment Assignment, Randomization and Blinding 27

5.4 Baseline 27

5.5 Change from Baseline 28

5.6 End of Study 28

5.7 Treatment Start Day 28

5.8 Treatment End Day 28

5.9 Methods for Withdrawals, Missing Data, and Outliers 28

5.10 Analysis Software 29

6.0 DEMOGRAPHICS AND OTHER BASELINE CHARACTERISTICS 29

6.1 Demographics 29

6.2 Prior and Concomitant Medications 29

6.3 Baseline and Screening Conditions 29

6.3.1 Baseline Medical/Surgical History 30

6.3.2 Baseline Physical Examination and Vital Signs 30

6.3.3 Other Screening Assessments 30

7.0 STATISTICAL ANALYSES 30

7.1 Primary Endpoint Analysis 30

7.2 Secondary Endpoint Analysis 33

7.3 Exploratory Endpoint Analysis 37

8.0 SAFETY AND TOLERABILITY ANALYSES 38

8.1 Adverse Events 38

8.2 Vital Signs 39

8.3 Physical Examination 39

8.4 Pregnancy Test 39

9.0 INTERIM ANALYSIS 40

10.0 REPORTING CONVENTIONS 40

10.1 Reporting of Numeric Values..40

11.0 REFERENCES..41

List of Abbreviations and Definition of Terms

AE	Adverse Event
ANCOVA	Analysis of Co-Variance
BMI	Body Mass Index
CI	Confidence Interval
COVID-19	Coronavirus disease
CRF	Case Report Form
CRO	Contract Research Organization
eCFR	Electronic Case Report Form
ELISA	Enzyme-Linked Immunosorbent Assay
GMT	Geometric Mean Titre
ICF	Informed Consent Form
ICH-GCP	International Council on Harmonization-Good Clinical Practice
IgG	Immunoglobulin G
IgM	Immunoglobulin M
IWRS	Interactive Web Response System
MedDRA	Medical Dictionary for Regulatory Activities
mRNA	Messenger Ribonucleic Acid
NAbs	Neutralizing Antibodies
PBMC	Peripheral Blood Mononuclear Cells
PRNT	Plaque Reduction Neutralization Test
PT	Preferred Term
RNA	Ribonucleic Acid
RT-PCR	Reverse Transcriptase-Polymerase Chain Reaction
SAE	Serious Adverse Event
SAP	Statistical Analysis Plan
SARS-CoV-2	Severe Acute Respiratory Syndrome Coronavirus-2
SAS	Statistical Analysis System
SD	Standard Deviation
TLGs	Tables, listings, and graphs
WHO	World Health Organization

1.0 INTRODUCTION

This Statistical Analysis Plan (SAP) describes a comprehensive and detailed description of the strategy and statistical technique to be used for the analysis of data for protocol GBL/GEMCOVAC-OM/2022/02, entitled 'A Prospective, Multi-centre, Open-labelled, Randomized, Phase II study seamlessly followed by a Phase III study to evaluate the Safety, Tolerability and Immunogenicity of GEMCOVAC-OM as a booster in Subjects 18 years of age and older'.

Phase II and III parallel-arm, multicentre clinical studies are being completed to assess the Safety, Tolerability and Immunogenicity of GEMCOVAC-OM as a booster in Subjects 18 years of age and older.

The reader of this SAP is encouraged to also read the clinical protocols for details on the conduct of this study and the operational aspects of clinical assessments and timing for completing a participant enrollment in this study. The purpose of this SAP is to outline the planned analyses to be completed to support the completion of the Clinical Study Report (CSR) for protocol GBL/GEMCOVAC-OM/2022/02. The planned analyses identified in this SAP will be included in regulatory submissions if applicable and/or future manuscripts. In addition, exploratory analyses not necessarily identified in this SAP may be performed to support the clinical development program. Any post-hoc or unplanned analyses which is not identified in this SAP and performed will be clearly identified in the respective CSR.

2.0 DESCRIPTION OF THE PROTOCOL

2.1 Protocol Number and version

GBL/GEMCOVAC-OM/2022/02 and version 4.0

2.2 Protocol Title

A Prospective, Multi-centre, Open-labelled, Randomized, Phase II study seamlessly followed by a Phase III study to evaluate the Safety, Tolerability and Immunogenicity of GEMCOVAC-OM as a booster in Subjects 18 years of age and older.

2.3 Date

03 OCT 2022

2.4 Amendment

Protocol version 3.0. 29 Sept 2022, Protocol version 2.0., Protocol version 1.0.

3.0 STUDY OBJECTIVES AND ENDPOINTS

OBJECTIVES	ENDPOINTS
**Primary**	**Primary endpoints include:**
1. To assess the safety of adult subjects who received GEMCOVAC-OM as a booster dose till Day 180	• Occurrence and severity of local and systemic reactogenicity adverse events (AEs) for 7 days following vaccination.<br>• Occurrence of unsolicited adverse events up to day 29 post vaccination.<br>• Occurrence of related unsolicited adverse events throughout the duration of the study.<br>• Occurrence of serious adverse events (SAEs): throughout the duration of the study.
2. To evaluate the immunogenicity as detected by Immunoglobulin G- Enzyme-linked immunosorbent assay (IgG ELISA) against the SARS-CoV-2 Spike protein of GEMCOVAC-OM in comparison with GEMCOVAC-19 at Day 29	• Comparison of anti-Spike (omicron variant) IgG Antibodies (GMT) at Day 29 with GEMCOVAC-OM against GEMCOVAC-19
**Secondary**	**Secondary endpoints include:**

OBJECTIVES	ENDPOINTS
1. To evaluate the immunogenicity as detected by IgG ELISA against the SARS-CoV-2 Spike protein of GEMCOVAC-OM in comparison with GEMCOVAC-19 at Day 29	• Comparison of seroconversion rates as assessed by ≥ 2- fold rise in anti-Spike IgG antibodies at Day 29
2. To evaluate the NAb against SARS-CoV-2 post vaccination with GEMCOVAC-OM in comparison with GEMCOVAC-19 at Day 29.	• Comparison of neutralizing antibodies against SARS-CoV-2 using a surrogate virus assay (cPASS™ neutralization antibody kit) at Day 29
3. To evaluate cellular immune response from GEMCOVAC-OM in comparison with GEMCOVAC-19 at Day 29	• Cell mediated immunity assessment by cytokine expression from stimulated PBMCs at Day 29 (20% of participants)
**Exploratory**	**Exploratory endpoint include:**
1. To evaluate the immunogenicity detected by IgG ELISA against the SARS-CoV-2 Spike protein of GEMCOVAC-OM at Day 90	• GMT measured by IgG-ELISA against SARS-CoV-2 Spike protein (omicron variant) post booster administration at Day 90
2. To evaluate the NAb against SARS-CoV-2 post vaccination with GEMCOVAC-OM at Day 90	• Neutralisation antibodies against SARS-CoV-2 using a surrogate virus assay (cPASS™ neutralization antibody kit) post booster administration at Day 90
3. To evaluate the cellular immune response from GEMCOVAC-OM at Day 90	• Assessment of cellular immune responses from stimulated PBMCs at Day 90 (20% of participants)
4. To assess symptomatic COVID-19 events till end of the study	• Symptomatic laboratory confirmed COVID-19 cases throughout the duration of the study

Phase III

OBJECTIVES	ENDPOINTS
**Primary**	**Primary endpoints include:**
1. To evaluate the neutralizing antibody (NAb) titers against SARS-CoV-2 post vaccination with GEMCOVAC-OM in comparison with COVISHIELD™ at Day 29	• Comparison of neutralizing antibody titers against SARS-CoV-2 (omicron variant) using plaque reduction neutralization test (PRNT) assay with COVISHIELD™ at Day 29 by non-inferiority<br>• Comparison of seroconversion rates as assessed by ≥ 2- fold rise in neutralizing

OBJECTIVES	ENDPOINTS
	antibodies against SARS-CoV-2 (omicron variant) using PRNT at Day 29 between GEMCOVAC-OM and COVISHIELD™ by non-inferiority
**Secondary**	**Secondary endpoints include:**
1. To assess the safety of adult subjects who received GEMCOVAC-OM as booster dose till Day 180	• Occurrence and severity of local and systemic reactogenicity adverse events (AEs) for 7 days following vaccination<br>• Occurrence of unsolicited adverse events up to day 29 post vaccination<br>• Occurrence of related unsolicited adverse events throughout the duration of the study<br>• Occurrence of serious adverse events (SAEs): throughout the duration of the study
2. To evaluate the immunogenicity as detected by IgG ELISA against the SARS-CoV-2 Spike protein with GEMCOVAC-OM in comparison with COVISHIELD™ at Day 29	• Comparion of anti-Spike (omicron variant) IgG antibodies (GMT) between GEMCOVAC-OM and COVISHIELD™ at Day 29<br>• Comparison of seroconversion rates as assessed by ≥ 2- fold rise in antibody titers at Day 29 between GEMCOVAC-OM and COVISHIELD™ using test of non-inferiority
3. To evaluate the NAb against SARS-CoV-2 post vaccination with GEMCOVAC-OM in comparison with COVISHIELD™ at Day 29	• Comparison of neutralizing antibodies against SARS-CoV-2 using a surrogate virus assay (cPASS™) at Day 29
4. To evaluate the cellular immune response from GEMCOVAC-OM in comparison with COVISHIELD™ at Day 29	• Cell mediated immunity assessment by cytokine expression from stimulated PBMCs at Day 29 (25% of participants)
**Exploratory**	**Exploratory endpoint include:**
1. To evaluate the immunogenicity detected by IgG ELISA against the SARS-CoV-2 Spike protein of GEMCOVAC-OM at Day 90	• GMT measured by IgG-ELISA against SARS-CoV-2 Spike (omicron variant) protein post booster administration at Day 90

OBJECTIVES	ENDPOINTS
2. To evaluate the NAb against SARS-CoV-2 post vaccination with GEMCOVAC-OM at Day 90	• Neutralisation antibodies against SARS-CoV-2 using a surrogate virus assay (cPASS™ neutralization antibody kit) post booster administration at Day 90<br>• GMT of SARS-CoV-2 specific serum neutralizing antibody levels using live virus (PRNT) assay, at Day 90
3. To evaluate the cellular immune response GEMCOVAC-OM at Day 90	• Assessment of cellular immune responses from stimulated PBMCs at Day 90 (25% of participants)
4. To evaluate COVID-19 infections	• Symptomatic Laboratory confirmed COVID-19 cases throughout the duration of the study

4.0 STUDY METHODS

4.1 Study Design and Plan

This is a Phase II seamlessly followed by Phase III study to evaluate the immunogenicity and safety of a booster dose of the Omicron-specific mRNA vaccine – GEMCOVAC-OM. The study participants will be adult subjects who are fully vaccinated against COVID-19 with either COVAXIN™ or COVISHIELD™ and received last dose of primary vaccination at least 4 months prior to screening. This booster dose of GEMCOVAC-OM will be based on the sequence of Omicron variant BA.1 of SARS-CoV-2. The study will be conducted in two parts: Phase II part will be conducted in 140 subjects while Phase III part of the study will enrol 3140 subjects.

Phase II

Approximately 140 subjects will be randomized, and the enrolment will be competitive. Hence, the subjects will be randomized in 1:1 ratio into two arms:

<u>Arm I:</u> 70 Subjects who have received either COVAXIN™ or COVISHIELD™ as primary vaccination (both doses) will receive a booster dose of GEMCOVAC-OM (intra-dermal).

<u>Arm II</u>: 70 Subjects who have received either COVAXIN™ or COVISHIELD™ as primary vaccination (both doses) will receive a booster dose of GEMCOVAC-19 (Intramuscular)

It will be ensured that at least 20% (n ~14) of the participants in each arm will have received COVAXIN™ and COVISHIELD™ as their primary vaccination.

All the subjects randomized in Arm I will receive 1 dose of GEMCOVAC-OM and those who are randomized in Arm II will receive 1 dose of GEMCOVAC-19 (Intramuscular) on Day 1. GEMCOVAC-OM will be administered intradermally by PharmaJet Tropis Needle-Free Injector.

Phase III

Approximately 3140 subjects will be randomized, and the enrolment will be competitive. The subjects will be randomized into two arms:

Arm I: 3000 Subjects who have received either COVAXIN™ or COVISHIELD™ as primary vaccination (both doses) will receive a booster dose of GEMCOVAC-OM

Arm II: 140 Subjects who have received COVISHIELD™ as primary vaccination (both doses) will receive a booster dose of COVISHIELD™

All the subjects randomized in Arm I will receive 1 dose of GEMCOVAC-OM and subjects randomized in Arm II will receive 1 dose of COVISHIELD™.

The immunogenicity subset will include 280 subjects from Arm I and all the subjects from Arm II (n=140). The dose of GEMCOVAC-OM will be administered by PharmaJet Tropis Needle-Free Injector.

In the immunogenicity cohort of Arm I, it will be ensured that a minimum of 42 participants (15%) will have receieved COVAXIN™ and COVISHIELD™ as their primary vaccination.

In the safety cohort of Arm I, a minimum of 300 participants (10%) will receive COVAXIN™ and COVISHIELD™.

Each subject must agree to participate in screening procedures by signing the most recent Ethics Committee approved Informed Consent Form (ICF) before any screening procedure is initiated. Each subject will be assigned a unique screening number on first cum first basis. Subjects satisfying the inclusion and none of the exclusion criteria will be randomized in the study.

Subjects who qualify screening assessments will be randomized in the study and will receive single dose of booster vaccine on Visit 1 (Day 1).

Subjects will be assessed for infection risk category at screening. Physical examination/ Assessment of the main vital signs will be performed at all onsite visits. /SAEs will be recorded throughout the study. Urine pregnancy test will be performed at screening for female of childbearing potential.

The subjects will have post vaccination telephonic follow-up on Day 7.

A thermometer will be provided to all the randomized subjects, and they will be instructed to record their daily body temperature, for a period of 1 week after booster dose administration/randomization in e-diary/paper diary, wherever usage of e-diary is not feasible.

Immunogenicity assessments will be performed in all the randomized subjects as per schedule of events.

All subjects receiving booster dose will participate in this clinical trial for approximately 6 months (Day 180+14 days) after the administration of the vaccine. The participants will have one screening/ vaccination visit and three on-site follow- up visits during the trial period.

The sites will collect the subject data using appropriate case report forms. Institutional Review Board/Institutional Ethics Committee approval will be taken prior to study initiation.

Study procedures will be as per assessment schedule.

Study Flow Chart

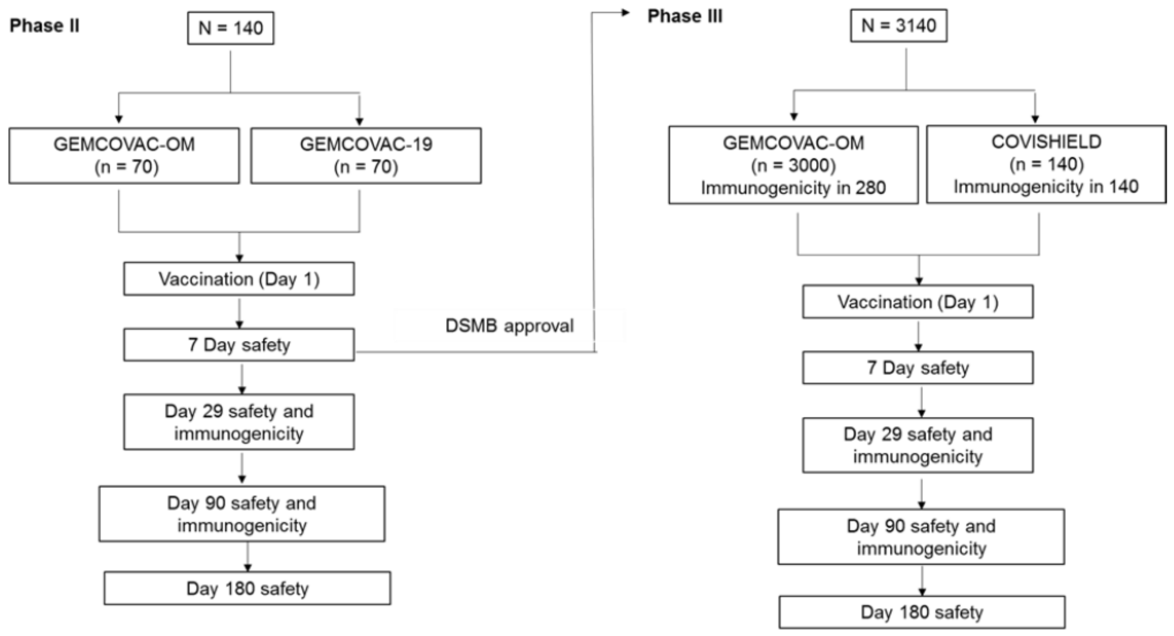

In Phase II, participants who have received 2 doses of either COVAXIN™ or COVISHIELD™ will be enrolled in both the arms. It will be ensured that at least 20% (n ~14) of the participants in each arm will have received COVAXIN™ and COVISHIELD™ as their primary vaccination.

In Phase III, participants who received 2 doses of either COVAXIN™ or COVISHIELD™ will be enrolled in the GEMCOVAC-OM booster arm. Whereas, in the COVISHIELD™ booster arm, participants who have received 2 doses of COVISHIELD™ only will be enrolled. In the immunogenicity cohort of Arm I, a minimum of 42 participants (15%) will have received COVAXIN™ and COVISHIELD™. In the safety cohort of Arm I, a minimum of 300 participants (10%) will have received COVAXIN™ and COVISHIELD™ as their primary vaccination.

4.2 Study Initiation and Completion

Approximate duration of the study is 6 months (Day 180+14 days).

All subjects receiving GEMCOVAC-19 will participate in this clinical trial for approximately 6 months (Day 180+14 days) after the administration of the vaccine. The participants will be administered as a single dose on Day 1. Vaccinated subjects included in the study will be followed up to approximately 6 months (180+ 14 days) of study period thereafter.

4.3 Selection of Study Population

Subjects who meet all the inclusion criteria and none of the exclusion criteria, will be randomized in the study. The study participants will be adult subjects who are fully vaccinated against COVID-19 with either COVAXIN™ or COVISHIELD™ and have received last dose of primary vaccination at least 4 months prior to screening. Each subject will be evaluated from the day of signing of informed consent form till the End of Study.

4.4 Study Subject Group

Phase II

Approximately 140 subjects will be randomized, and the enrolment will be competitive.

Hence, the subjects will be randomized in 1:1 ratio into two arms:

<u>Arm I:</u> 70 Subjects who have received either COVAXIN™ or COVISHIELD™ as primary vaccination (both doses) will receive a booster dose of GEMCOVAC-OM (intra-dermal).

<u>Arm II:</u> 70 Subjects who have received either COVAXIN™ or COVISHIELD™ as primary vaccination (both doses) will receive a booster dose of GEMCOVAC-19 (Intramuscular)

It will be ensured that at least 20% (n ~14) of the participants in each arm will have received COVAXIN™ and COVISHIELD™ as their primary vaccination.

All the subjects randomized in Arm I will receive 1 dose of GEMCOVAC-OM and those who are randomized in Arm II will receive 1 dose of GEMCOVAC-19 (Intramuscular) on Day 1. GEMCOVAC-OM will be administered intradermally by PharmaJet Tropis Needle-Free Injector.

Phase III

Approximately 3140 subjects will be randomized, and the enrolment will be competitive. The subjects will be randomized into two arms:

Arm I: 3000 Subjects who have received either COVAXIN™ or COVISHIELD™ as primary vaccination (both doses) will receive a booster dose of GEMCOVAC-OM

Arm II: 140 Subjects who have received COVISHIELD™ as primary vaccination (both doses) will receive a booster dose of COVISHIELD™

All the subjects randomized in Arm I will receive 1 dose of GEMCOVAC-OM and subjects randomized in Arm II will receive 1 dose of COVISHIELD™.

The immunogenicity subset will include 280 subjects from Arm I and all the subjects from Arm II (n=140). The dose of GEMCOVAC-OM will be administered by PharmaJet Tropis Needle-Free Injector.

In the immunogenicity cohort of Arm I, it will be ensured that a minimum of 42 participants (15%) will have received COVAXIN™ and COVISHIELD™ as their primary vaccination.

In the safety cohort of Arm I, a minimum of 300 participants (10%) will have receieved COVAXIN™ and COVISHIELD™ as their primary vaccination.

Each subject must agree to participate in screening procedures by signing the most recent Ethics Committee approved Informed Consent Form (ICF) before any screening procedure is initiated. Each subject will be assigned a unique screening number on first cum first basis. Subjects satisfying the inclusion and none of the exclusion criteria will be randomized in the study.

Subjects who qualify screening assessments will be randomized in the study and will receive single dose of booster vaccine on Visit 1 (Day 1).

4.5 Study Background

After getting vaccinated against COVID-19, protection against the virus may be reduced due to decrease in neutralizing antibody levels over time. Moreover, the new variants such as Omicron have shown to evade antibody recognition resulting in reduced efficacy of the vaccine. The health officials from USA have taken the decision to encourage coronavirus booster shots, after reviewing data showing that vaccine-produced immunity to milder infection decreases over time.

4.6 Study Rationale

Rationale for booster dose

After getting vaccinated against COVID-19, protection against the virus may be reduced due to decrease in neutralizing antibody levels over time. Moreover, the new variants such as omicron have been shown to evade antibody recognition resulting in reduced efficacy of the vaccine. Emergence of highly transmissible variants of SARS-CoV-2 like omicron has led to considerations for booster doses to enhance immunity and provide sustained protection from COVID-19. [1] Emerging evidence shows that among healthcare and other frontline workers, vaccine effectiveness against COVID-19 decreases rapidly due to the combination of waning immunity and the greater exposure to virus including variants.

A clinical trial involving booster shot of BNT162b2 (Pfizer's mRNA-based vaccine) showed an increase in the immune response in trial participants, who had completed primary vaccination 6 months before. [1]

The officials involved in public health from USA have taken the decision to encourage coronavirus booster shots, after reviewing data showing that vaccine-produced immunity to milder infection decreases over time. Recent findings from Israel and Qatar [2] reported an increasing proportion of breakthrough cases among the earliest vaccinated individuals. The transmissible delta variant and the observed waning protection against symptomatic infection with time since vaccination justify the need for a booster dose in healthy adult subjects as a booster dose can dramatically increase the amount of circulating antibodies. [3]

US- FDA has already authorized Omicron-specific booster doses of Pfizer and Moderna vaccines to certain high-risk adults, 6 months after the primary vaccination. US FDA has also authorized use of a single booster dose of the Janssen COVID-19 vaccine that may be administered at least 2 months after completion of the single-dose primary regimen to individuals 18 years of age and older. US-FDA has also allowed “mix and match” vaccines i.e., getting a booster shot of a different vaccine than the one individuals received as their primary vaccines. [4]

In light of the above data and recommendations for variant specific booster doses by WHO and different regulatory authorities, a prospective, multi-centre, open-labelled, randomized, phase II study seamlessly followed by a Phase III study to evaluate the safety, tolerability and immunogenicity of GEMCOVAC-OM as a booster dose is proposed to be conducted in subjects 18 years of age and older.

Rationale of comparator

In the Phase II study, the safety and immunogenicity of GEMCOVAC-OM is being compared to the prototype vaccine GEMCOVAC-19. GEMCOVAC-19 has received emergency use authorization and safety data has already been generated using this vaccine. GEMCOVAC-OM has been designed using the sequence of the omicron variant of the SARS-COV-2.

In Phase III study, COVISHIELD™ will be used as a comparator. COVISHIELD™ has been approved as a precautionary third dose in India in participants who have received COVISHIELD™ as the primary vaccine. A study has been published that has shown that a third dose of COVISHIELD™ increases the neutralization against all variants of SARS-CoV-2 including omicron. [5] This makes COVISHIELD™ an ideal comparator.

4.7 Schedule of Study Events:

The schedule of study events shall be as follows:

- Visit 1- (Screening Visit/ Baseline/ Randomization Visit & Booster dose administration) - Day 1
- Telephonic Visit (Safety) [Day 7 + 3].
- Visit 2- (Safety & Immunogenicity) - Day 29 + 7 – in-person visit
- Visit 3- (Safety & Immunogenicity) - Day 90 + 14– in-person visit.
- Visit 4- (Safety) - Day 180 + 14 – in-person visit (EOS)

Unscheduled Visits

Any unscheduled visits must be recorded in the source documents and the electronic case report form. Such unscheduled visits should not affect the schedule of regular visits stipulated by the study protocol.

4.8 Schedule of Visits and Procedures

Table 1 Schedule of Visits (Phase II & Phase III)

Visit schedule	V1 (Screening Visit/ Baseline/Randomization Visit & Booster dose administration)	TC (Safety)	V2 (Safety & Immunogenicity)	V3 (Safety & Immunogenicity)	V4 (Safety)
	Day 1	Day 7 + 3	Day 29+7 – in-person visit	Day 90+14– in-person visit	Day 180+14 – in-person visit (EOS)
Informed consent	X				
Evaluation of inclusion/ exclusion criteria, taking the history, demographic[1] and anthropometric data	X				
Assessment of infection risk category[$]	X				
RT-PCR testing for COVID-19[@]	X				
Randomization	X				
Physical examination[2]/ Assessment of the main vital signs[3]	X		X	X	X
Urine pregnancy test[4]	X				
Study Product administration[5]	X				
Subjects filling out diaries[6]	X	X			
Concomitant Medications	X	X	X	X	X

Visit schedule	V1 (Screening Visit/ Baseline/Randomization Visit & Booster dose administration)	TC (Safety)	V2 (Safety & Immunogenicity)	V3 (Safety & Immunogenicity)	V4 (Safety)
	Day 1	Day 7 + 3	Day 29+7 – in-person visit	Day 90+14– in-person visit	Day 180+14 – in-person visit (EOS)
AE/SAE's reporting	X	X	X	X	X
Post booster dose administration/randomization telephonic follow-up		X			
Assesment of Neutralizing Antibodies	X#		X	X	
Evaluation of Anti-S IgG Antibodies	X#		X	X	
Cellular Immunity	X#		X	X	

AEs=adverse events; COVID-19=Coronavirus 2019; EOS=End of Study; IC=informed consent; RT-PCR=Reverse transcriptase Polymerase Chain Reaction; SAEs=serious adverse events; V=Visit;.TC= Telephonic Consultation

[1]Demographics include Date of Birth or age, height, weight, and Sex.

[2]Physical examination includes assessment of the following body parameters: condition of skin, locomotive system, gastrointestinal tract, respiratory organs, cardiovascular system, urogenital system. Examination of lymph nodes (submandibular, cervical, cubital, inguinal lymph nodes) by touch should include assessment of their size, consistency, tenderness, mobility, matting with each other and with surrounding tissues and skin as per the investigator's discretion.

[3]Vital signs include Body temperature, Heart rate, Respiratory rate, systolic and diastolic blood pressure.

[4]Urine pregnancy test will be performed and if positive then Serum pregnancy test will be done for confirmation.

Blood sampling will be performed on day 1 after the subject is randomized to either 'booster arm' or 'comparator arm'. The sampling is to be performed immediately prior to the Study Product administration

[5] a)For subjects receiving the booster dose or comparator vaccine, If the body temperature is higher than 37.0°C on the day of administration of booster dose, the randomization visit can be postponed to the following day. Postponing the administration of booster dose for medical reasons (e.g., due to fever, common cold, diarrheal disease) will not be considered a deviation from the trial protocol.

b) GEMCOVAC-OM will be administered by PharmaJet Tropis Needle-Free Injector.

[6]All subjects must complete diary till Day 7 post booster dose administration, as well as when they feel unwell or when their condition deteriorates

5.0 GENERAL CONSIDERATIONS FOR STATISTICAL ANALYSIS

The statistical analysis will be performed using the SAS version 9.4. The descriptive statistics for the continuous data will be presented using mean, standard deviation, median, minimum, maximum and range, whereas, for the categorical data it will be presented using number of observations (n) and percentages (%). For categorical data, the descriptive statistics will be presented with number of exposed subjects and number (n) with percentage of observations in various categories of the endpoint, where percentage will be based on the exposed subjects. Descriptive analyses will also include graphical presentations of data wherever appropriate. Individual data listings will be also provided.

5.1 Sample Size Determination

No formal sample size has been calculated for Phase II. The 7-day safety post booster dose will be analyzed and presented to the DSMB. After their favourable opinion, the Phase III will commence.

The Phase III study consists of safety and the immunogenicity cohort. The safety cohort will consist of 3140 participants of which 3000 will receive GEMCOVAC-OM. This was calculated to ensure that a safety database of 3000 participants who received GEMCOVAC-OM is available.

The immunogenicity cohort was calculated for the two primary endpoints.

1. Non-inferiority of neutralizing antibody (PRNT) Geometric Mean Titer (GMT) ratio

The immunogenicity cohort was calculated based on the WHO guidelines of non-inferiority defined as lower bound of 95% CI in the neutralizing antibody (PRNT) Geometric Mean Titer (GMT) ratio (GMT in GEMCOVAC-OM / GMT in COVISHIELD™) > 0.67.

The sample size will be allocated in to 2:1 ratio between GEMCOVAC-OM and COVISHIELD™ arms and includes 20% dropout rate.

A sample size of 420 (280 in GEMCOVAC-OM and 140 in COVISHIELD™ arm) in the immunogenicity cohort will provide adequate numbers for the statistical analysis considering a non-inferiority margin of 0.67, standard deviation of 1.82, alpha error of 5% and power of 90%.

	Case 1	Case 2	Case 3	Case 4
Mean difference b/w Test vs Reference	5%	5%	5%	5%
Non-Inferiority margin	0.67	0.67	0.67	0.67
Standerd Deviation	1.82	1.82	1.82	1.82
Power (1- beta) %	0.8	0.9	0.95	0.99
Alpha error (%)	0.05	0.05	0.05	0.05
Ntotal (2:1)	243	336	423	615
Required sample (2:1) with 20 % Dropout	304	420	529	769

2. Non-inferiority of difference in seroresponse rate

The immunogenicity cohort was calculated based on the WHO guideline of non-inferiority defines as lower bound of 95% CI in seroresponse rate difference (Seroconversion Rate GEMCOVAC-OM - Seroconversion Rate COVISHIELD™) >-10%.

The sample size is allocated in a 2:1 ratio between GEMCOVAC-OM and COVISHIELD™ arms and includes 20% dropout rate.

A sample size of 381 (254 in GEMCOVAC-OM and 127 in COVISHEILD™) in the immunogenicity cohort will provide adequate numbers for the statistical analysis considering a non-inferiority of -10%, alpha error of 5% and power of 90%.

	Case 1	Case 2	Case 3	Case 4
Proportion difference b/w test vs reference	-2%	-2%	-2%	-2%
Non-Inferiority margin	-10%	-10%	-10%	-10%
Power (1- beta) %	0.8	0.9	0.95	0.99
Alpha error (%)	0.025	0.025	0.025	0.025
Ntotal (2:1)	219	303	384	561
Required sample (2:1) with 20 % Dropout	274	379	480	701

The sample size of 420 (280 in GEMCOVAC-OM and 140 in COVISHIELD™ arm) is considered in this study to provide adequate numbers for the statistical analysis of both the primary endpoints.

5.2 Analysis Population

For the statistical analysis, following populations of the subjects will be considered:

5.2.1 Safety population

This population will include all the subjects with confirmation of receiving the study vaccine (Answer to 'Was IP administered:'=Yes').

5.2.2 Immunogenicity Analysis Set:

Full analysis set (FAS):

FAS population includes subjects in the safety population who received the planned vaccine dose and have pre- vaccination (Prior to Dose 1) and Day 29 post Dose 1 (Visit 2) immunogenicity measurement(s) available for analysis. FAS is based on ITT (intention to treat analysis) principle which includes all subjects randomized in the study.

Per-protocol (PP) Population set:

PP population includes all subjects in FAS population who received the vaccine dose of study vaccines as per the assigned treatment group and have immunogenicity measurement(s) at pre- vaccination (Prior to Dose 1) and Day 29 post Dose 1 (Visit 2) with no major protocol

deviations that were determined to potentially interfere with immune response to the study vaccine.

This population will serve as the primary population for the immunogenicity objectives for testing non-inferiority hypotheses.

This population will also be used for the immunogenicity analysis related to secondary objectives along with FAS population.

5.3 Method of Treatment Assignment, Randomization and Blinding

Subjects who meet the inclusion/exclusion criteria and have successfully completed all screening procedures will be randomized in 1:1 ratio into two arms for phase II (70 Subjects each) and in two arms for phase III (Arm I: 3000 Subjects and Arm II: 140 Subjects), through block randomization. This is an open-labelled study i.e., a study where designated investigator site staff will remain unblinded to the study drug. The treatment is known to the subjects, the investigator and the independent biostatistician. Subjects who have received COVAXIN™/COVISHIELD™ as primary vaccination (both doses) will receive either Study Vaccine/Comparator Vaccine.

Unique randomization codes will be assigned to the subjects and will remain unchanged until the completion of the trial. The randomization codes will be generated through Proc Plan using SAS® version 9.4 or higher (SAS Institute Inc, Cary, North Carolina) by an independent biostatistician.

The final randomization list will be kept strictly confidential, filed securely by the independent biostatistician, and accessible only to authorized persons per Sponsor (or designee) standard operating procedures until completion of the study.

5.4 Baseline

Baseline value will be defined /as the last non-missing assessment prior to the first study treatment dose.

In this study, the Baseline Visit is Visit 1/Day 1.

5.5 Change from Baseline

Value of change from baseline at any post baseline visit will be defined as the difference of the non-missing baseline value to the non-missing post baseline value i.e.

Change from baseline (Δ) = post-baseline value at visit X - baseline value, where both values are non-missing.

Percent change from baseline will be calculated as:

(Assessment value at post-baseline visit X – baseline value) / baseline value * 100.

5.6 End of Study

End of study value is defined as the last non-missing assessment at the end of study treatment visit. In this study, the end of visit is Visit 4/Day 180 + 14 in-person visit.

If the subject has not completed the study, then the last available non-missing value prior to the end of study treatment visit will be used as end of study value and that visit will be considered as early termination visit.

5.7 Treatment Start Day

The vaccine will be administered at Visit 1/Day 1, which will be the treatment start day.

5.8 Treatment End Day

As the vaccine will be administered only once, treatment end day is not applicable.

5.9 Methods for Withdrawals, Missing Data, and Outliers

For early withdrawal of a subject from the study, all safety assessments related to end of study visit will be performed. If the subject notifies of his/her intention to withdraw the consent, stops visiting the study physician or there will be no communication with him/her during the study, such subject may be deemed as left the study. In such case, the analysis will be done till the last available visit.

No imputation will be used for missing data, data will be presented as such.

5.10 Analysis Software

All analysis will be performed using SAS® Software version 9.4 or later.

6.0 DEMOGRAPHICS AND OTHER BASELINE CHARACTERISTICS

6.1 Demographics

Demographic characterstics including age, gender and race and anthropometric variables, height, weight and BMI will be summarized descriptively by treatment arms. Variables that are measured on a continuous scale, such as the age of the patient at the time of enrollment, the number of non-missing observations, mean, median, SD, minimum, and maximum will be tabulated by treatment assignment and overall, for the randomized population. Variables that are measured on a categorical scale will be summarized using frequencies and percentages by treatment assignment and overall, for the safety population.

6.2 Prior and Concomitant Medications

The prior and concomitant medications will be coded using World Health Organization Drug Dictionary (WHODD), 01 March 2021 or later. The frequency count and percentage of subjects will be summarized according to the coded terms of system organ class and preferred term across all the treatment groups.

6.3 Baseline and Screening Conditions

6.3.1 Baseline Medical/Surgical History

Medical and surgical history will be coded using MedDRA version 23.0 or higher. The medical history will be analyzed using frequency count and standard summary statistics based on the safety analysis set.

6.3.2 Baseline Physical Examination and Vital Signs

The observed data of physical examination will be summarized categorically (Normal, Abnormal Clinically Significant, Abnormal Not Clinically Significant) in the tabular format by treatment group. Individual data listings of each physical examination parameter (observed data) will be presented for each subject.

Vital signs will be presented with standard summary statistics and laboratory parameters at baseline will be summarized with frequency and percentages of subjects with Normal, Abnormal Clinically Significant, Abnormal Not Clinically Significant results.

6.3.3 Other Screening Assessments

Assessment of infection risk category, RT-PCR testing for COVID-19 and urine pregnancy test that are assessed at screening only. For inclusion and exclusion testing will not be presented separately. However, the frequency and percentage will be presented for patients who will be eligible to participate based on all screening conditions.

7.0 STATISTICAL ANALYSES

7.1 Primary Endpoint Analysis

The immunogenicity analysis will be performed on immunogenicity analysis set (FAS and PP) and safety analysis will be performed on safety analysis set.

In Phase II and Phase III, non inferiority margin will be considered as 0.67 as per WHO guidelines, i.e. non inferiority is demonstrated if the lower bound of 95% CI of GMT ratio (GEMCOVAC-OM/ COVISHIELD™) is > 0.67.

In Phase III, non-inferiority margin for seroconversion is defined as lower bound of 95% CI in seroresponse rate difference (Seroconversion Rate GEMCOVAC-OM - Seroconversion Rate COVISHIELD™) being > - 10%.

If Lower Limit of two-sided 95% CI calculated for non-inferiority testing is >1 for GMT ratio and >0 for difference in seroconversion in FAS population, then superiority can be interpreted though not powered.

The interim Phase III immunogenicity and safety data at Day 29 will be analysed and submitted to the office of DCGI for Emergency Use Authorization.

Phase II

<u>**Safety Analysis**</u>

- **Occurrence and severity of local and systemic reactogenicity adverse events (AEs) for 7 days following vaccination**
- **Occurrence of unsolicited adverse events up to day 29 post vaccination**
- **Occurrence of related unsolicited adverse events throughout the duration of the study**
- **Occurrence of serious adverse events (SAEs): throughout the duration of the study**
- **Occurrence of adverse events of special interest (AESI): throughout the duration of the study**

Adverse events (AE) will be coded in accordance with the MedDRA version 25.0 or higher. Number of Occurrence and severity of local and systemic reactogenicity AEs, Unsolicited AEs, related unsolicited AEs, and Serious AEs reported during the study will be presented with number and percentage of subjects who reported the corresponding events by vaccine and comparator arms.

Also, 95% confidence interval (CI) for proportion of subjects will be calculated by using the exact binomial distribution from Clopper- Pearson's method.

Immunogenicity Analysis

- **Comparison of anti-Spike (omicron variant) IgG Antibodies (GMT) at Day 29 with GEMCOVAC-OM against GEMCOVAC-19**

  The GMTs of the Anti-Spike (omicron variant) IgG Antibodies (GMT) at baseline and Day 29 will be evaluated among subjects who receive booster dose. Summary statistics of GMTs will be calculated based on base 10 log-transformed titres. GMT ratio from pre-vaccination to post-vaccination in each arm will be compared between the subjects receiving GEMCOVAC-OM and those who received GEMCOVAC-19.

  The analysis will be performed by using analysis of covariance (ANCOVA). The GMT ratio will be the outcome variable, Study treatment will be main independent variable, baseline titres will be the covariate in the ANCOVA analysis.

Phase III

- **Comparison of neutralizing antibody titers against SARS-CoV-2 (omicron variant) using plaque reduction neutralization test (PRNT) assay with COVISHIELD™ at Day 29 by non-inferiority**

  The geometric mean titres (GMTs) of neutralisation antibodies against SARS-CoV-2 at baseline and Days 29 will be evaluated among subjects who receive booster dose. Summary statistics of GMTs will be calculated based on base 10 log-transformed titres.

  GMT ratio from pre-vaccination to post-vaccination in each arm will be compared between the vaccine and comparator arms. The analysis will be performed by using analysis of covariance (ANCOVA). Baseline log-transformed titres will be adjusted as covariate in the model.

This analysis will be performed for combined population as well as separately for COVAXIN and COVISHIELD group.

- **Comparison of seroconversion rates as assessed by ≥ 2- fold rise in neutralizing antibodies against SARS-CoV-2 (omicron variant) using PRNT at Day 29 between GEMCOVAC-OM and COVISHIELD™ by non-inferiority**

  Seroconversion rate is defined as percentage of subjects who will show ≥ 2-fold rise in neutralizing antibody titers (PRNT assay using omicron variant) at Day 29 compared to baseline.

  Number and percentage of subjects who achieve ≥2-fold rise will be presented for GEMCOVAC-OM and COVISHIELD™ group. 95% CI will be calculated for percentage by using Clopper-Pearson method. Difference between percentages and the 95% CI for the difference will also be calculated by using Meitinen-Nurminen method. Chi-square or Fisher's exact test will be used to compare the subjects who achieve ≥2-fold rise between GEMCOVAC-OM and COVISHIELD™ arms.

  Non-inferiority margin for difference in seroconversion rates is considered as -10% (Seroconversion Rate GEMCOVAC-OM – Seroconversion Rate COVISHIELD™), i.e. GEMCOVAC-OM vaccine will be considered as non-inferior to the COVISHIELD™ vaccine if the lower bound of 95% CI of the difference in Seroconversion Rates is > -10%.

7.2 Secondary Endpoint Analysis

Phase II

- **Comparison of seroconversion rates as assessed by ≥ 2- fold rise in antibody titers at Day 29 from baseline**

The number and percentage of subjects who achieve ≥2-fold rise will be presented by GEMCOVAC-OM and GEMCOVAC-19 group. 95% CI will be calculated for percentage by using Clopper-Pearson method. Difference between percentages and the 95% CI for the difference will also be calculated by using Meitinen-Nurminen method. Chi-square or Fisher's exact test will be used to compare the subjects who achieve ≥2-fold rise between GEMCOVAC-OM and GEMCOVAC-19 arms.

- **Comparison of neutralizing antibodies against SARS-CoV-2 using a surrogate virus assay (cPASS™ neutralization antibody kit) at Day 29**

  The median/ mean neutralization percentage at baseline and Days 29 will be evaluated among subjects who receive booster dose. Median/ mean of percentage from pre-vaccination to post-vaccination in each arm will be compared between the subjects receiving GEMCOVAC-OM and GEMCOVAC-19. The analysis will be performed by using analysis of covariance (ANCOVA). The difference in median/ mean percentage neutralization will be the outcome variable, Study treatment will be main independent variable and baseline neutralization percentage will be the covariate in the ANCOVA analysis.

- **Cell mediated immunity assessment by cytokine expression from stimulated PBMCs at Day 29 (20% of participants)**

  Summary statistics of cell mediated immunity response will be provided by vaccine arms.

Phase III

- **Occurrence and severity of local and systemic reactogenicity adverse events (AEs) for 7 days following vaccination**
- **Occurrence of unsolicited adverse events up to day 29 post vaccination**

- **Occurrence of related unsolicited adverse events throughout the duration of the study**
- **Occurrence of serious adverse events (SAEs): throughout the duration of the study**

  Adverse events (AE) will be coded in accordance with the MedDRA version 23.0 or higher. Number of Occurrence and severity of local and systemic reactogenicity AEs, Unsolicited AEs, related unsolicited AEs, and Serious AEs reported during the study will be presented with number and percentage of subjects who reported the corresponding events by vaccine GEMCOVAC-OM against GEMCOVAC-19.

  Also, 95% confidence interval (CI) for proportion of subjects will be calculated by using the exact binomial distribution from Clopper- Pearson's methods.

- **Comparion of anti-Spike (omicron variant) IgG antibodies (GMT) between GEMCOVAC-OM and COVISHIELD™ at Day 29**

  The GMTs of the Anti-Spike (omicron variant) IgG Antibodies (GMT) at baseline and Day 29 will be evaluated among subjects who receive booster dose. Summary statistics of GMTs will be calculated based on base 10 log-transformed titres. GMT ratio from pre-vaccination to post-vaccination in each arm will be compared between the subjects receiving GEMCOVAC-OM and those who received COVISHIELD™. The analysis will be performed by using analysis of covariance (ANCOVA). The GMT ratio will be the outcome variable, Study treatment will be main independent variable, baseline titres will be the covariate in the ANCOVA analysis.

  In Phase III, non inferiority margin will be considered as 0.67 as per WHO guidelines, i.e. non inferiority is demonstrated if the lower bound of 95% CI GMT ratio (GEMCOVAC-OM/ COVISHIELD™) is > 0.67.

- **Comparison of seroconversion rates as assessed by ≥ 2- fold rise in antibody titers at Day 29 between GEMCOVAC-OM and COVISHIELD™ using test of non-inferiority**

  Number and percentage of subjects who achieve ≥2- fold rise will be presented by GEMCOVAC-OM and COVISHIELD™ group. 95% CI will be calculated for percentage by using Clopper-Pearson method. Difference between percentages and the 95% CI for the difference will also be calculated by using Meitinen-Nurminen method. Chi-square or Fisher's exact test will be used to compare the subjects who achieve ≥2-fold rise between GEMCOVAC-OM and COVISHIELD™ arms.

  Non-inferiority margin for difference in seroconversion rates is considered as -10% (Seroconversion Rate GEMCOVAC-OM – Seroconversion Rate COVISHIELD™), i.e. GEMCOVAC-OM vaccine will be considered as non-inferior to the COVISHIELD™ vaccine if the lower bound of 95% CI of the difference in Seroconversion Rates is > -10 %.

- **Comparison of neutralizing antibodies against SARS-CoV-2 using a surrogate virus assay (cPASS™) at Day 29**

  The median/ mean neutralization percentage at baseline and Days 29 will be evaluated among subjects who receive booster dose. Median/ mean of percentage from pre-vaccination to post-vaccination in each arm will be compared between the subjects receiving GEMCOVAC-OM and who received COVISHIELD™. The analysis will be performed by using analysis of covariance (ANCOVA). The difference in mean percentage neutralization will be the outcome variable, Study treatment will be main independent variable and baseline neutralization percentage will be the covariate in the ANCOVA analysis.

- **Cell mediated immunity assessment by cytokine expression from stimulated PBMCs at Day 29 (25% of participants)**

Summary statistics of cell mediated immunity response will be provided by vaccine arms.

- **B-Cell (BA.1 and BA.5) assessment at Day 29**

  Summary statistics of B-Cell response will be provided by vaccine arms.

7.3 Exploratory Endpoint Analysis

Phase II

- GMT measured by IgG-ELISA against SARS-CoV-2 Spike protein (omicron variant) post booster administration at Day 90.
- Neutralisation antibodies against SARS-CoV-2 using a surrogate virus assay (cPASS™ neutralization antibody kit) post booster administration at Day 90
- Assessment of cellular immune responses from stimulated PBMCs at Day 90 (20% of participants)
  - Summary statistics of cellular immune response will be provided by each arm
- Symptomatic laboratory confirmed COVID-19 cases throughout the duration of the study
  - Symptomatic Laboratory confirmed COVID cases will be summarized as number and percentage of subjects by each arm in each of the COVISHIELD™ and who received COVAXIN™ group.

Phase III

- GMT measured by IgG-ELISA against SARS-CoV-2 Spike (omicron variant) protein post booster administration at Day 90
- Neutralisation antibodies against SARS-CoV-2 using a surrogate virus assay (cPASS™ neutralization antibody kit) post booster administration at Day 90

- GMT of SARS-CoV-2 specific serum neutralizing antibody levels using live virus (PRNT50) assay, at Day 90
- Assessment of cellular immune responses from stimulated PBMCs at Day 90 (25% of participants)
  - Summary statistics of cellular immune response will be provided by each arm.
- Symptomatic Laboratory confirmed COVID-19 cases throughout the duration of the study
  - Symptomatic Laboratory confirmed COVID cases will be summarized as number and percentage of subjects by each arm in each of the COVISHIELD™ and who received COVAXIN™ group.
- B-Cell (BA.1 and BA.5) assessment at Day 90
  - Summary statistics of B-Cell response will be provided by vaccine arms.

8.0 SAFETY AND TOLERABILITY ANALYSES

Visit wise summary and change values from baseline up to the end of the study for safety assessments, including vital signs, body weight, physical examination, laboratory investigations will also be summarized as per standard convention. Shift table for change from baseline to EOS in categorical response will also be prepared wherever needed. Safety evaluations will be done on safety analysis set.

8.1 Adverse Events

Along with all the expected adverse events mentioned in section 7 of the protocol, any abnormal physical examination findings judged by the investigator as ‘clinically significant’ (except at screening) will be considered as an AE and will be recorded appropriately.

The AEs will be coded by system organ classification (SOC) and preferred term (PT) using the Medical Dictionary for Drug Regulatory Activities version 23.0 or later. All the AEs will be listed and categorized by AEs before dosing and after dosing. All AEs, which occur after the first dose of the study drugs, will be categorized as TEAEs i.e. AEs with onset date on or after the first dose of the study drugs. The TEAEs will be tabulated and summarized for each treatment group. All AEs will be collected, evaluated and tabulated by SOC, PT, start date, relationship to the IP, seriousness, severity, action taken, and outcome, and date of resolution for each treatment group. The analysis of all safety endpoints will be based on descriptive statistics as described above for continuous/categorical variables.

8.2 Vital Signs

Descriptive statistics will be provided by treatment group for heart rate, respiratory rate, systolic and diastolic BP, body temperature etc. The vital signs will be generated by visit as absolute values and the change from baseline will also be summarized. P value for change from baseline will be calculated using paired t-test and Wilcoxon signed rank test based on normality.

8.3 Physical Examination

The observed data of physical examination will be summarized categorically (Normal, Abnormal Clinically Significant, Abnormal Not Clinically Significant) by treatment group. Shift table for change from baseline to EOS in categorical response will also be presented for all SOCs. Individual data listings of each physical examination parameter will be presented for each subject.

8.4 Pregnancy Test

Urine pregnancy test for female subjects of childbearing potential those who are married or sexually active will be summarized pre vaccination and post vaccination.

9.0 INTERIM ANALYSIS

An interim analysis for Phase II, and Phase III of the study will be performed when all subjects complete Visit 2 (Day 29) assessment of immunogenicity. As the primary endpoint assessment is covered in the interim analysis, no adjustment for level of significance has been done in the sample size justification for interim analysis.

The Phase III interim immunogenicity and safety data will be submitted to the office of DCGI for Emergency Use Authorization.

10.0 REPORTING CONVENTIONS

10.1 Reporting of Numeric Values

All raw data will be presented to the original number of decimal places. The mean, median and quartiles will be presented with 1 decimal place more than raw data. The standard deviation (SD), Standard Error of Mean and Confidence Interval (CI) of mean will be presented with 1 decimal place more than mean. The range (minimum and maximum) will be presented as per the raw data Percentages will be presented in xx.x% format.

All categories of variables will be presented even if there is no data. Blank cells will be filled by "- "in reporting of results.

Precision of p-values will be 4 decimal places. p-values less than 0.0001 will be presented as < 0.0001 and if equal to 1 then ≥ 0.9999.

11.0 REFERENCES

[1] Omer SB, Malani PN. Booster Vaccination to Prevent COVID-19 in the Era of Omicron: An Effective Part of a Layered Public Health Approach. JAMA. Published online January 21, 2022. doi:10.1001/jama.2022.0892

[2] https://www.fda.gov/news-events/press-announcements/coronavirus-covid-19-update-fda-takes-additional-actions-use-booster-dose-covid-19-vaccines)

[3] Chemaitelly H, Tang P, Hasan MR, et al., Waning of BNT162b2 vaccine protection against SARS-CoV-2 infection in Qatar. New England Journal of Medicine. 2021 Oct 6.

[4] https://www.cidrap.umn.edu/news-perspective/2022/01/new-data-show-booster-doses-protect-against-omicron

[5] Wanwisa Dejnirattisai, Jiandong Huo, Daming Zhou, et al., SARS-CoV-2 Omicron-B.1.1.529 leads to widespread escape from neutralizing antibody responses. 2022, Cell 185, 467–484.

[6] Protocol -GBL GEMCOVAC **GBL/GEMCOVAC-OM/2022/02 V4.0**, dated 03 OCT 2022.

[7] ICH E3: Structure and content of Clinical Study Reports, November 1995,

Committee for Proprietary Medicinal Products.

[8] ICH E9: Statistical Principles for Clinical Trials, September 1998, Committee for Proprietary Medicinal Products.
